# JEDi: java essential dynamics inspector — a molecular trajectory analysis toolkit

**DOI:** 10.1186/s12859-021-04140-5

**Published:** 2021-05-01

**Authors:** Charles C. David, Chris S. Avery, Donald J. Jacobs

**Affiliations:** 1 The New Zealand Institute for Plant and Food Research, Ltd., Canterbury Agricultural Park, Lincoln, New Zealand; 2 Department of Bioinformatics and Genomics, University of North Carolina Charlotte, 9201 University City Blvd, 28223 Charlotte, NC USA; 3 Department of Physics and Optical Sciences, University of North Carolina Charlotte, 9201 University City Blvd, 28223 Charlotte, NC USA

**Keywords:** Essential dynamics, Principal component analysis, Hierarchical principal component analysis, Kernel principal component analysis, Sparse principal component analysis, Subspace analysis, Outlier detection, Rare events, Covariance shrinkage

## Abstract

**Background:**

Principal component analysis (PCA) is commonly applied to the atomic trajectories of biopolymers to extract essential dynamics that describe biologically relevant motions. Although application of PCA is straightforward, specialized software to facilitate workflows and analysis of molecular dynamics simulation data to fully harness the power of PCA is lacking. The Java Essential Dynamics inspector (JEDi) software is a major upgrade from the previous JED software.

**Results:**

Employing multi-threading, JEDi features a user-friendly interface to control rapid workflows for interrogating conformational motions of biopolymers at various spatial resolutions and within subregions, including multiple chain proteins. JEDi has options for Cartesian-based coordinates (cPCA) and internal distance pair coordinates (dpPCA) to construct covariance (Q), correlation (R), and partial correlation (P) matrices. Shrinkage and outlier thresholding are implemented for the accurate estimation of covariance. The effect of rare events is quantified using outlier and inlier filters. Applying sparsity thresholds in statistical models identifies latent correlated motions. Within a hierarchical approach, small-scale atomic motion is first calculated with a separate local cPCA calculation per residue to obtain eigenresidues. Then PCA on the eigenresidues yields rapid and accurate description of large-scale motions. Local cPCA on all residue pairs creates a map of all residue-residue dynamical couplings. Additionally, kernel PCA is implemented. JEDi output gives high quality PNG images by default, with options for text files that include aligned coordinates, several metrics that quantify mobility, PCA modes with their eigenvalues, and displacement vector projections onto the top principal modes. JEDi provides PyMol scripts together with PDB files to visualize individual cPCA modes and the essential dynamics occurring within user-selected time scales. Subspace comparisons performed on the most relevant eigenvectors using several statistical metrics quantify similarity/overlap of high dimensional vector spaces. Free energy landscapes are available for both cPCA and dpPCA.

**Conclusion:**

JEDi is a convenient toolkit that applies best practices in multivariate statistics for comparative studies on the essential dynamics of similar biopolymers. JEDi helps identify functional mechanisms through many integrated tools and visual aids for inspecting and quantifying similarity/differences in mobility and dynamic correlations.

**Supplementary Information:**

The online version contains supplementary material available at 10.1186/s12859-021-04140-5.

## Background

The widespread use of molecular dynamics (MD) simulation of biopolymers [1] has created a greater need for statistical tools to analyze atomic trajectories. A thorough analysis helps identify mechanisms responsible for biological function. Molecular conformation is represented by a vector space with dimension equal to the number of degrees of freedom (DOF), often taken as Cartesian coordinates of selected atoms. Internal DOF can also be employed, such as distances between pairs of atoms. [2, 3] Certain distance pairs may characterize a functional motion, which in some cases can be measured experimentally as illustrated in myosin. [4] Principal component analysis (PCA) is a method from multivariate statistics to reduce the dimensionality of the vector space, allowing the essential dynamics (ED) [5] of large molecules to be expressed in terms of a small number of collective motions. [3, 6, 7]

**Fig. 1:**
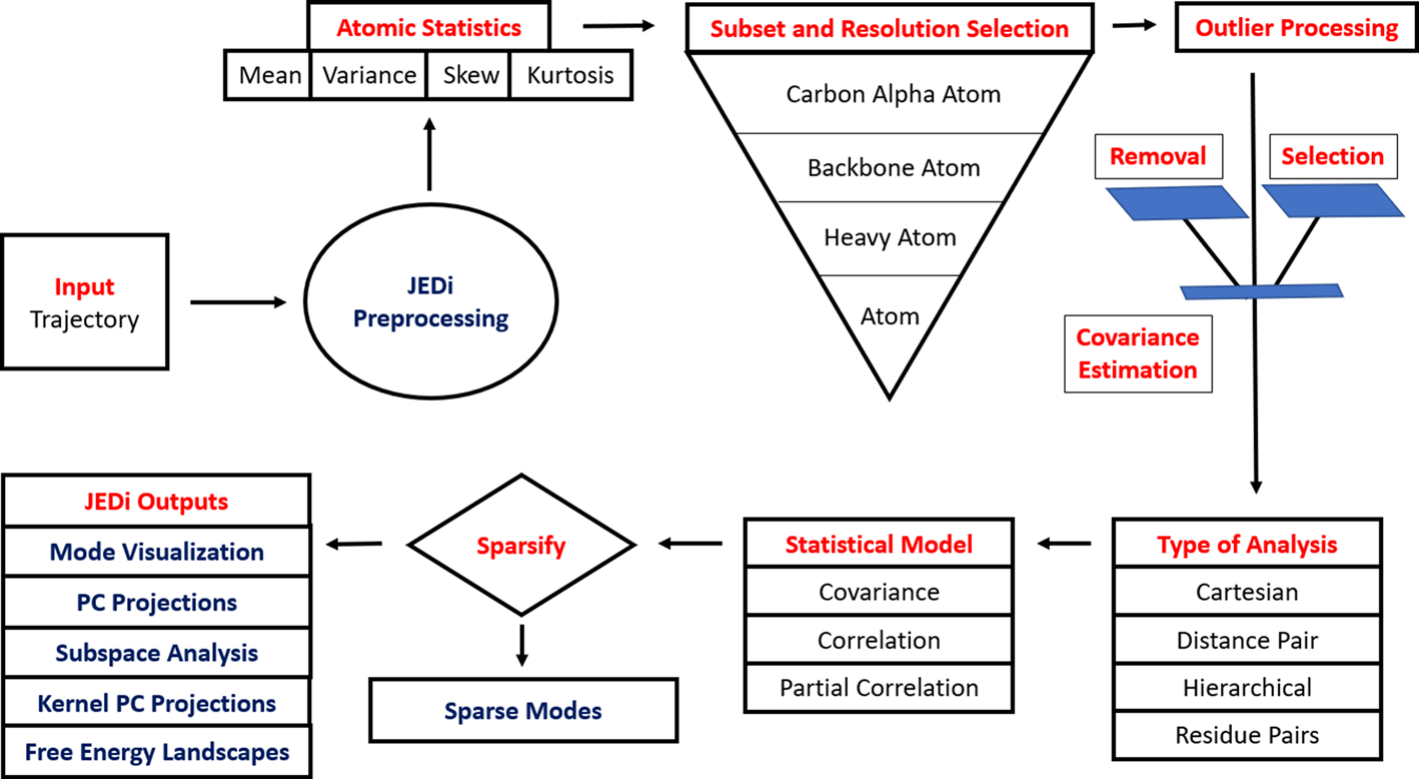
Overview of the JEDi workflow

To calculate ED one can consider using specialized software or a MD simulation program [8, 9] to perform PCA on its outputted trajectories. [10] However, MD programs that perform ED lack the sophistication for a thorough analysis. Standalone program Bio3D [11] runs in R, limiting its utility to low throughput loads. ModeTask [12] runs from a command line or as a PyMol plugin. ModeTask is used for PCA and normal mode analysis and offers good visualization, but has minimal statistical analysis functionality. The standalone software JED [13] provides a means for large scale comparative analyses and has many more statistical analysis tools than Bio3D or ModeTask, plus JED offers good visualization by providing PyMol scripts. Despite many advantages, JED requires a steep learning curve to use properly.

With Java Essential Dynamics (JED) as a forerunner, the JEDi software includes novel hierarchical PCA methodologies, more statistical tools and multithreading is added to achieve real-time analysis. JEDi handles high throughput analysis involving large numbers of input files simultaneously for comparative analyzes controlled by turning on-and-off switches from a single input file to *inspect* molecular motions in great detail.

Feedback from JED users indicated a desire to examine ED with varying resolutions controlling the number of DOF representing the dynamics. Several requests were made to improve the user-interface for making it easier to inspect the high dimensional data and statistical outputs. In addition to addressing these concerns, JEDi has new functionality to quantify the role of rare events and makes it easier to employ different statistical models simultaneously. Realizing that most tasks are repeated many times, it became clear that a toolkit to conveniently and coherently perform a comprehensive set of real-time operations is needed.

In this report, we describe a major upgrade to JED on two fronts. First, the user-interface has been completely changed to execute inspection of data using workflows that automate repetitive analysis. The previous high barrier learning curve is greatly reduced due to the simplified user-interface that makes it easy to control how to inspect the data with explicit user-options. Second, additional novel computational algorithms have been included in the package, such as hierarchical PCA, which offers unique capabilities in analyzing large systems at high spatial resolutions, sparsification of statistical matrices to extract latent correlated motions, and filters to quantify the effects of rare events. With an emphasis placed on data inspection, the JED software is now called JEDi, for Java Essential Dynamics *inspector*.

A schematic of the JEDi toolset is shown in Fig. 1. The scope of analysis tools available in the JEDi toolkit for computing and interpreting the ED of single and multiple molecular trajectories includes: (1) Specification of multiple subsets of atoms/residues with multiple levels of spatial resolution (or coarse-graining) and distributed selection of DOF; (2) Statistical moments of all variables up to fourth order, with options for selecting variables through thresholding; (3) Outlier processing including removal of outliers and selection of outliers based on thresholds, with model to model comparisons; (4) Optimal covariance conditioning; (5) Multiple types of PCA including a novel hierarchical cPCA, the determination of a coupling score between all residues in a subset, and generalizing dpPCA for any selection of atom pairs; (6) Three models of PCA using the covariance, correlation and partial correlation matrices are available with quantitative subspace comparisons; (7) More than a dozen kernel PCA analyses with PCA filtering for rapid processing are available; (8) default comprehensive graphical outputs, including PyMol scripts to visualize individual principal component (PC) modes and essential motion over user-selected time scales as movies; (9) Creation of free energy surfaces from the top two PC modes; (10) A verbose option allows flat files to be given as part of its output. The output files are compressed in bzip2 format to reduce storage requirements. Finally, JEDi is programmed with multi-threading to complete all the analyses rapidly.

## Implementation

In multivariate statistics the process of PCA is commonly applied to three types of statistical models, given by the covariance matrix, *Q*, the correlation matrix, *R*, and the partial correlation matrix, *P*. For all three statistical models, a spectral decomposition is performed. The eigenvectors are called PC modes. The rank ordering of the eigenvalues for *Q* from highest to lowest quantifies variation in collective variables.

Within a quasi-harmonic approximation [14], the PC modes from the *Q* associated with position coordinates are equivalent to the normal modes of vibration, where the largest variation corresponds to the lowest frequency motion [15]. As such, PCA on *Q* provides a description of ED similar to normal mode analysis. Applying PCA on *R* allows correlated motions to be tracked without being biased toward the large amplitude motions. Applying PCA on *P* yields correlations between variables with the effects of all other variables removed. The application of PCA on the standard statistical measures *Q*, *R*, and *P* used in multivariate statistics have been implemented previously in the context of ED. [13].

### Alignment of conformations

A molecular trajectory provides snapshots (frames) depicting a set of sampled conformations, denoted as $\{X(t)\}$ where *t* is a discrete frame index. The vector *X* may describe a subset of atoms within the system. All atomic coordinates are read in from a set of standard format PDB files. With this information, any subset of residues may be studied at various levels of atomic resolution, from all atom to alpha carbon atom only. For a subset consisting of *m* atoms, *X* will be a column vector of dimension 3*m* since each atom has (*x*, *y*, *z*) coordinates in Cartesian space.

For *n* observations, and *m* atoms the position covariance matrix associated with *x*, *y* and *z* coordinates per atom is constructed from a $3m \times n$ data matrix, *A*. The 3*m* rows define the conformation of the system and the *n* columns represent the number of frames sampled in a trajectory that describe how the molecular conformation evolves in time. The molecular conformation deforms as the center of mass and orientation of the system changes over time. Due to global translations and rotations, all frames are aligned using an intermolecular correspondence set (ICS) of atoms.

The conformation of each frame is aligned to a reference structure using the quaternion alignment method [13]. The reference structure is usually selected as one of the conformations in a trajectory. The choice of reference structure is arbitrary, however it is necessary to use the same reference structure throughout an analysis. To facilitate a comparative analysis across many systems, the aligned coordinates $\{ X_a \}$ of each conformation in each trajectory have the same atoms in the ICS aligned to the same reference structure. JEDi outputs the aligned coordinates $\{ X_a \}$ for all trajectories that are synchronized to the specified reference PDB structure, and to be use in subsequent calculations, such as performing displacement projection plots.

The *A* data matrix represents the aligned coordinates with respect to the ICS with the mean of these coordinates subtracted. Thus, $A = \{ X_a - \langle X_a \rangle \}$ Then, $Q=(AA^T)/(n-1)$, where $A^T$ is the transpose of the data matrix. The $3m \times 3m$ *Q* matrix is real and symmetric, which guarantees only real eigenvalues and real components in eigenvectors. The quadratic form of *Q* ensures all eigenvalues are non-negative. This form of calculating *Q* is quite common, as it provides a simple unbiased estimator for the population covariance matrix. When using distance pairs there is no need to align the data since the distances between pairs of atoms are invariant under translations and rotations.

### Statistical sampling

A variety of methods to quantify sampling adequacy and perform outlier detection are provided. To assess how well each variable (or DOF) is sampled, the Measure of Sampling Adequacy (MSA) for each variable and Kaiser-Meyer-Olkin (KMO) statistic are calculated [16, 17]. The problem of determining outliers in molecular dynamics (MD) trajectories is complicated because important functional mechanisms can be triggered by rare events that are not well sampled during a simulation, yet these outliers are at risk of being thrown out of the analysis. Furthermore, if some DOF are not sampled well, it is not prudent to ignore these variables altogether, although it is valuable to know where statistical inference is weak. The inclusion or exclusion of outliers have a major effect on the covariance matrix. For this reason, the best practices for covariance matrix estimation is implemented.

It is well known that the sample covariance matrix *Q*, defined above, generally provides a poor estimator for the population covariance matrix when the number of samples is not much larger than the number of variables. For this reason, an adaptive-covariance shrinkage (ACS) algorithm [18] is implemented to obtain an improved covariance estimation. The target matrix for ACS shrinkage is “*Diagonal-Unequal-Variances*”. The ACS algorithm determines the optimal shrinkage intensity based on the variance of the entries of the sample covariance matrix.

**Fig. 2:**
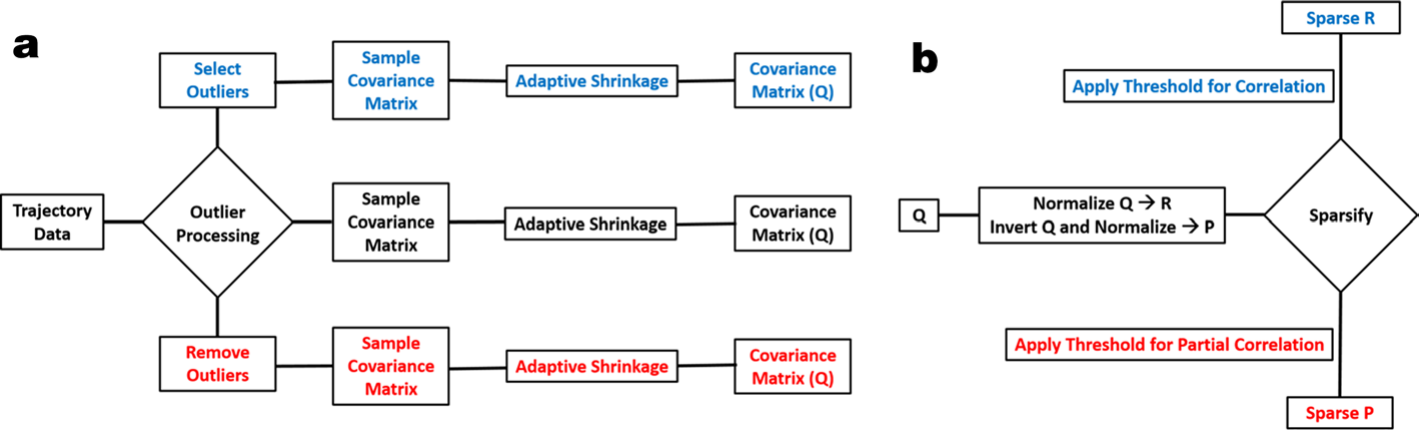
**a** Outlier processing and covariance estimation. **b** Sparsification of the R and P matrices

Previously, JED used a rare-event shrinkage (RES) algorithm that replaces detected outliers based on a user-defined threshold with the mean for each DOF. The RES algorithm is retained in JEDi because it offers the advantage that the user can dramatically increase shrinkage by setting a low outlier threshold, and effectively turn off shrinkage by setting a high outlier threshold. The combination of RES and ACS methods yields robust unbiased estimates for the covariance matrix. See Fig. 2a. The accurate estimation for *Q* translates to accurate estimates for *R* and *P* because the correlation and partial correlation matrices derive from the covariance matrix. With shrinkage, subsequent spectral decomposition of the statistical models yield more reliable insights into the essential subspaces due to improved consistency as quantified by cross-validation when using subsampling.

### Matrix conditioning

The inverse of *Q* is used when calculating the partial correlation matrix. For the inverse of *Q* to exist, zero eigenvalues from the spectral decomposition of *Q* cannot occur. It is important to assess the physical relevance of the smallest eigenvalues of *Q* in terms of the last significant digit of the data. Since the data is obtained from PDB files that record position coordinates to 3 decimal places, any uncertainty (via standard deviation) that is less than one thousandth of an Angstrom is below baseline noise. To accommodate this physical reality, a default floor threshold of $10^{-6}$ Angstrom-squared is applied to the eigenvalues of *Q*. After a spectral decomposition is made for *Q*, the *Q* matrix is reconstructed through outer products of its eigenvectors where all eigenvalues less than the floor threshold are replaced with the threshold value. This reconstruction procedure is an improvement over a similar method to regularize the covariance matrix when an accurate inverse of *Q* is needed. [19] Using the physically based floor thresholding procedure, the inverse of *Q* (called the precision matrix) always exists, and is then used to calculate *P*.

### Characterization of essential dynamics

Spectral decomposition yields eigenvectors, each with an eigenvalue, that define a complete set of orthogonal collective modes. When eigenvalues from *Q* are plotted against mode index sorted from highest to lowest variance, a “scree plot” typically appears indicating a large fraction of molecular motion is captured using a small fraction of modes. These modes define an “essential subspace” that describes the motions with largest amplitude. This processing has become standard practice, and like JED, JEDi offers all the usual outputs, such as scree plots, plots for the mean square fluctuations (MSF) of each PC mode and the combined MSF from the top set of modes specified by the user.

When large-scale motions underlie biological function, such as a hinge-bending motion between domains, the essential subspace captures functional dynamics. However, when functional motion has an amplitude less than dominant motion that is unrelated to function, such as a swinging C-terminus not tied to function, then the top modes of *Q* will not be biologically relevant. For this reason, JEDi allows users to apply PCA to subregions within a system. By selecting subsets of atoms, which need not be contiguous, biologically relevant motions are possible to identify, which would otherwise be missed when analyzing the entire system. In addition, the statistical models of *R* and *P* provide different insights that *Q* does not offer.

For *R*, PC modes with eigenvalues greater than 1 suggest potentially biologically relevant correlated motions. The influence from an increasing number of the original variables occurs as the eigenvalue increases. For *P*, the maximum eigenvalue is 2. Due to the nature of the partial correlation, there will be many variables with eigenvalue near 2. [20] The PC modes from *R* elucidate where correlations are present between variables, while the PC modes from *P* point to the variables that enhance or dampen the correlated motions. The comparison of the *R* and *P* matrices can help identify ‘suppressor’ and ‘activator’ variables in the subset. [20–24]

After spectral decomposition is performed on the *Q*, *R*, and *P* statistical models, the aligned conformations are projected onto the selected top modes to create scatter plots. These scatter plots either show displacement vector projections (DVP) where the origin is defined by a user-selected conformation, or principal components where the origin is at the mean position taken over all conformations in the ensemble. These scatter plots allow for the inspection of the collective dynamics in two-dimensional cross-sections of conformational space to facilitate comparisons. JEDi outputs this information graphically, where each quarter of the trajectory has a distinct color so that the evolution in time can be traced. In summary, JEDi offers convenient tools to quantify how similar/different the essential subspaces are comparatively based on the top modes of each type of statistical model.

### Outlier and inlier filtering

A comparison technique of examining the difference in the essential subspaces derived from first removing outliers and then selecting them characterizes the effects of rare events. The procedure sets a z-score threshold on each variable, or optionally a MAD score [25]. Within each frame, looking at each variable, if the value of a variable is below a set threshold, then that variable is said to be an inlier, otherwise an outlier. Using the same RES method of replacing the identified inlier or outlier by the mean (or median) of the variable, two distinct covariance matrices are constructed associated with inliers and outliers. The different results from both covariance matrices, as well as the two *R* and two *P* statistical models that derive from each covariance matrix are subsequently compared using any of the available tools such as subspace comparison. Difference in the essential motions of the inliers versus the outliers glean insight into how the rare events influence molecular function.

### Sparsification of statistical matrices

To interrogate the characteristics of correlated motions, thresholding is available for the sparsification of the *R* and *P* matrices. See Fig. 2b. The user can set thresholds separately for these analyses. The process of sparsification sets matrix entries that are below the threshold to zero. A sparsified matrix accentuates the correlated motions by diminishing the effects of motions with low correlations, since these low values are susceptible to noise. Subsequent spectral decomposition of these sparse matrices provides a clear view of the correlated dynamics within the selected region of interest.

When prompted, JEDi performs PCA analysis on both the original (unaltered) and sparse matrices, and then compares the resulting subspaces quantitatively. Moreover, JEDi will allow the user to compare corresponding entries in *R* and *P*. When the absolute value of the difference in these entries is greater than a user defined threshold, the correlation between two variables is either being enhanced or suppressed by other variables (DOF). JEDi generates a 2D map of where corresponding matrix elements in *P* are greater or lesser than that within *R*, and exceed the set threshold. This map identifies the interactions that *activate* or *suppress* correlated motions. This feature can be used on the *P* and *R* matrices or any of their sparsified versions.

The key to identifying important interactions between variables is in setting appropriate thresholds in the absolute differences between the entries in the *P* and *R* matrices, and examining the direction of the difference under different levels of sparsification. Using sparsified matrices with a variety of thresholds can therefore glean insight into physical pairwise interactions that might be relevant to molecular function.

### Trade-off in spatial and statistical resolutions

An effective statistical analysis requires balancing the level of spatial resolution with the DOF intrinsic to the data. Consider a residue with twenty atoms, ten heavy atoms, four backbone atoms and one alpha carbon atom. Note that a heavy atom is a non-hydrogen atom. In this case, there are 60 DOF available. If the all-atom level of resolution is selected, then all 60 DOF are considered. At the heavy atom resolution 30 DOF is considered. A backbone analysis (defining N-C-C-O atoms) deals with 12 DOF. An alpha-carbon analysis uses only 3 DOF. Not all DOF are equally informative to ED. For example, the 3 DOF for the alpha carbon atom is commonly used because it tracks the overall motions of a residue well, although information about conformational changes within the residue is lost.

Increasing the spatial resolution allows greater detail in conformational motion to be explored, but statistical resolution decreases as more DOF are considered for the same number of frames sampled. Besides lowering statistical inference, increasing spatial resolution increases the computation time, where the CPU time to perform spectral decomposition scales as $DOF^3$. The other complication of tracking more data is having a higher dimensional dataset that needs to be interpreted. In general, increasing spatial resolution decreases statistical resolution and vice versa. Therefore, JEDi gives the ability to select subregions within a system with specified spatial resolution to optimize this trade-off for elucidating functional mechanisms.

### Hierarchical PCA and distributed DOF

To concurrently increase spatial and statistical resolution, a novel *hierarchical* PCA (HPCA) is employed. To our knowledge HPCA was proposed as covariance splitting [26], but not implemented in the same way. Here, HPCA uses residues to define a complete set of building blocks for distributed DOF. Each residue is allotted a specified number of DOF (*h*) that represent local conformational motion of either all atoms or all heavy atoms within a residue. These are *distributed* DOF that encompass the entire set of specified residue atoms, obtained by applying cPCA to each residue yielding a set of eigenvectors, which we call an *eigenresidue*, and a set of PCs (generalized coordinates), which we call *residuePCs*. Those residuePCs are then used to construct a new covariance matrix, which when factored and convoluted with the eigenresidues yields an atomic level approximation of the ED.

For this method to work, it is required that the same aligned conformations are used for the entire region of interest as well as for each residue within that region. In other words, a single coordinate system must be used for the entire calculation. This also ensures the results will be consistent with a direct brute force approach. Using a single global alignment, a set of residuePCs is stored for each residue. The number of residuePCs used to represent a residue defines the number of DOF for that residue. The residuePCs are a set of generalized coordinates representing the internal motions of a residue, where the top modes capture most of the large-scale motions. It is worth mentioning that if all residuePCs for each residue are used, HPCA will give exactly the same results as a standard cPCA approach, and it will require approximately the same CPU time.

Due to the nature of covariance PCA, the top PC modes (i.e. most relevant eigenresidues) capture the dominant atomic motions that occur locally within each residue. As such, the desired spatial resolution is easily controlled, where a smaller *h* yields a faster calculation with less spatial resolution. The entire system, or a selected sub-region of interest, is represented by an incomplete basis set of eigenresidues. Each DOF corresponding to an eigenresidue encodes distributed information throughout the residue. Dropping *DOF* means information is discarded in a conceptually similar way as when the carbon alpha atom is used to represent an entire residue within a protein. However, unlike the carbon alpha approach, the most relevant conformational motion that takes place within each residue is prioritized in the top most eigenresidues. As such, diminishing returns sets in as *h* is increased.

With HPCA, an all-atom resolution ED analysis is possible for thousands of residues. For example, a protein with *m* residues using $h=3$ (i.e. 3 eigenresidues per residue), captures 3 distributed DOF per residue. This $3m \times 3m$ covariance matrix is the same size as a carbon alpha approach. Within common practice of considering only the top PC modes with greatest variance as indicated by a scree plot, HPCA provides an excellent approximation. Moreover, the calculation time for HPCA will be substantially faster and allow for a substantial reduction in memory usage.

When analyzing the dominant large-scale motions in large proteins, it is not required to retain atomic level detail. In this case, setting $h=1$ provides sufficient accuracy. A user can override the $h=3$ default, setting *h* to satisfy specific needs. As a general rule of thumb, *h* should be small for large systems, and made larger for smaller systems.

### Residue-residue coupling map

Eigenresidues are also employed to characterize residue-residue coupling. For two or more residues, a user selects a set of residues of interest. At the all-atom level, this residue set defines a subregion where alignment is done separately for every residue pair within the selected region, and then all eigenresidues are calculated for each residue separately. For each pair of residues, an *eigenresiduepair* is constructed from the eigenresidues for each participating residue, and the associated PCs, called *residuepairPCs* are generated.

Selecting *h* DOF (residuepairPCs) per residue leads to a $2h \times 2h$ mode coupling covariance matrix (MCCM). After performing an eigenvalue decomposition on the MCCM, the 1st and 2nd half of the components of a PC mode from the MCCM respectively correspond to the 1st and 2nd eigenresidues in a given residue pair. A scoring function is introduced to quantify the degree of participation of each residue each PC mode of the MCCM.

The mean square fluctuation (MSF) for each component of a PC mode is calculated. Since each mode is normalized, the sum over MSF over all components is 1. Summing MSF over the 1st and 2nd half of the components of the *k*-th mode leads to $w_1(k)$ and $w_2(k)$ weights, where $w_1(k) + w_2(k) = 1$. These weights give the respective fraction of participation of residue 1 and 2 in the *k*-th mode. When $w_1(k)$ is near 50%, there is strong mode coupling between the residues. Mode coupling strength decreases as $w_1(k)$ deviates farther from 50%. The scoring function is defined as $s = \sum _{k=1}^h g( w_1(k) - w_1(k) ) \lambda (k) / TR(MCCM)$ where $\lambda (k)$ is the eigenvalue of the *k*-th PC mode of the MCCM, *TR*() is the trace operation, and *g* is a Gaussian probability density centered at 0, with standard deviation set at 0.25. Note that the range of the difference, $(w_2 - w_1)$ is between -1 and 1. This scoring function smoothly quantifies the degree of mode coupling per mode, weighted by the percent variance in the data that the mode captures.

The scoring function for mode coupling strength has dependence on *h*. The most relevant information that impacts the scoring function comes from the top eigenresidues, which is why increasing *h* beyond the scree point leads to marginal change in scores. However, rapid convergence of the sum over modes does not occur because the nature of the modes change as the size of MCCM increases as *h* increases. Nevertheless, the score slowly converges with stable results generally occurring for $h>9$. Being that the calculations are extremely fast, $h=12$ is used as a default to ensure qualitatively consistent results are produced. A heat-map image of the matrix showing the propensity of residue-residue coupling for all residue pairs in the selected subregion is given as output. The user can change *h* to monitor the sensitivity of the results.

### Summary of dependencies and features

The Java code for JEDi can be downloaded from: (https://github.com/charlesdavid/JEDi).

Key resources include executable JAR files, input files, and a User Manual (Additional File 1). Additional resources are provided regarding PCA, essential dynamics, and example datasets. JEDi is written in Java. The machine on which JEDi is to be run should have JRE version 1.8 or higher installed. The programs can be run from compiled source or from the provided executable jar files.

### Dependencies

The following external libraries are required and are packaged with the JEDi program:

- JAMA Matrix: Jama-1.0.3.jar
- Java Commons: jcommon-1.0.23.jar
- Apache Commons Compress: commons-compress-1.19.jar
- JFreeChart: jfreechart-1.0.19.jar, jfreechart-1.0.19-experimental.jar, jfreechart-1.0.19-swt.jar
- PDF Estimator: estimatePDF.jar

### Features

1. *Multi-threading*. The JEDi_Driver_MT.java class instantiates all methods to run the JEDi tookit using multi-threading. The user must allocate CPU and memory resources.
2. *Task management*. To inspect high dimensional data relies on specifying which types of analysis to perform, and associated analysis parameters. The task of setting up a JEDi run is made simple by having the JEDi driver class read in a single input file that contains all needed information.
3. *User manual*. Details of how to use each task in JEDi with recommendations on how to apply the methods are provided in the JEDi user manual.
4. *Prepocessing step*. A preliminary run generates a JEDi formatted coordinate matrix file for all atoms in the PDB files that are read in as trajectory data. This initial step makes subsequent subset analyses much faster to perform. It also serves to guarantee that the specified atoms/residues for subset selection are correctly represented in matrix form. After this step, the intial PDB files are not used, except for the reference PDB file used for performing coordinate alignments. Input highlights for the preprocessing step are:
  The PDB files may be single chain or multi chain, in standard format.
  The PDB files may be uncompressed, zipped, gzipped, bzipped, or tarred.
  JEDi will process PDB records with header ‘ATOM’ or ‘HETATM’ only.
  Output highlights for the preprocessing step are:
  Variable statistics plots: mean, variance, skew, kurtosis
  Matrix of atomic coordinates before and after the optimal alignment is performed (when doOutputCoordinates=true)
  Conformation RMSD and residue RMSF.
  The B-factors in a PDB file, replaced with residue RMSF.
5. *Analysis types*. The user can specify multiple subregions (**subsets** of atoms) for analysis using different levels of **resolution**, and different **types** of PCA. Analysis type highlights are:
  *All Atom* $\rightarrow$ all atoms in the PDB.
  *All Atom Hierarchical*
  *Heavy Atom* $\rightarrow$ all atoms except hydrogen.
  *Heavy Atom Hierarchical*
  *Backbone* $\rightarrow$ 4 backbone atoms (N-C-C-O).
  *Alpha Carbon* $\rightarrow$ $\text{ C}_\alpha$ atoms only.
  *Atom List* $\rightarrow$ user defined atoms.
  *Distance Pair* $\rightarrow$ user defined pairs of atoms.
  *Individual Residue* $\rightarrow$ all atoms within residue.
  *Residue Pair* $\rightarrow$ all to all coupling scores.
6. *Statistical models*. Selection of three **model** types:
  Covariance (always performed)
  Correlation
  Partial Correlation
7. *Visualization*. The user can choose the number of most relevant modes to retain and visualize by subset. Two types of visualization are possible:
  Individual mode dynamics.
  Dynamics of selected top modes combined.
8. *Data exploration*. There are many parameters that the user can adjust to change the characteristics of the analysis during the inspection process. The outputs of these analyzes informs the user about the nature of the essential dynamics to help elucidate the mechanisms behind biologial function. Input highlights for customizing inspection are:
  Dimension of ED $\rightarrow$ number of top modes.
  Hierarchical PCA $\rightarrow$ number of eigenresidues.
  Outlier processing $\rightarrow$ Z-score or MAD score.
  Atom subsets $\rightarrow$ variance, skew, kurtosis.
  Down sampling $\rightarrow$ to strobe frames.
  Frame selection $\rightarrow$ for basin analysis.
  Sparsification $\rightarrow$ *R* and *P* thresholds.
  Kernel PCA $\rightarrow$ types and parameters.
  Free energy surfaces $\rightarrow$ smoothing level.
  Verbosity $\rightarrow$ output file types.
  Output highlights for data inspection are:
  Mean, variance, skew, kurtosis per variable.
  MSA scores and KMO statistic.
  Statistical models $\rightarrow$ plot *Q*, *R*, *P* matrices.
  All eigenvalues for *Q*, *R* and *P*.
  Scree and cumulative percent variance plots.
  MSF per mode $\rightarrow$ unweighted and weighted.
  Reduced matrices $\rightarrow$ atom to residue.
  Displacement vector projections (DVP).
  Topmost PC modes $\rightarrow$ *Q*, *R* and *P*.
  High quality PNG images $\rightarrow$ default output.
9. *Subspace comparisons*. A powerful suite of tools are available to make quantitative subspace comparisons between different statistical models and selected subregions. Output highlights are:
  Cumulative overlap (CO).
  Root Mean Square Inner Products (RMSIP).
  Comparison to random basis per subspace.
  Canonical principal angles (PA). [27]
  Comparisons between statistical models.
10. *Standalone drivers*. Additional Java programs can be run to perform comparative analysis or additional analyses. These programs are:
  VIZ_Driver: Individual and Essential motions from Q, R, and P results can be generated for any user-selected window of PC-modes, corresponding to observing molecular motions on different time scales with fine control of parameters.
  POOL_Driver: Pools together multiple trajectories into a single dataset to facilitate another JEDi analysis on the collection of data.
  SSA_Driver: Runs comparisons between a pair of trajectories. The outputs are CO, RMSIP and canonical PA.
  FES_Driver: Creates a free energy surface for any two user-selected PC-modes.
  KPCA_Driver.java: Performs kernel PCA analysis with option to select kernels and use PCA output or raw data.

**Fig. 3:**
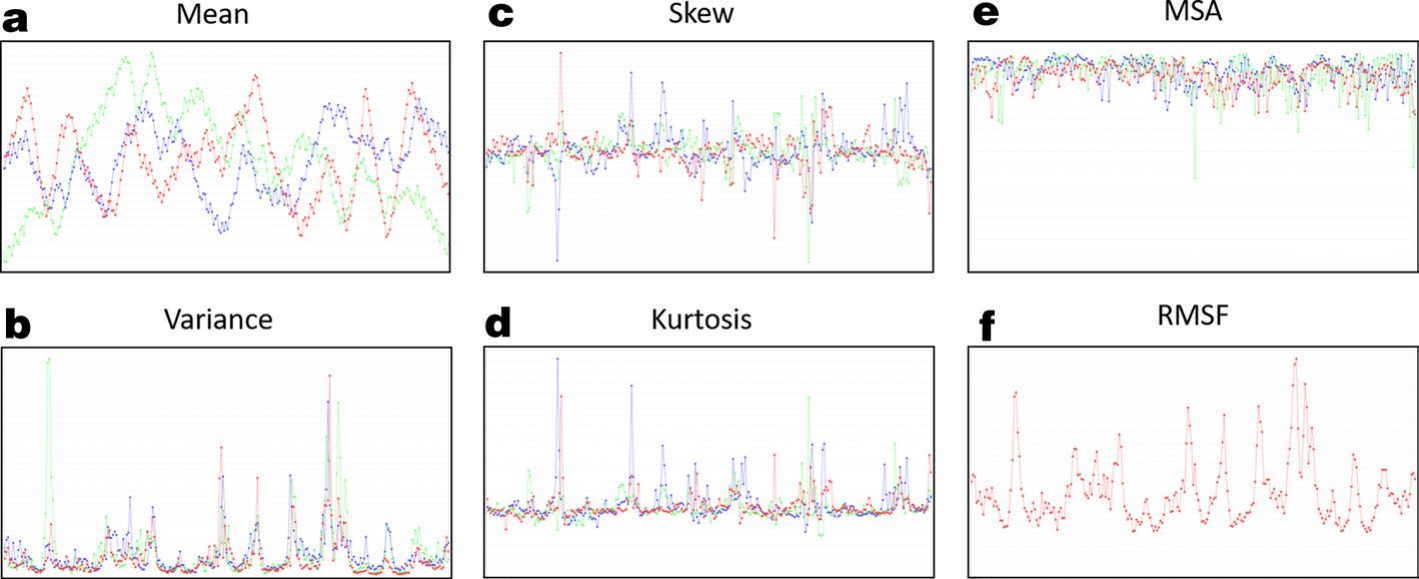
**a**–**d** show the first four moments as JEDi outputs for immediate inspection when running the analysis driver over a trajectory. These can be used to evaluate the general statistical behavior of atoms and select subsets of residues for further analysis. In **e** the MSA for each atom is shown which is also output for inspection during analysis run. Finally **f** shows RMSF, a common metric for evaluating MD Simulation behavior

## Results

Key features of JEDi are illustrated by analyzing MD trajectories for two beta-lactamase proteins. The results presented here were generated on a high performance computer (HPC). With the possible exception of analyzing all-atom models of a whole biomolecule, typical calculations by users are accessible on a modern laptop computer. However, running JEDi on a HPC takes full advantage of multi-threading. A user wishing to perform multiple analyses in the same run may request as many processors as there are analyses to run simultaneously. This allows users to inspect data in various ways in real time with what if scenarios.

### MD simulations

The simulations used for illustrative purposes are all-atom molecular dynamics simulations of TEM-1 and TEM-52 beta-lactamase. The simulations were performed for 500 *ns* each using the GROMACS MD simulation software, generating 10, 000 conformations per trajectory. Details for the parameters and protocols for the simulations have been published previously. [28]

### Running JEDi

Packaged as a Java library, JEDi is also distributed as JAR files. Each standalone JAR takes a parameter file as input. The main JAR file, JEDi_Driver_MT.jar, reads the main parameter file that consists of a set of key value pairs specifying which analyses to run, data files to read, and parameters for each analysis. The JEDi parameters file is designed for ’plug and play’ functionality. This format makes it easy to manually edit the file via command line and automate editing via scripts for high-throughput applications. The main input file directs JEDi to perform preprocessing, run all methods on all selected models and do all selected analysis, as well as modifies user serviceable settings.

Output of JEDi is routed to directories that are created during execution of the program. In the top level of the output directory information about the statistics of each subset can be found, as well as trajectory files if they are chosen to be output. Each analysis performed is handled by a distinct thread assigned from a thread pool in a multi-threaded environment, which directs output to a sub-directory where each statistical model is assigned to its own directory. This ensures threads do not clash and output is cleanly segregated. For each analysis, JEDi provides the raw data in a compressed bzip2 form for efficient memory storage, as well as high quality PNG files that provide instant access to results for users to consider without the need to use third party plotting software.

**Fig. 4:**
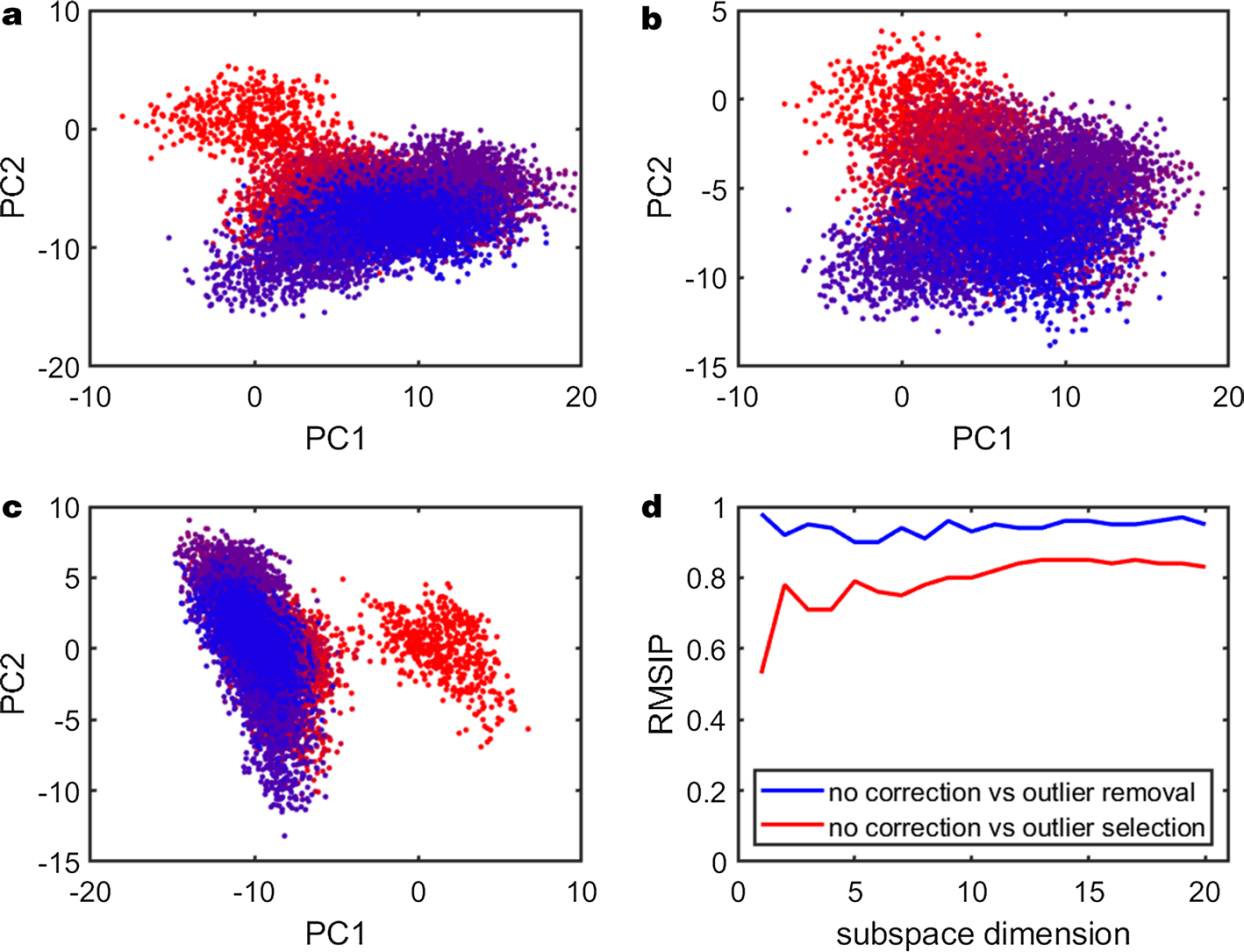
The differences in the essential dynamics of TEM-1 is visualized using **a** no outlier correction, **b** outlier removal, and **c** outlier selection. Outlier processing was done using a z-score cutoff of 1.96. Projections are colored by time series, with red being the beginning of the trajectory and blue being the end. Figure **d** shows the RMSIP between the essential subspaces of the outlier removal and selection modes with the no correction modes. We see that the essential subspace is very similar ($> 0.9$ identity) for no correction and outlier removal indicating that the outlier do not appreciably impact the essential dynamics of the protein. The RMSIP between the no-correction and outlier selection space is much lower and in the projections we see that the first PC mode is dominated by a relaxation motion of the molecule at the beginning of the simulation

### Workflow

A JEDi workflow consist of two steps. First raw structure information is converted into convenient matrix format in the preprocessing step. JEDi can read and write data in the standard PDB format. In the preprocessing step all PDB files found in a specified directory are parsed and converted into a trajectory matrix. A full path name can be given for the reference PDB file, which is used for methods that require coordinate alignment. Multiple trajectories from different files can be placed in different directories. All trajectories are aligned to the same reference structure using atoms in the ICS shared among all frames to preserve interpretability. When comparing multiple trajectories, JEDi can combine them into one for comparative analysis via the pooling driver (with optional down sampling). Importantly, trajectories can only be pooled if they share identical atoms, and they must be listed in the same order within the PDB files.

The second step of JEDi analysis is to perform PCA. JEDi supports cPCA at the Alpha Carbon, Backbone, Heavy Atom, and All Atom level, Hierarchical PCA at the Heavy Atom and All Atom level, dpPCA between specified atoms, Individual Residue PCA, and Residue Pair Coupling Analysis. All of these can be run in the same job in a multi-threaded way for efficient computation. In addition to PCA, post processing of the PCA outputs is performed, including subspace analysis, kernel PCA, construction of Free Energy Landscapes, visualization of the ED through individual modes or the superposition of modes. The schematic of the JEDi toolset is illustrated in Fig. 1.

**Fig. 5:**
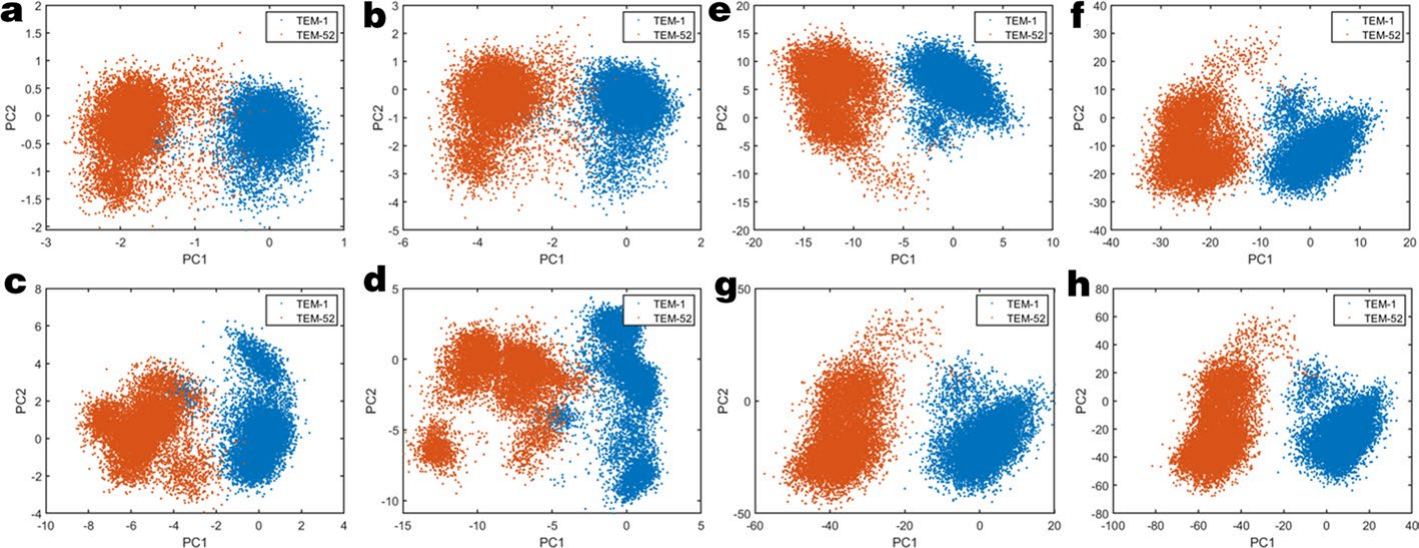
The projections of the mechanistic site conformations onto the top 2 principal components for **a** alpha carbon **b** backbone **c** heavy atom and **d** all atom levels. With increasing resolution of atomic detail there is an accompanying increase in projection detail for this small subset. In contrast **e**–**h** shows the same funnel for the ICS set which does not

### Statistics

Statistical moments up to 4th order are calculated for each position coordinate, and visualized in high-quality PNG output files for quick access. Examples of these statistics are shown in Fig. 3. Each graph provides a distinct line for x, y, and z components and are plotted against atom number. These moments inform the user about how to select subsets of atoms to analyze. The user can specify distinct thresholds for variance, skew, and kurtosis to construct atom subsets in which only the atoms that meet or exceed those thresholds are included.

Outlier and inlier filtering is provided to investigate the effects of rare events. Statistical models are built by either including only inliers or only outliers. PCA analyses is performed on the statistical models, which will be different between inliers and outliers. These differences can be quantified by subspace comparison. An example of this procedure is shown in Fig. 4. The TEM-1 MD Simulation trajectory was analyzed using outlier processing to asses the quality of the simulation. The outlier analysis indicated the presence of rare events at the very beginning of the trajectory, which is likely tracking relaxation in the simulation. For a successful outlier processing, z-score or MAD thresholds should be chosen judiciously to ensure proper sampling in both the inlier and outlier sets. Conservatively, a z-score range of [0.675, 1.96] is recommended.

**Fig. 6:**
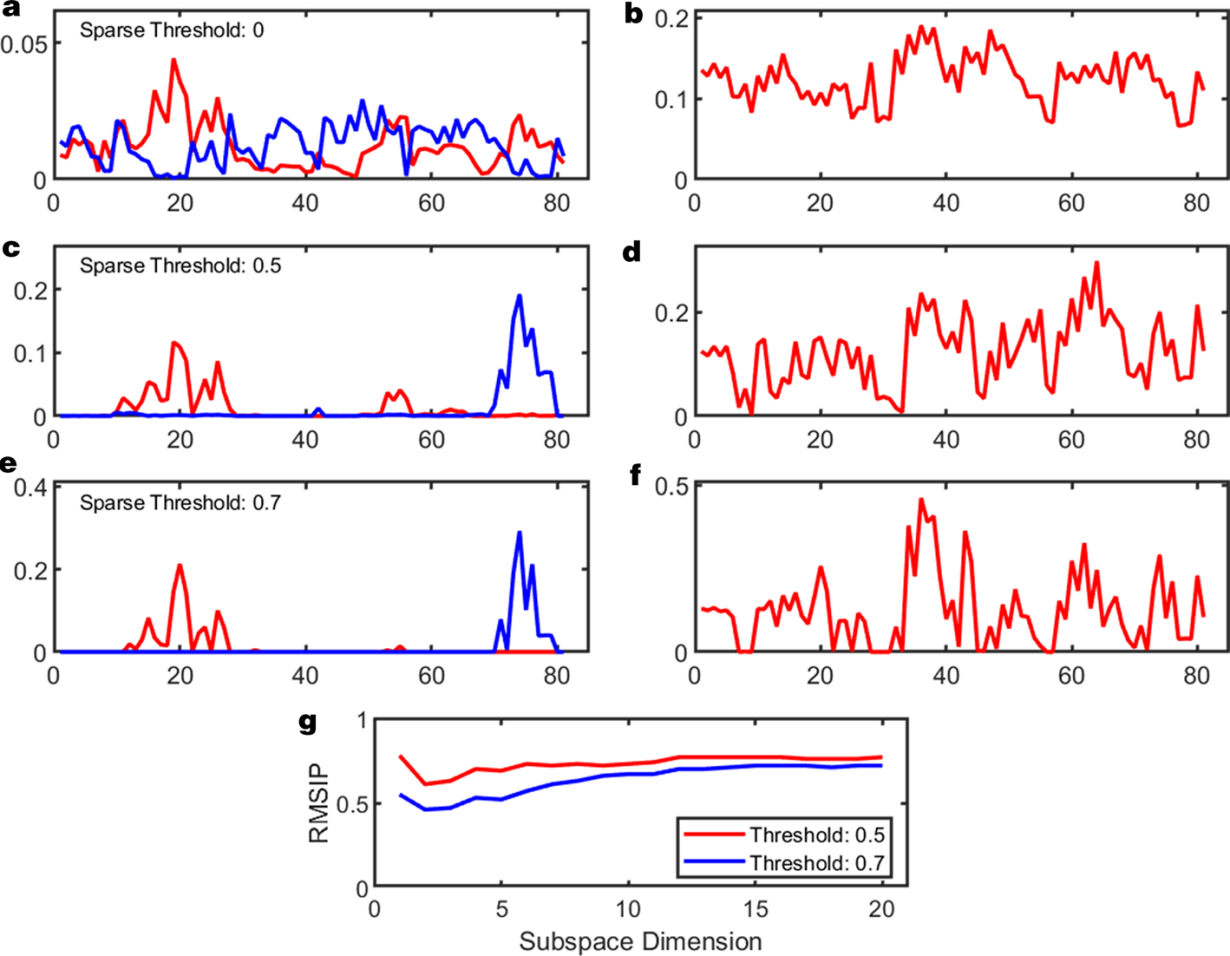
Correlation analysis shown with increasing sparsification threshold. **a**, **c**, and **e** shows the top two squared modes from each analysis with thresholds of 0.0, 0.5, 0.7 respectively showing how the information encoded in each mode becomes more localized with increasing sparsification. **b**, **d**, and **f** show the total root mean square fluctuations (RMSF) of the subset captured by the top ten modes from PCA. These indicate that while modes become more localized, the total information in the essential subspace is roughly conserved. Finally, g) shows the RMSIP for the sparsified analyses compared to the un-sparsified analysis

### Multiple resolution analyses

Multiple resolution analyses are illustrated by pooling the trajectories of TEM-1 and TEM-52 beta-lactamase via the Pooling Driver. To examine the effect of multiple levels of descriptions of molecular dynamics, a subset of 5 residues that comprise the mechanistic site is considered. This subset is specified by passing a residue list to JEDi containing only the desired residue numbers. The selected residues are SER70, LYS73, SER130, GLU166, and LYS234 [29]. cPCA on *Q* was performed on this subset of the trajectory using alpha carbon, backbone, heavy atom and all-atom resolutions.

**Fig. 7:**
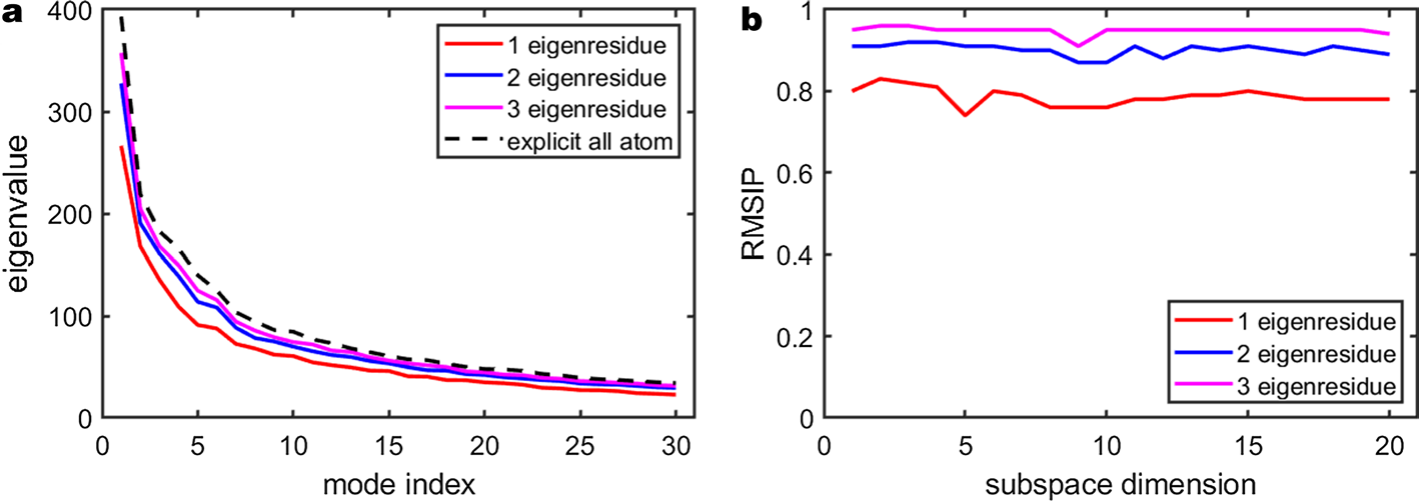
**a** For the entire TEM-1 protein, the scree plot for explicit all atom PCA is compared to HPCA using 1, 2, 3 eigenresidues to show that the captured variance approaches that of the exact variance. **b** Iterated RMSIP for HPCA is shown for 1, 2, and 3 eigenresidues compared to the eigenvectors found by explicit all atom PCA analysis. Using only a single eigenresidue per amino acid already reconstructs the explicit analysis up to 80% and including more degrees of freedom improves this to 95%

Projections of subregion motions onto the top two PC modes are shown in Fig. 5a-d for the four resolution levels. As the level of atomic resolution increases there is a corresponding increase of resolution in the projection space. Notably, there is a large difference between the alpha carbon and backbone subsets (Figs. 5a, b) in comparison to the heavy atom and all-atom subsets (Figs. 5c, d). At higher resolution, multiple clusters appear in the projections of each molecule’s trajectory, indicating dynamic transitions between meta-stable conformation states. These basins are not discernible at low resolution. The ability to probe small regions at high resolution helps reveal basins that govern molecular function. Interestingly, the overall differentiation in TEM-1 versus TEM-52 dynamics is maintained at all resolution levels.

Next consider multiple resolution analysis using PCA when determining large-scale motions of a protein. Figure 5e–h shows the same resolution funnel for the ICS residues within the entire protein. There is no appreciable gain in resolution at the global scale from an all-atom analysis. This result occurs because large conformational motions of the entire protein eclipses the small amplitude motions observed at high resolution. As expected, the alpha carbon or backbone PCA is useful for global analysis, while a targeted heavy atom or all-atom analysis is useful on a small subset of residues to extract mechanistic information.

### Sparsification

Sparsification of the R and P normalized statistical matrices allows for clarification of atomic motion in the top square modes. When sparsification thresholds are chosen that maintain moderate RMSIP scores but remove many minimally correlated interactions, it becomes possible to see exactly which atoms are contributing to key collective motions that may indicate molecular function.

The effect of sparsification on the motions of the mechanistic residues of TEM-1 is shown in Fig. 6. Figures 6a, b show the top two squared modes and the RMSF over the top 10 modes from PCA using the normal (not sparsified) correlation matrix. The top squared mode indicates that the atoms in the second residue, LYS73, play an important role in the correlated motions but the essential dynamics are spread across many of the atoms in the subset. This is indicated in the broad distribution of RMSF.

Figure 6c, d and e, f show the same information, but the PCA was performed on a sparsified correlation matrix with thresholds of 0.5 and 0.7 respectively. With increasing sparsification the background noise in the squared modes decreases. Interestingly, it visually becomes apparent the atoms participating in the most highly correlated motions occur in residues LYS73 and LYS234. The RMSF plots over 10 modes indicate that the total information contained in the essential subspace is approximately conserved independent of the sparsification threshold. This is quantifiable through RMSIP between PC modes with sparsification and normal PC modes, as shown in Fig. 6g. For higher thresholds the overlap for the individual modes are small but as the subspace dimension increases the RMSIP increases again. The sparsified essential dynamics are also visualized directly on the molecule via pymol scripts generated by JEDi’s visualization driver. Movies of the motions discussed in this section are provided in Additional File 2.

### Hierarchical PCA

To illustrate the equivalency and advantages of hierarchical PCA (HPCA), an explicit all-atom PCA and all-atom HPCA using 1, 2, and 3 eigenresidues for the entire protein were done and then compared. A subspace analysis was performed to evaluate the similarity of the results. Figure 7a shows that with an increasing number of eigenresidues used in the HPCA computation, the total variance of the essential subspace approaches the true variance as computed with a brute force all atom PCA.

The RMSIP from the subspace comparison between the explicit all-atom and all-atom HPCA is shown in Fig. 7b. The high ($> 0.8$) RMSIP between the two subspaces indicates that even a single eigenresidue is able to capture the global motions of the protein. Accuracy increases as the number of eigenresidues increases. For the same computational cost of an alpha carbon analysis, using three eigenresidues yields a very high ($> 0.95$) RMSIP to the explicit all atom approach. This example clearly shows that HPCA is an excellent approximation for a brute force all atom PCA while significantly reducing compute times.

**Fig. 8:**
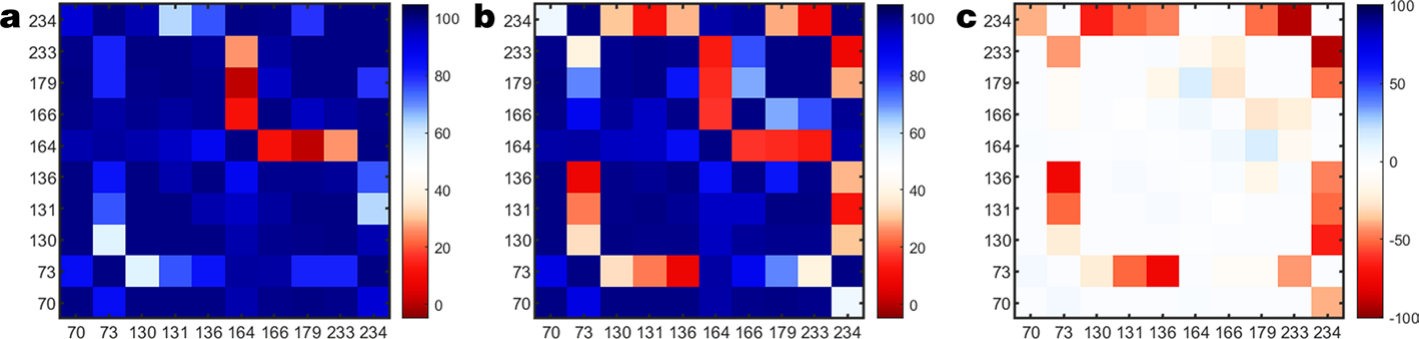
Residue pair interaction scores for the active site residues of: **a** TEM-1 and **b** TEM-52. The interaction score for a residue pair can range from 0-100, where 0 means no interaction and 100 means maximum interaction. **c** shows the difference in the two interaction networks (TEM-52 minus TEM-1) in order to highlight the differences in interaction networks

**Fig. 9:**
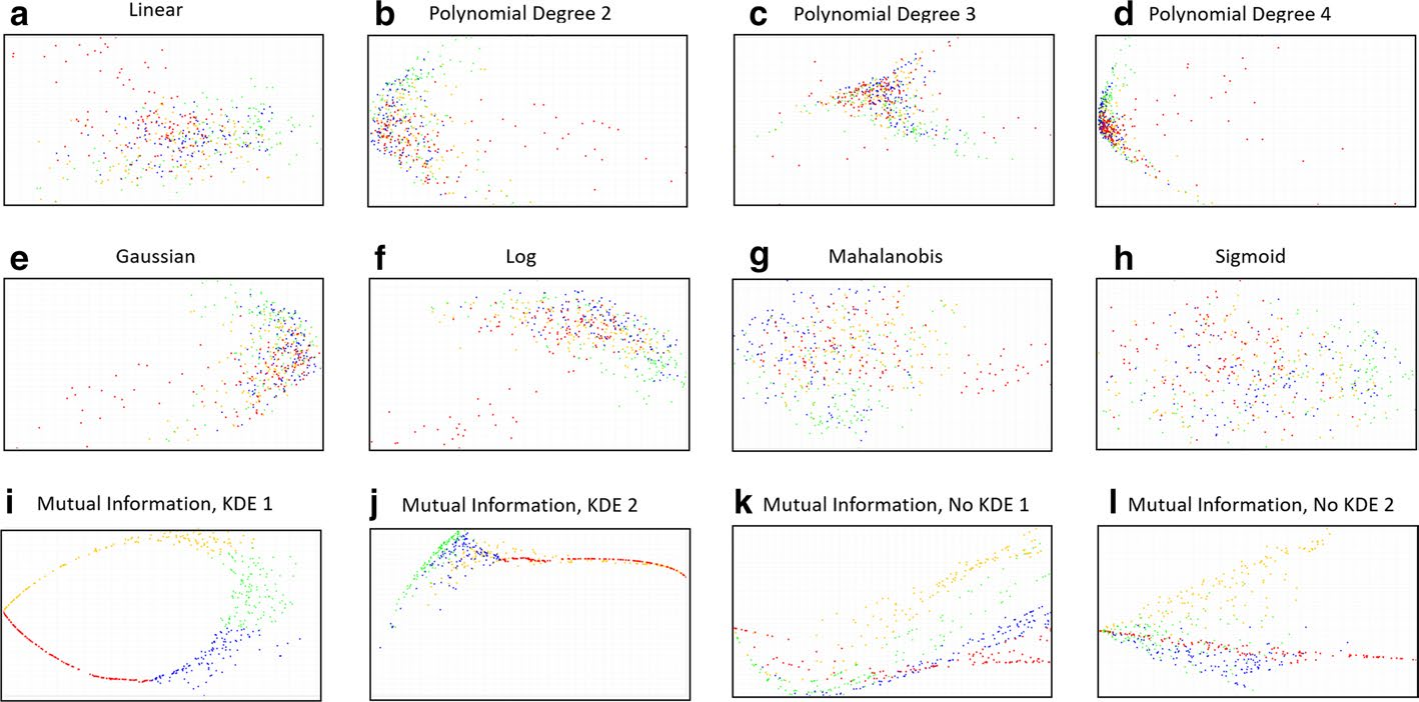
**a**–**i** display some of the kernels computed by JEDi when the KPCA routine is called. In each figure, JEDi colors the conformations by time series, splitting into the trajectory into four segments denoted by red then blue then green then yellow

**Fig. 10:**
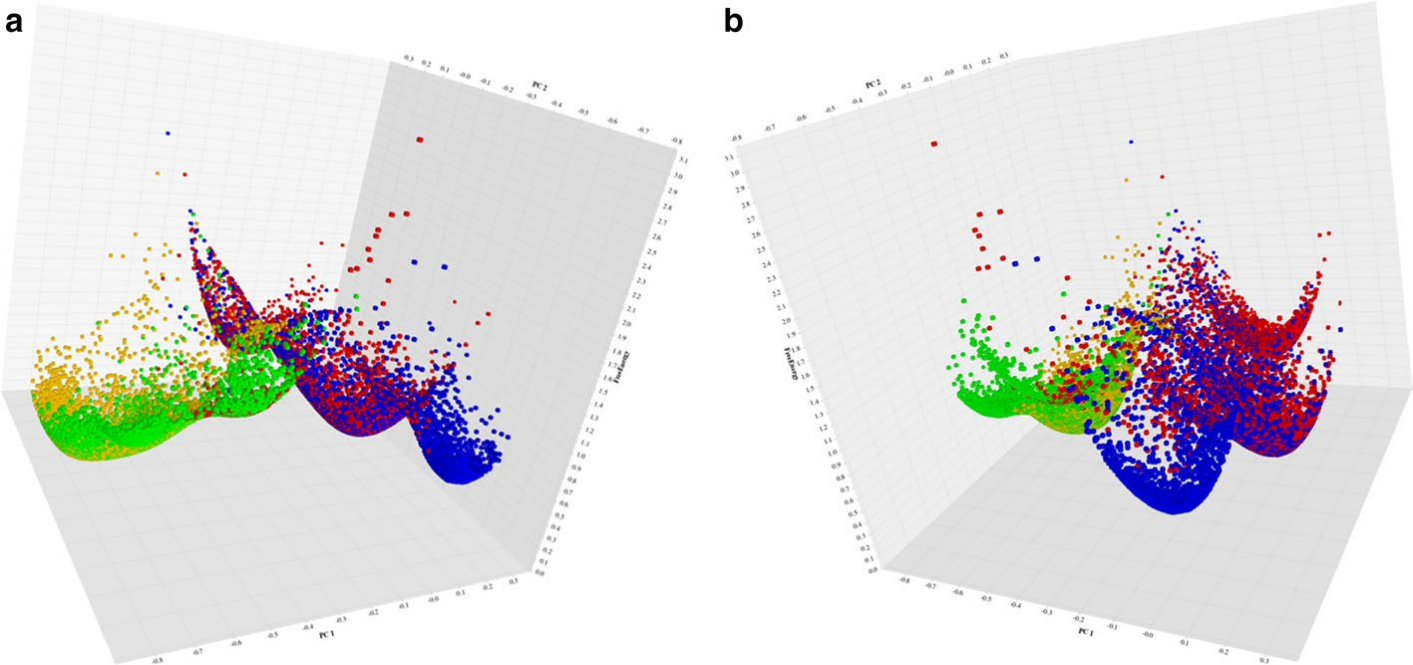
Sample free energy surface output from JEDi. **a** Shows the left side view and **b** shows the right side view

### Residue pair coupling

Calculating residue-residue couplings as a heat map is illustrated in Fig. 8a, b for the active site residues of TEM-1 and TEM-52 respectively. Many of the residues have high interaction scores with each other indicating their pairwise motions are correlated. In Fig. 8c the difference in the interaction matrices for the two enzymes (TEM-52 minus TEM-1) are shown. The resulting change in residue pair couplings is sparse, indicating that most of the residue pair interaction network for both enzymes is similar. However, there is a drop in residue-residue coupling when one of the residues is either LYS73 or LYS234. The drop in residue pair coupling indicates the motions are less coupled in TEM-52. This result is consistent with the TEM-52 binding site being less specific than that of TEM-1.

### Subspace analysis

The subspace analysis function computes RMSIP iteratively as the subspace dimension increases, and estimates the significance of each RMSIP score by comparing to z-scores obtained from multiple random comparisons of an equivalent vector space and subspace dimensions. Additional analysis is run for the entire essential subspace to obtain multiple metrics. When multiple PCA models are being analyzed for the same subset of atoms (including sparsification results), or if explicit PCA and HPCA are selected for the same subset (and resolution), then a subspace analysis will be automatically done for comparison, and the output is directed to labeled sub-directories.

### Visualization of molecular motion

JEDi includes a program which takes eigenvectors from a Cartesian PCA and generates a high quality movie of the dynamics described by each mode and the essential subspace. Details on how the modes are animated are given in the original JED paper [13]. This driver program has been refactored with new capability and now produces updated PyMol scripts for viewing atomistic details. Examples of whole molecule and subset motions are provided in Additional file 1. Several improvements and extensions for visualization has been made that was absent in JED. Examples of these movies can be found in Additional file 2.

### Kernel PCA

JEDi includes a set of programs which take the output of the PCA analyses and pipes them into user selected kernels for KPCA analysis. The PCA preprocessing ensures that the input to the kernels contains the most critical elements of the original data while providing substantial reduction in compute times. JEDi offers more than a dozen different kernels to investigate the presence of non-linear features in the data. The user can turn on this feature and select which kernels to apply. Examples of these kernels are shown in Fig. 9a–l as high quality PNG images for immediate inspection.

### Free energy landscapes

JEDi includes a program which takes the top two PCs or DVPs and computes a free energy surface. The plots are rendered as 3-D scatterplots from two distinct perspectives. This feature can be turned on and will produce graphical output for every PCA analysis. An example of this output can be seen in Fig. 10a, b where high quality PNG images give two distinct perspectives to help identify the geometry of the landscape.

## Conclusions

We developed an essential dynamics analysis toolkit written in Java that performs many tasks that implement best practices for multivariate statistics. The JEDi toolkit offers much more functionality than currently available tools. Analysis methods are integrated, and due to multi-threading, processed largely in parallel. Unique aspects include: subregion and resolution selection, threshold-based processing of rare events into two sets, outliers and inliers, with complete PCA analysis and subspace comparisons; sparsification of R and P matrices with complete PCA analysis, subspace comparisons, and activator and suppressor variable detection; hierarchical PCA using distributed DOF for all atom and heavy atom sets of any size; residue pair interaction analysis; distance pair PCA; atom list PCA; convenient comparative analysis of subspaces using iterated RMSIP scores and principal angles; visualization of essential motions and individual PCA modes; the inclusion of 3 PCA models - covariance, correlation, and partial correlation. A detailed user manual (as a PDF) is made available with the download of the JEDi software package (Additional File 1). The program can be run from compiled source or from executable jar files. Additional resources include example test cases with all JEDi results.

## Availability and system requirements

- **Project name**: Java Essential Dynamics Inspector
- **Project home page**: https://github.com/charlesdavid/JEDi
- **Operating system**: Platform independent
- **Programming language**: Java
- **Other requirements**: JRE version 1.8 or higher
- **License**: GNU GPL 3
- **No restrictions to use**: For reproduction and development, cite the license

## Supplementary information

**Additional file 1.** JEDi User Manual

**Additional file 2.** Supplemental Images: ZIP Archive of MPEG Movies

## Data Availability

Not applicable.

## Abbreviations

PCA: Principal component analysis
JEDi: Java essential dynamics inspector
JED: Java essential dynamics
cPCA: Cartesian principal component analysis
dpPCA: Distance-pair principal component analysis
Q: Covariance matrix
R: Correlation matrix
P: Partial correlation matrix
PDB: Protein data bank
MD: Molecular dynamics
DOF: Degree of freedom
ED: Essential dynamics
PC: Principal component
ICS: Inter-molecular correspondence set
MSA: Measure of sampling adequacy
KMO: Kaiser–Meyer–Olkin
ACS: Adaptive covariance shrinkage
RES: Rare-event shrinkage
MSF: Mean squared fluctuations
DVP: Displacement vector projection
MAD: Median absolute deviation
HPCA: Hierarchical principal component analysis
MCCM: Mode coupling covariance matrix
JRE: Java runtime environment
RMSD: Root mean square deviation
RMSF: Root mean square fluctuation
RMSIP: Root mean square inner product
PA: Principal angle
CO: Cumulative overlap
HPC: High performance computing
KPCA: Kernel principal component analysis
