# Supplementary material for: JEDi: java essential dynamics inspector — a molecular trajectory analysis toolkit
Source: BMC Bioinformatics. 2021 May 1;22:226. doi: 10.1186/s12859-021-04140-5 (PMC8088583; doi:10.1186/s12859-021-04140-5)
Supplement: Supplementary file 1 — Additional file 1. JEDi User Manual [file 12859_2021_4140_MOESM1_ESM.pdf]

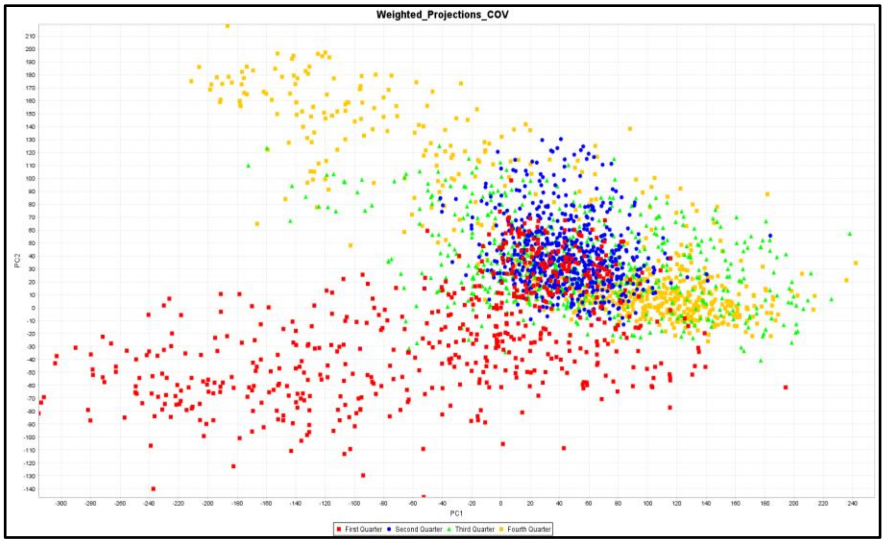

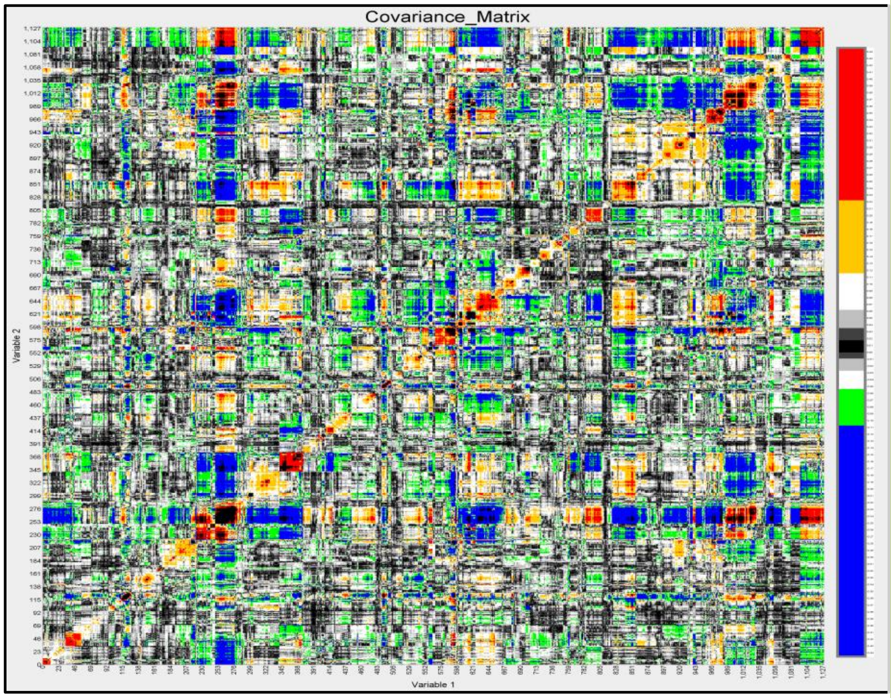

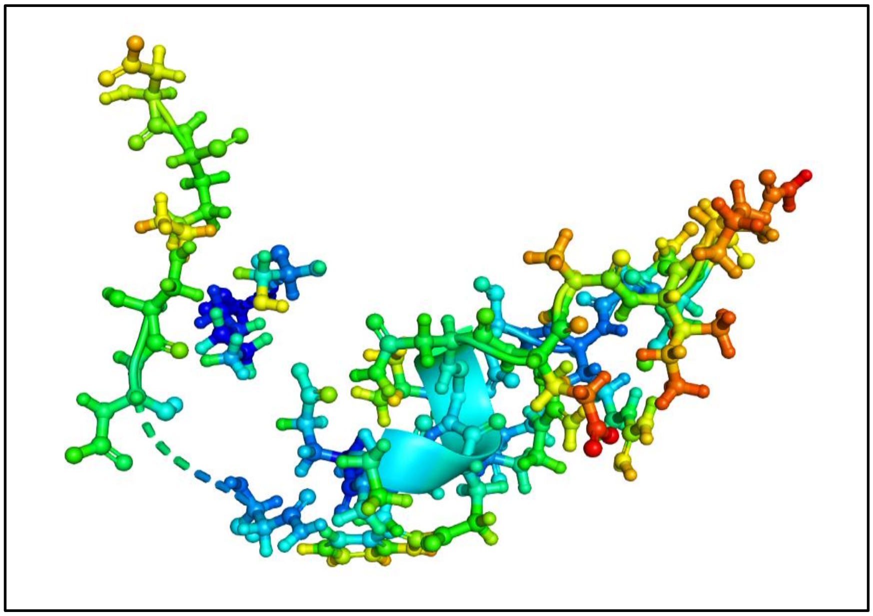

JEDi 2020

Charles David, Donald Jacobs

UNC Charlotte, BMPG

JEDi 2020

Java Essential Dynamics Inspector (JEDi)

User Manual and Tutorial Holocron

Acknowledgement

Dr. Charles David wrote the JED program during his Ph.D. studies in Bioinformatics and Computational Biology at UNC Charlotte under the direction of Dr. Donald Jacobs. Partial support for this work came from NIH grants (GM073082 and HL093531), from the Center of Biomedical Engineering and Science, and the Department of Physics and Optical Science. JEDi is the updated and extended version of JED.

GNU General Public License Agreement

If you choose to use JED or JEDi software, you agree to cite the references listed below on all publications that present results based on the JEDi analysis, and you agree to abide by the GNU General Public License Agreement (version 3).

The GNU General Public License Agreement can be found at http://www.gnu.org/licenses/gpl.html

Citations

Charles C. David, Singam Azimiya, and Donald J. Jacobs. *JED: Java Essential Dynamics*. BMC (publication information).

Charles C. David, Chris Avery, and Donald J. Jacobs. *JEDi: Java Essential Dynamics Inspector*. BMC (publication information).

Supplemental Material: JEDi: Java Essential Dynamics Inspector, C. David, C. Avery, and D. Jacobs

Table of Contents

I. Introduction (Impatient or Hate manuals? Click here!) 6

A. Expected Input to JEDi: Pre-Processing 7

B. JEDi Pre-Processing Output: 8

C. Expected Input to JEDi: Production Run 8

D. Levels of coarse graining: 8

E. Types of PCA: 9

F. PCA Models: 9

G. Outlier Processing & Rare Events: 10

H. Conditioning of the sample Q Matrix: 11

I. Defining Subsets of Residues and Atoms: 12

J. Visualization of PCA modes: 12

K. Dimension Reduction Level: 12

L. Delta Vectors, Displacement Vector Projections, and Principle Components: 13

M. Post PCA Kernel PCA Processing: 13

N. Post PCA Comparative Subspace Analysis: 13

II. Using JEDi 15

A. JEDi Install Instructions: 15

B. Expected Memory Requirements: 15

C. The JEDi Driver: 15

D. Input File for JEDi Driver: 15

III. Running JEDi Driver: 18

A. JEDi Command Line format: 18

B. Organization of Output Files: 18

C. Current Limitations: 19

IV. Getting Started: 20

A. The Pre-Processing Run: 20

B. Common Causes for JEDi to Crash or Exit: 20

V. Running Jobs Using JEDi DRIVER 21

A. The Pre-Processing Run 21

i. Parameters to set in the parameters file: 21

ii. Output Files: 21

B. Performing Production Runs 22

i. Parameters to set in the parameters file depend on what you want to do. 22
ii. Root Output Files: 22
iii. Process Dependent Output Files: 22
iv. Residue List Format 23
v. Atom List Format for Distance Pairs 23
C. Debugging Crashes: Fixing and Preventing Problems: 24
i. Simple mistakes: 24
ii. More subtle issues: 24
iii. Very subtle issues: 25
D. Performing Mode Visualization 26
VI. Additional Types of Analysis 27
A. Pooling Data: 27
i. Run Command: 27
ii. Input File format: 27
iii. Output File format: 28
B. Subspace Analysis: 28
i. Run Commands: 28
ii. Input File format: 28
C. Free Energy Surface: 29
i. Run Commands: 29
ii. Input File format: 29
D. Kernel PCA: 31
i. Run Commands: 31
ii. Input File format: 31
iii. Output 31
E. Essential Mode Visualization: 32
i. Run Commands: 32
ii. Input File format: 32
iii. Output: 33
VII. Appendix 1 - Input File Formats 34
A. JEDi_Parameters.txt 34
B. Residue List file 36
C. Atom Pair List file 36

D. POOL.txt 36

E. SSA.txt 36

F. FES.txt 36

G. KPCA_Parameters.txt 37

H. VIZ.txt 37

VIII. Appendix 2 - Output File Formats: 38

A. Sample JEDi Log file: Abridged (…) for space considerations 38

B. Sample PDB READ Log file: 44

C. Sample SSA Log File: 45

D. Sample FSSA Iterated Log File: 46

E. Sample Plots: 47

F. Sample PCA Projection Plots: 53

G. Sample Kernel PCA Projection Plots: 54

H. Sample FES Plots: 55

I. Sample RMSIP Plots: 57

➢ COV versus CORR Model 57

➢ CORR versus PCORR Model 57

J. Sample atomic RMSF Edited PDB files: 58

IX. Appendix 3 - For the Impatient… 59

A. All you need to do a JEDi Pre-Processing Run is a working directory with the following: 59

B. All you need to do a JEDi Production Run is a working directory with the following: 59

C. The JEDi Pre-Processing Workflow: 60

D. The JEDi Production Workflow: 60

Supplemental Material: JEDi: Java Essential Dynamics Inspector, C. David, C. Avery, and D. Jacobs

I. Introduction (Impatient or Hate manuals? Click here!)

Java Essential Dynamics Inspector (JEDi) is a *multi-threaded* java library (a package of programs) for analyzing protein trajectories. The trajectories may be derived from MD, FIRST/FRODA, or any other dynamic simulations that output a trajectory (equivalent to a set of PDB files). The program can handle single chain PDB files with no chain identifier as well as multi-chain PDB files that use unique chain IDs.

For the initial processing run, the PDB files are read into a matrix of coordinates:

- The input PDB files may be uncompressed, individually compressed, or in a compressed archive.
- Supported formats include `bz2`, `gz`, `zip`, `tar.bz2`, and `tar.gz`.

For production runs, the user specifies:

- The **subset** of residues or atoms to be considered for each analysis (which need not be contiguous)
- The **resolution** for each analysis (e.g., all atom or heavy atom)
- **Statistical thresholds** for identifying atoms/residues with high variance, skew, or kurtosis
- **Outlier Processing:** Inliers versus Outliers using z-score (or MAD) thresholds
- The **statistical models** to use in the PCA
- Thresholds for **sparsifying** the correlation and partial correlation matrices
- Optionally, key parameters (to change default settings)

A variety of utility tools are provided for statistical thresholding of variables and matrices, detecting 'interesting' atom and residue sets, constructing PCA modes and PC projections, performing subspace analysis, constructing visual representations of the individual and essential modes, constructing Free Energy Landscapes using the top two modes as order parameters, and post-processing of the PCA reduced data with Kernel PCA (KPCA).

The outputs include verbose log files, both flat-files and high-res PNG images of all plots, and sets of PDB files with PyMol scripts for reproducing the essential and individual PCA mode motions.

The functionality of JEDi was designed for the specific purpose of examining dynamic trajectories using multiple statistical and visual analytical approaches, with the understanding that resolving the complex underlying dynamics is highly dependent on the tools that one uses to interrogate the system.

JEDi software provides an **integrated suite of tools** that is not found in current MD-simulation packages or other stand-alone PCA software, especially in regards to inspection of the essential dynamics and the comparative analysis of multiple trajectories.

- JEDi is capable of running on any platform with a suitable Java Runtime Environment (JRE).
- JEDi is multi-threaded and implements the Java Executor service with a Cached Thread Pool
    - When running on HPC resources, always request sufficient # of cpus for requested analyses
    - Use the `Number of Analyses + 2` formula for best performance

Supplemental Material: JEDi: Java Essential Dynamics Inspector, C. David, C. Avery, and D. Jacobs

A. Expected Input to JEDi: Pre-Processing

Ideally, each PDB structure must follow standard PDB-format.

Moreover, it is required that every PDB file used in the creation of the coordinates matrix be identical, meaning specifically, that **they must all contain the <u>same atoms</u> and <u>in the same order</u>**.

- When pooling PDBs, as in studies with mutants, it is critical that only the atoms in the intersecting correspondence set (ICS) are used.
- We recommend that the \`doParityCheck\` option is set to true when performing the pre-processing run on pooled data (you need only include one representative of each mutant to verify parity).
    - The parity check verifies atom type, symbol, residue number, and residue type only.
    - In point mutants, atom numbers will vary.
    - For residue INDELs, much more care is needed to select the ICS.

JEDi will process PDB records with header 'ATOM' or 'HETATM' only.

Additionally, JEDi expects that each chain in the PDB file has a unique chain ID.

For the case of a single chain PDB with no chain ID, JEDi will automatically assign a default chain ID of 'A' to the chain.

We highly recommend that users review their PDB files carefully and ensure that they are all-atom and consistent with all formatting guidelines. When generating PDB files from binary trajectory files, ensure that standard PDB output is selected and specify unique chain IDs for all entries.

<u>Note1</u>: It is recommended to label PDB files using **zero padding** (leading zeros) in the name of the files to simplify tracking time progression. For example, if a simulation generates 100,000 frames in the trajectory, it is best to name the PDB files like: <file_name_000001>, <file_name_000002> … <file_name_100000>, which specifies that relative to the starting structure 100,000 frames are generated in successive order. Although this naming scheme is not required, it is highly recommended as it allows the user to track time order easily on operating systems that sort order by literal alphabetic-characters (rather than interpreting 34 is less than 100, for example).

<u>Note2</u>: When preparing a compressed archive, it is recommended to **sort** the files into a file list and then pass that list to the archiver. For example, if using \`tar\`, it is best to gather the files into a single directory and then pass a sorted file list using the \`–T\` option. This is absolutely critical as the \`tar\` program by default will place the files into the archive in a random order, which randomizes the trajectory frames.

<u>Note3</u>: By default, JEDi assumes that pdb files end in the suffix '**.pdb**'.

The supported **individual file formats** include the following <u>only</u>:

- .pdb
- .pdb.bz2
- .pdb.gz
- .pdb.zip

The supported **archive formats** include the following <u>only</u>:

- . tar.bz2
- . tar.gz
- .zip

B. JEDi Pre-Processing Output:

The first step in using JEDi is a pre-processing step in which JEDi either reads:

- All PDB files in a specified directory, or
- All PDB files in a specified compressed archive
  - (Using the 'doRead_Archive=true' and 'Archive_Name=name' parameters)

The **Matrix of the all-atom PDB coordinates,** obtained from all the atoms in the input PDB files is created.

- This file can be used for all subsequent JEDi runs.
- Like most of the flat files output by JEDI, it is compressed using **BZIP2 format**.
  - **Original_PDB_Coordinates_AA.txt.bz2**

A **Residue List File** of all the residues found in the PDB files (along with the chain IDs) is generated.

The aligned **conformation rmsd plot** is determined for each member structure in the trajectory relative to the specified reference structure (ref PDB). JEDi uses a quaternion alignment algorithm.

The Matrix of the **aligned all atom PDB coordinates** is created (Use 'doOutputCoordinates=true' flag).

Statistics for each atom is computed over the entire trajectory: **Mean**, **Variance**, **Skew**, and **Kurtosis**

- These statistics are output as 3-series plots (x ,y, z variables)

Atom (Residue) Lists are generated for every atom (residue) that exceeds a set statistical threshold. For example:

- VARIANCE_THRESHOLD=1.250
- SKEW_THRESHOLD=2.000
- KURTOSIS_THRESHOLD=8.000

The **atom rmsd plot** (also commonly referred to as atomic **RMSF**) is determined from the entire trajectory.

An **edited PDB file** is generated where atomic B-factors are replaced with the **atomic rmsf** values for visualization.

Files listing the **number of atoms in each residue** and the **number of heavy atoms in each residue** are produced.

C. Expected Input to JEDi: Production Run

- JEDi Input file
- PDB Reference file
- Matrix of atomic coordinates in JEDi format
- Atom and Residue list files for the specified subsets

D. Levels of coarse graining:

1. **All-Atom**, includes every atom (and HET atom)
2. **Heavy Atom**, includes all atoms except hydrogen
3. **Back-Bone**, includes the 4 backbone chain atoms (N-C-C-O)
4. **Alpha Carbon**, includes only alpha carbon atoms
5. **Individual Residue**, includes all atoms in the residue

NOTE: **Any subset of residues/atoms that includes HETATM records CAN NOT BE USED FOR BACKBONE OR ALPHA CARBON ANALYSIS** (for obvious reasons…)

E. Types of PCA:

The core element of essential dynamics is to perform PCA. JEDi implements five variations of PCA.

1. **Direct Cartesian** (**cPCA**): The first (and most common) method is based on Cartesian coordinates.
   a. Note that cPCA using ***n* atoms** will yield eigenvectors having ***3n*** components, each corresponding to one Cartesian coordinate.
2. **Hierarchical** (**hPCA**): The second method is done by performing direct Cartesian PCA on each atom (or heavy atom) of every residue in the chosen subset, yielding a set of eigenvectors (Eigen-Residues) and residue principle components (**RPCs**).
   a. A reduced number, ***k*** of those RPCs are selected to represent the residue in a low ***k*** dimensional subspace, where ***k*** measures the informative degrees of freedom (**DOF**).
   b. Those ***k*** RPCs are then used to create a new covariance matrix for the entire subset, where each residue is now represented with ***k*** distributed DOF. The Eigen-Residues are then convoluted with the eigenvectors derived from the factoring of the RPCs.
   c. This method is available for the all-atom and heavy-atom levels, and yields results approximating those from cPCA on those subsets, in much less time.
      i. The closeness of the approximation is offset by performance through the choice of the number of DOFs to use in the hierarchical method.
      ii. We find that using even three DOFs gives much more insight than an alpha carbon analysis, which while it also has 3 DOFs, those DOFs only capture the information of one atom, while in hPCA, the 3DOFs capture information about the entire residue.
3. Distance Pair (**dpPCA**): The third method is based on internal coordinates using atom-pair distances.
   a. The dpPCA method, using ***n* atom-pairs,** will yield eigenvectors having ***n*** components, each corresponding to one of the inter-atom distance pairs.
   b. For small subsets, an all-to-all comparison could be specified.
4. **Residue Pairs Analysis:** The fourth method is done by performing cPCA on every **pair** of residues in the input subset.
   a. Similar to hPCA, a number of distributed DOF are chosen to represent each residue.
   b. Then a Coupling Score is computed that reflects the interaction of the two residues.
5. Atom Set PCA: The fifth method is cPCA applied to a set of atoms.
   a. In this variation, the user specifies a set of atoms as the subset of interest.
   b. The choice of which atoms to include could be informed using some criteria of "interestingness":
      i. Variance
      ii. Skew
      iii. Kurtosis
      iv. Biophysical Insight

F. PCA Models:

All of the PCA methods, except hierarchical, are performed using a **covariance matrix** (**Q**), and optionally, a **correlation matrix** (**R**) and a **partial correlation matrix (P)**. The correlation matrix is a normalized version of the covariance matrix. The results obtained from **Q** and **R** generally differ somewhat due to the inherent statistical biases in each approach. The partial correlation matrix (**P**) is obtained from the inversion of the covariance matrix, with subsequent normalization.

There are noticeable differences in PCA results based on the choice of model. **Q** tends to reveal the largest displacements while obscuring smaller correlated motions. **R** tends to reveal many more atoms participating in any given mode (higher collectivity), thereby emphasizing smaller displacements. **P** tends to be like R but with much less information being captured in each mode. While **R** eigenvalues can be large, **P** eigenvalues do not exceed 2. Considering that in statistics one uses the partial correlation to determine the correlation between two variables after the effects of all other variables have been removed, each **P** mode should contain the information of at most 2 variables, which is the reason for the eigenvalue cutoff of 2. Additionally, the comparison of the **R** and **P** matrices can help identify the '***suppressor'*** and '***activator'*** variables in the subset.

As an option, one may choose to sparsify the **R** and/or **P** matrices using a specified threshold. Whether or not to sparsify is controlled by the flag `**doSPARSIFY**` in the input parameters file.

While JEDi offers the option to threshold **Q**, we do not recommend this procedure.
Instead, we suggest only thresholding the **R** and **P** matrices as these are normalized matrices and Q is not.

The current release of JEDi implements all three PCA models, and compares the results (when selected).

G. Outlier Processing & Rare Events:

To help assess the degree of sampling of the variables (e.g., the (x,y,z) coordinates or distances) in the input data, JEDi computes the **Measure of Sampling Adequacy** (MSA) scores for each variable and the overall **Kieser-Meyer-Olkin** (KMO) score. However, when reviewing trajectory data, it is hard to know if events that have low statistical sampling are true outliers or represent very important rare events. For this reason, JEDi offers the option to both REMOVE and SELECT outliers (aka rare events) based on a specified threshold, and then quantitatively compare the two analyses.

- To activate this feature, set ` **doOutlierProcessing =true**
- Also, set `**Z_SCORE_CUTOFF=x**` or ` **MAD_SCORE_CUTOFF=x** ` with **x** $\in$ **[0.675, 1.960]**

Because PCA is highly sensitive to outliers, it is often a recommended step to examine the data for possible outliers. The simplest method for removing outliers is to calculate the z-scores for each instance of a variable, and then set any instance that is greater than a specified threshold to the mean value for that variable. For variable distributions that deviate greatly from a normal distribution, the method of mean absolute deviations (MAD) can be used, replacing the extreme value with the median. In contrast to outlier removal, one may want to emphasize the impact of rare events from a molecular simulation. In this case, it is necessary to both select the extreme values, and down-weight the effect of the more central values.

In practice, we find that there exists a range of cutoffs that allow good insight into the structure of the data. The exact values for useful cutoffs are somewhat dependent on the size of the subset and the number of samples, but a practical z-score range for most datasets would be **[0.675, 1.96]**. In particular, we note that using a z-score-cutoff of 0.675 allows for a comparison of approximately 50% inliers to 50% outliers, while the choice of 1.96 provides a comparison of approximately 95% inliers to 5% outliers. More extreme values of these cutoffs are possible but come with the risk that little or no sampling will be available for analysis, leading to no useful results and possible crashing of the program.

As a 'Best Practice', we recommend that for any given subset and resolution, the user perform the analysis using no outlier processing, and then an outlier processing process using a few different cutoffs. Good insight can be obtained by comparing the results of those approaches. Our experience shows that when comparing analyses using strong outlier removal (z >= 3.0) with no outlier processing, the essential subspaces as measured with RMSIP are very similar (RMSIP > 0.95). This is due to the fact that JEDi implements covariance shrinkage, thus greatly reducing the impact of outliers on covariance estimation. However, we have seen extremely different essential subspaces emerge when selecting versus removing outliers, even with cutoffs as moderate as z = 0.675. Depending on the situation, one may wish to examine the outputs for both the inliers and outliers to truly understand the essential dynamics of the system.

JEDi handles the removal [selection] of outliers [events] prior to the PCA analyses. The user can specify a cutoff, **z-score (mad-score)** (a decimal ≥ 0) such that when the value of a variable has a |deviation| from the variable mean (median) that is greater [is lower] than the score cutoff, it is identified as an outlier [event]. For each entry that is identified as an

outlier or event, it is replaced with its mean (or median). This process is done per variable, over all frames, and each entry is treated independently. In this process, a frame is never thrown away, but some entries within a frame may be modified.

When JEDi performs outlier processing, an automatic comparison is done between the two derived essential subspaces, using the COV model, to provide a quantitative measure of the differences in the essential motions of the two sets.

Note1: Outlier Removal and Selection are both done and the resulting subspaces compared.
Note2: Z-Score and MAD-Score cutoffs are mutually exclusive.

H. Conditioning of the sample Q Matrix:

Critically, it is well known that sample covariance matrices are poor estimators of the true covariance and the use of such matrices is not recommended. For this reason, JEDi implements a shrinkage algorithm for improving the covariance estimators. The target matrix is "*Diagonal-Unequal-Variances*". The algorithm determines the optimal shrinkage intensity based on the input data and the variance of the entries of the sample covariance matrix. The combination of outlier removal and covariance shrinkage yields statistical estimators that are much more robust than one would get from a naïve approach, and whose spectral decompositions provide reliable insights into the essential subspaces.

Since spectral decomposition of the Q, R, and P matrices is a critical element in JEDi processing, many procedures are done to ensure that they are robust. While outlier removal and shrinkage ensure good statistical estimators of covariance and invertible Q matrices, it is still important to assess the physical meaning of the eigenvalues of any such factorization. A first consideration is the smallest eigenvalue that retains physical relevance. This can be assessed by determining the last significant digit in the analysis. Since the data is obtained from PDB files that only record position information to 3 decimal places, any values less that one thousandth of an angstrom is in the noise and not significant. To accommodate this physical reality, JEDi implements a noise threshold for determining the smallest eigenvalues with physical meaning that by default is set to 1.000E-6 angstrom-squared, as the square root of this variance is associated with the smallest significant displacement (1.000E-3 angstroms).

This threshold is critical to forming the **P** matrix, which relies on the inversion of the **Q** matrix.

The equation for the eigenvalue decomposition of the covariance matrix is: $\mathbf{Q = UDU^{-1}}$.

- The inverse of the covariance matrix is then given by: $\mathbf{Q^{-1} = UD^{-1}U^{-1}}$
  - Where **U** is the matrix of eigenvectors and **D** is the diagonal matrix of eigenvalues.

By controlling the size of the smallest eigenvalue, the precision matrix ($\mathbf{Q^{-1}}$) is always available and well-formed.

- To control this option, set `NOISE_LEVEL=1.000E-6` (for example)

Supplemental Material: JEDi: Java Essential Dynamics Inspector, C. David, C. Avery, and D. Jacobs

I. Defining Subsets of Residues and Atoms:

The user can submit distinct residue lists for the following levels and types of analysis:

- All-Atom
- All-Atom Hierarchical
- Heavy-Atom
- Heavy-Atom Hierarchical
- Backbone Atom
- Alpha Carbon
- Atom List
- Distance Pair
- Individual Residue
- Residue Pairs

The format of the residue lists files is described here.

NOTE: Each of these analyses consumes one thread.

- When performing **all ten analyses**, we recommend that the number of cpus requested be **10 + 2 = 12**.
- This **N + 2** formula for requesting cpus is geared so that java has dedicated threads for house-keeping tasks.

J. Visualization of PCA modes:

JEDi computes the **PCA modes** (RMSD and MSD, with and without weight by the corresponding eigenvalue) from the Cartesian/Displacement/Hierarchical eigenvectors so that they may be mapped to the subset of atoms. Sets of structures (PDB files) can be generated to visually inspect the cPCA and hPCA eigenvectors and modes. Eigenvectors from dpPCA cannot be mapped to the residue set in any simple way, so no mapping or visualization is attempted. The user can specify the number of modes to visualize, beginning with mode one. Mode visualization is done by creating a set of PDB files that capture the displacement of each atom, for each requested mode, using a sine function to perturb the associated PDB coordinate with the associated eigenvector component. A scale-factor parameter is used to control the amount of displacement in the modes. A PyMol™ script is generated to animate the frames. JEDi also produces a set of frames to capture the Essential Modes, using a superposition of the top selected modes. This set of frames captures multiple periods of the fundamental mode. The Input parameters file has a variety of settings to control the number of modes combined for the essential visualization, the number of frames and the number of periods:

K. Dimension Reduction Level:

The primary purpose of applying PCA to capture the essential dynamics of a protein is to reduce the large dimension of variables to a much smaller number of variables that captures the greatest variance in protein motion. The **Q, R,** and **P** matrices, once diagonalized, provide a set of eigenvalues and eigenvectors. The eigenvalues for proteins typically fall off fast for the first several Q and R modes, out of possibly thousands of modes. While the **P** eigenvalues do not fall off rapidly, the primary usefulness of the **P** modes is in the identification of ***activator*** and ***suppressor*** variables. The number of dimensions needed to provide a fair assessment of the essential dynamics in a protein is system-dependent. The user can specify any number (say 20, which typically is more than needed) to obtain results for all possible selections, ranging from 1 up to the maximum value that is selected. In this way, the user can see how the added dimensions help glean more information, albeit making it harder to interpret the greater number of dimensions. The essential subspace can often be accurately identified using the Cattell Criterion for eigenvalues from **Q** or **R**. Eventually, the user must decide, based on their purpose/goals, the optimal number of dimensions to use for representing the essential dynamics.

L. Delta Vectors, Displacement Vector Projections, and Principle Components:

A set of **displacement vectors** (**DV**s) is calculated using a specified reference structure. Those **DV**s are then projected onto a set of eigenvector directions to create delta vector projections (**DVP**s), which are similar to principle components (**PC**s). The **PC**s are delta vector projections, but according to the standard definition used in statistics, they are always relative to the mean conformation position as defined in the construction of the **Q** matrix. In studying the essential dynamics of a protein, it is common to use a reference structure that has a particular physical or biochemical meaning, which is why we call these displacements **DVP**s, and not **PC**s. The DVPs are very useful to have for visualizing protein motions. For example, if the first two eigenvector directions are selected (those eigenvectors associated with the highest and second highest eigenvalues, or variance) the **DVP**s can be plotted for each frame to construct the trajectory in conformational space projected onto a two dimensional cross-section. Other eigenvector directions can be specified, allowing the user to investigate how the trajectory projects into the space defined by each eigenvector. The **DVP**s are given using un-normalized and normalized inner products, as well as weighted by the square root of the corresponding eigenvalue (giving units of angstroms or angstrom squared). The different methods highlight the structure of the data and provide scaling for visualization.

JEDi will automatically plot the top two PCs or DVPs, producing a scatter plot with resolution 1600x900 in PNG format. The plots are divided into quarters of the trajectory, and colored accordingly. This allows for immediate review of the data in each analysis as a function of time/place in the trajectory.

M. Post PCA Kernel PCA Processing:

JEDi offers a Boolean option of performing kernel PCA (**`doKPCA=true`**) on the reduced data from the PCA analyses done. If selected, JEDi will perform KPCA, using multiple kernels, on the top selected PCs or DVPs. The number ***x*** of PCs to use is set with the (**`NUMBER_PCs_INPUT=x`**) parameter. The user can set any or all of the kernels to true in the input parameters file to activate their use in this analysis.

A separate KPCA driver is available for fine tuning the KPCA analysis, but we find that even using the top two PCs allows quick calculation times and deep insight into any nonlinear structure of the data. The current kernels include Linear, Mutual Information (3 versions, one based on 2D KDE), Cauchy, Circular, Gaussian (radial basis function), Sigmoid (hyperbolic tangent function), Log, Euclidean (distance and similarity), Mahalanobis (distance and similarity), and Polynomial (homogeneous, degrees 2, 3, and 4).

Just as with the standard PCA PC plots, JEDi will automatically plot the top two Kernel PCs, producing a scatter plot with resolution 1600x900 in PNG format. The plots are divided into quarters of the trajectory, and colored accordingly. This allows for immediate review of the data in each analysis as a function of time/place in the trajectory.

N. Post PCA Comparative Subspace Analysis:

When multiple models are selected, JEDi performs inter-model subspace analysis (**SSA**) on the two equidimensional sets of eigenvectors generated from the **Q, R,** and **P** variants of PCA (and sparse variants when selected). The results provide a detailed comparison for the chosen essential subspace, as well as comparisons for all subspace dimensions up to the dimension chosen by the user (when selecting the number of Cartesian/Displacement/Hierarchical or Distance modes to process) in an iterative fashion. This allows one to quantitatively determine how different the PCA results are due only to the choice of PCA model, while also assessing the size of the essential subspace.

Moreover, JEDi performs an inter-type subspace analysis when results from identical subsets are obtained using different types of PCA (the vector spaces and the subspaces must have the same dimensions).

To provide a base-line for the reported RMSIP values, JEDi computes a RMSIP score for **two random subspaces** of the representative vector space, iterated, so that one can assess the effect of choice of subspace dimension on the results.

When comparing essential subspaces, keep in mind that the RMSIP metric depends on both the dimension of the SS and the dimension of the full VS. One way to assess PCA modes is to compare them to the modes of a random process to obtain a baseline for determining the significance of the subspace comparisons as the dimensions for the subspace and full vector space change. With these baselines, a Z-score can be calculated to assess the statistical significance of the scores.

Output of the subspace analysis includes a log file and a 3-series plot of the (1) iterated RMSIP score, (2) the random RMSIP score with error bars, and (3) Z-score for accessing significance.

Additional analysis can be done using the driver programs for the Subspace Analysis class. To perform these kinds of tests, it is a best-practice to first generate equidimensional sets of eigenvectors from each trajectory of interest, as well as from a pooled trajectory to use as a reference set, while ensuring that the subsets of residues analyzed are identical. Subspace analysis is done by comparing the sets of eigenvectors, directly or iteratively, and determining the root mean square inner products (**RMSIP**s), Principal Angles (**PA**s), cumulative overlap (**CO**s), cosine products (**CP**s), vectorial angular sum (**VAS**), and the maximum angle between subspaces of the given vector space. JEDi produces summary log files for both of these analyses.

Supplemental Material: JEDi: Java Essential Dynamics Inspector, C. David, C. Avery, and D. Jacobs

`(In this manual, code, file paths, and text file content are shown in dark blue Consolas)`

II. Using JEDi

A. JEDi Install Instructions:

Java is **platform independent** and JREs exist for all common architectures. The machine on which JEDi is to be run should have **JRE version 1.8 or higher** installed. The programs can be run from compiled source or from the provided executable jar files. While JEDi can be installed in any directory that is part of your Java classpath, the sources must be compiled on the local machine to insure runtime integrity.

- Dependencies: (these are in the **`/library`** folder)
    - JAMA Matrix: `Jama-1.0.3.jar`
    - Java Commons: `jcommon-1.0.23.jar`
    - Apache Commons Compress: `commons-compress-1.19.jar`
    - JFreeChart: `jfreechart-1.0.19.jar`, `jfreechart-1.0.19-experimental.jar`, `jfreechart-1.0.19-swt.jar`
    - PDF Estimator: `estimatePDF.jar`

When compiling from source, be sure to compile the **Jama, Java Commons**, **Apache Commons Compress**, **JFreeChart, and PDFEstimator** source packages as JEDi depends on those libraries, or ensure that the package JAR files are on the Java classpath.

Alternatively, no source code or compilation is needed to run the executable jar files. These contain all source code and dependencies and can be placed in any directory that is on the Java classpath. For most applications, a **64bit OS is required** to address the amount of memory needed for the analyses. It is critical that the environment variable Java **CLASSPATH** be correctly set to run Java programs at the command prompt. Alternatively, you can always add the **-cp** option to the **java** command, which allows you to specify the path that contains your Java classes.

B. Expected Memory Requirements:

On high performance computer clusters make sure the 64 bit JRE is installed. Memory use is demanding because JEDi loads the complete covariance matrix (among other data structures) and performs a full matrix diagonalization, which scales as $O(N^3)$. Typically 8 to 32 GB of RAM will be needed depending on the size of the protein. For very large proteins consisting of tens of thousands of atoms and/or many tens of thousands of frames, select a high memory node and request as much RAM as possible. On most platforms, Java can be optimized by specifying parameters at runtime for heap space, etc.

C. The JEDi Driver:

The JEDi programs are run by a single class, JEDi_Driver.java. This class contains the main method and instantiates all analysis. The key to running JEDi is in specifying the parameters for the analyses.
This is made simple by having the driver class read in an input file that contains all needed information.

D. Input File for JEDi Driver:

JEDi requires an input file that specifies job parameters. The format of this file is a simple list of **KEY=VALUE** pairs. There must be no spaces in the declaration. Lines starting with a "#" are ignored. The run command takes only one (optional) argument, which is the name of the input file that includes the absolute path to the file. If no argument is specified, then JEDi assumes that the default input file name is used and that the file is located in the same directory from which the Java Virtual Machine (JVM) was called.
The default input file name is: **`JEDi_Parameters.txt`**

Below is an **annotated** version of the input file: (*cannot be used in this form for running jobs!*)

```
# ---------------------------------------------------------------------------------------------------------------------------------------
#     DIRECTORY, DESCRIPTION, REFERENCE_PDB
# ------------------------------------------------------------
DIRECTORY=/working/directory/                  ----->     Working directory (must end with a `File.separator.char` ('\' or '/')
DESCRIPTION=TEST                               ----->     Job Description (no spaces)
REFERENCE_PDB=pdb_file.pdb                     ----->     Name of the PDB reference file (must be in working directory)
# ---------------------------------------------------------------------------------------------------------------------------------------
#     PRE_PROCESSING
# ---------------------------------------------------------------------------------------------------------------------------------------
doPREPROCESS=false                            ----->     Set to true when doing a Pre-Processing Run ONLY
doParityCheck=false                           ----->     Set to true to verify that all PDB files are equivalent
# ---------------------------------------------------------------------------------------------------------------------------------------
doREAD_ARCHIVE=false                          ----->     Set to true when reading files from a compressed archive ONLY
ARCHIVE_NAME=pdb_archive.zip                  ----->     Name of archive file
# ---------------------------------------------------------------------------------------------------------------------------------------
#     STATISTICAL THRESHOLDING
# ---------------------------------------------------------------------------------------------------------------------------------------
VARIANCE_THRESHOLD=1.000                      ----->     Variance Threshold for atom/residue list selection
SKEW_THRESHOLD=2.000                          ----->     Skew Threshold for atom/residue list selection
KURTOSIS_THRESHOLD=8.000                      ----->     Kurtosis Threshold for atom/residue list selection
# ---------------------------------------------------------------------------------------------------------------------------------------
#     COORDINATES FILE
# ---------------------------------------------------------------------------------------------------------------------------------------
ORIGINAL_PDB_COORDS=original_PDB_Coordinates_AA.txt    ----->     All-Atom PDB Coordinates File (from the Pre-Processing step)
# ---------------------------------------------------------------------------------------------------------------------------------------
#     PCA SUBSETS & RESOLUTIONS
# ---------------------------------------------------------------------------------------------------------------------------------------
doAA=true                                     ----->     Set to true to select All Atom Subset Analysis
doHA=true                                     ----->     Set to true to select Heavy Atom Subset Analysis
doBB=false                                    ----->     Set to true to select Back-Bone Atom Subset Analysis (NO HETATMs)
doCA=false                                    ----->     Set to true to select Alpha Carbon Atom Subset Analysis (NO HETATMs)
# ---------------------------------------------------------------------------------------------------------------------------------------
doRESIDUE_INDIVIDUAL=true                     ----->     Set to true to perform the all atom Individual Residue Analysis
doRESIDUE_PAIRS=false                         ----->     Set to true to perform the Residue Pairs Analysis
# ---------------------------------------------------------------------------------------------------------------------------------------
doHIERARCHICAL_AA=true                        ----->     Set to true to perform the Hierarchical All Atom Analysis
doHIERARCHICAL_HA=true                        ----->     Set to true to perform the Hierarchical Heavy Atom Analysis
# ---------------------------------------------------------------------------------------------------------------------------------------
doATOM_LIST=true                              ----->     Set to true to perform the Atom List Analysis
doATOM_PAIRS=false                            ----->     Set to true to perform the Distance Pair Analysis
# ---------------------------------------------------------------------------------------------------------------------------------------
#     PCA STATISTICAL MODELS
# ---------------------------------------------------------------------------------------------------------------------------------------
doCORR=true                                   ----->     Set to true to perform the Correlation PCA models
doPCORR=true                                  ----->     Set to true to perform the Partial Correlation PCA models
# ---------------------------------------------------------------------------------------------------------------------------------------
#     NUMBER of PCA MODES
# ---------------------------------------------------------------------------------------------------------------------------------------
MODES_RESIDUE_PAIRS=1                         ----->     Number of distributed DOFs for the Residue Pairs analysis (Eigen Residues)
MODES_RESIDUE_INDIVIDUAL=20                   ----->     Number of PC modes for the Individual Residue analysis
# ---------------------------------------------------------------------------------------------------------------------------------------
MODES_RESIDUE_AA=3                            ----->     Number of distributed DOFs for the Hierarchical All-Atom analysis (Eigen Residues)
MODES_HIERARCHICAL_AA=20                      ----->     Number of PC modes for the Hierarchical All-Atom analysis
# ---------------------------------------------------------------------------------------------------------------------------------------
MODES_RESIDUE_HA=3                            ----->     Number of distributed DOFs for the Hierarchical Heavy-Atom analysis (Eigen Residues)
MODES_HIERARCHICAL_HA=20                      ----->     Number of PC modes for the Hierarchical Heavy-Atom analysis
# ---------------------------------------------------------------------------------------------------------------------------------------
MODES_ALL_ATOM=15                             ----->     Number of PC modes for the All-Atom analysis
MODES_HEAVY_ATOM=15                           ----->     Number of PC modes for the Heavy-Atom analysis
MODES_BACKBONE=15                             ----->     Number of PC modes for the Back Bone analysis
MODES_ALPHA_CARBON=15                         ----->     Number of PC modes for the Alpha Carbon analysis
# ---------------------------------------------------------------------------------------------------------------------------------------
MODES_ATOMS_LIST=21                           ----->     Number of PC modes for the Atom List analysis
MODES_DISTANCE_PAIRS=21                       ----->     Number of PC modes for the Distance Pair analysis
# ---------------------------------------------------------------------------------------------------------------------------------------
#     RESIDUE & ATOM SUBSETS
# ---------------------------------------------------------------------------------------------------------------------------------------
RESIDUE_LIST_ALL_ATOM=residues.txt            ----->     Residue list for the All-Atom analysis
RESIDUE_LIST_HEAVY_ATOM=residues.txt          ----->     Residue list for the Heavy-Atom analysis
RESIDUE_LIST_BACKBONE=residues.txt            ----->     Residue list for the Backbone-Atom analysis
RESIDUE_LIST_ALPHA_CARBON=residues.txt        ----->     Residue list for the Alpha Carbon-Atom analysis
# ---------------------------------------------------------------------------------------------------------------------------------------
RESIDUE_LIST_INDIVIDUAL=residues.txt          ----->     Residue list for the Individual Residue analysis
RESIDUE_LIST_PAIRS=residues.txt               ----->     Residue list for the Residue Pairs analysis
# ---------------------------------------------------------------------------------------------------------------------------------------
RESIDUE_LIST_HIERARCHICAL_AA=residues3.txt    ----->     Residue list for the Hierarchical All-Atom analysis
RESIDUE_LIST_HIERARCHICAL_HA=residues3.txt    ----->     Residue list for the Hierarchical Heavy-Atom analysis
# ---------------------------------------------------------------------------------------------------------------------------------------
ATOM_PAIRS_LIST=atom_pairs.txt                ----->     Residue list for the Distance Pair analysis
ATOMS_LIST=atom_list.txt                      ----->     Residue list for the Atom List analysis
# ---------------------------------------------------------------------------------------------------------------------------------------
#     DOWN-SAMPLING
# ---------------------------------------------------------------------------------------------------------------------------------------
doDownSample=true                             ----->     Set to true to down sample the number of frames
DOWNSAMPLE=10                                 ----->     Number of reduced frames = number_of_frames/DOWNSAMPLE
# ---------------------------------------------------------------------------------------------------------------------------------------
doFrameSelect=false                           ----->     Set to true to select a range of frames
FRAME_START=1                                 ----->     First frame to select
FRAME_END=1000                                ----->     Last frame to select
# ---------------------------------------------------------------------------------------------------------------------------------------
#     OUTLIER PROCESSING
# ---------------------------------------------------------------------------------------------------------------------------------------
doOutlierProcessing=true                      ----->     Set to true to process outliers/rare events
MAD_SCORE_CUTOFF=0                            ----->     MAD-Score Cutoff for outlier detection/selection
Z_SCORE_CUTOFF=0                              ----->     Z-Score Cutoff for outlier detection/selection
# ---------------------------------------------------------------------------------------------------------------------------------------
```

```
#     SPARSIFICATION
# ------------------------------------------------------------------------------------------------------------------------------------------
doSPARSIFY=false                            ----->     Set to true to SPARSIFY Q, R, and/or P matrices
# ------------------------------------------------------------------------------------------------------------------------------------------
THRESHOLD_COV=0                             ----->     Threshold for entries in covariance matrix
THRESHOLD_CORR=0.500                        ----->     Threshold for entries in correlation matrix
THRESHOLD_PCORR=0.100                       ----->     Threshold for entries in partial correlation matrix
# ------------------------------------------------------------------------------------------------------------------------------------------
#     REDUCE DYNAMIC MATRICES (3N --> N)
# ------------------------------------------------------------------------------------------------------------------------------------------
doREDUCE=true                               ----->     Set to true to REDUCE dynamic matrices
# ------------------------------------------------------------------------------------------------------------------------------------------
#     FREE ENERGY LANDSCAPE
# ------------------------------------------------------------------------------------------------------------------------------------------
doFES=true                                  ----->     Set to true to perform construction of Free Energy Surfaces
# ------------------------------------------------------------------------------------------------------------------------------------------
#     KERNEL PCA
# ------------------------------------------------------------------------------------------------------------------------------------------
doKPCA=true                                 ----->     Set to true to perform the Kernel PCA on PCA reduced data
# ------------------------------------------------------------------------------------------------------------------------------------------
MAX_KERNEL_FRAMES=500                       ----->     Threshold for entries in kernel matrices
NUMBER_PCs_INPUT=5                          ----->     Number of PCs to use as input to the kernel matrices
KERNEL_SHRINKAGE=0.010                      ----->     shrinkage for kernel matrices
KPCA_SIGMA=0                                ----->     sigma for Gaussian, Cauchy, and Circular kernel matrices
KPCA_SLOPE=0                                ----->     slope for sigmoid kernel matrices
MULTIPLIER=100                              ----->     multiplier for sigma and slope for entries in kernel matrices
# ------------------------------------------------------------------------------------------------------------------------------------------
Linear=false                                ----->     Set to true to do Linear kernel
Degree_2_Poly=true                          ----->     Set to true to do homogeneous Degree 2 polynomial kernel
Degree_3_Poly=true                          ----->     Set to true to do homogeneous Degree 3 polynomial kernel
Degree_4_Poly=true                          ----->     Set to true to do homogeneous Degree 4 polynomial kernel
XY_Poly=false                               ----->     Set to true to do homogeneous Degree 2 polynomial cross kernel
Euclidean=true                              ----->     Set to true to do Euclidean Distance kernel
Mahalanobis=true                            ----->     Set to true to do Mahalanobis Distance kernel
Gaussian=true                               ----->     Set to true to do Gaussian kernel
Sigmoid=true                                ----->     Set to true to do Sigmoid kernel
Log=true                                    ----->     Set to true to do Log kernel
Circular=false                              ----->     Set to true to do Circular kernel
Cauchy=false                                ----->     Set to true to do Cauchy kernel
MI=true                                     ----->     Set to true to do Mutual Information kernel
MI_KDE=true                                 ----->     Set to true to do Mutual Information with KDE kernel
# ------------------------------------------------------------------------------------------------------------------------------------------
#     MODE VISUALIZTION
# ------------------------------------------------------------------------------------------------------------------------------------------
doModeViz=true                              ----->     Set to true to output files for Visualizing Individual PCA Modes
doEssentialViz=true                         ----->     Set to true to output files for Visualizing The Essential Subspace
MODES_VIZ=10                                ----->     The number of modes to visualize
# ------------------------------------------------------------------------------------------------------------------------------------------
#     ADVANCED PARAMETERS
# ------------------------------------------------------------------------------------------------------------------------------------------
READ_PDBS_FILTER_STRING=.pdb                ----->     String for choosing PDB files: Default = ‘.pdb’ → “return(name.contains(READ_PDBS_FILTER_STRING)”
# ------------------------------------------------------------------------------------------------------------------------------------------
FLOOR=1.000E-16                             ----->     Numerical FLOOR = smallest value allowed
NOISE_LEVEL=1.000E-6                        ----->     Noise level = square of smallest meaningful distance measure
KDE_RESOLUTION=20                           ----->     parameter for setting KDE resolution
KDE_CELL=64                                 ----->     parameter for setting KDE cell size
KDE_MARGIN=0.250                            ----->     parameter for setting KDE table margin
# ------------------------------------------------------------------------------------------------------------------------------------------
VIZ_MODE_SCALE_FACTOR=0.250                 ----->     Amplification Factor for mode perturbations (default = 0.250)
LOG_FLOOR=1.000E-4                          ----->     NUMERICAL FLOOR for log coloring scheme for PDB files
numberModeCycles=1                          ----->     Number of cycles to visualize for the individual modes
numberModeFrames=10                         ----->     Number of frames to output for the individual modes
numberModeComponents=10                     ----->     Number of individual modes to combine in forming the essential mode visualization
numberEssentialCycles=3                     ----->     Number of cycles to visualize for the essential motion
numberEssentialFrames=30                    ----->     Number of frames to output for the essential motion
# ------------------------------------------------------------------------------------------------------------------------------------------
#     OUTPUT CONTROL
# ------------------------------------------------------------------------------------------------------------------------------------------
verbose=true                                ----->     Set to true to output all text (flat) files from the analyses
doOutputCoordinates=false                   ----->     Set to true to output the subset aligned PDB coordinates as compressed (bzip2) matrices
# ------------------------------------------------------------------------------------------------------------------------------------------
```

III. Running JEDi Driver:

Each job should be assigned to its own working directory.

- Pre-Processing Runs:

- **JEDi_Parameters.txt** (input file)
- The **PDB files** (or archive) to read
- The **reference PDB file**

- Production Runs:
  - **JEDi_Parameters.txt** (input file)
  - The **Coordinates Matrix**
  - The matching **reference PDB file**
  - The **residue list(s)** for specifying the subset(s) of interest.

A. JEDi Command Line format:

To run **JEDi_Driver** at the command prompt or within a PBS script, you can use one of the following commands:

```
java -d64 JEDi_Driver /path/to/your/input/file.txt (runs the compiled java program)
java -jar -d64 JEDi_Driver.jar /path/to/your/input/file.txt (runs the executable jar file)
```

Remember to include command line switches to optimize the Java runtime environment for your jobs:

- `-d64, -server, -Xms, -Xmx, -XX:MaxGCPauseMillis, -XX:+UseLargePages, -XX:+AlwaysPreTouch, -XX:+DisableExplicitGC`
  - Set: Xms = Xmx, -XX:+AlwaysPreTouch, -XX:MaxGCPauseMillis=10000, and -XX:+DisableExplicitGC to eliminate heap resizing and back virtual memory with physical memory, turn off explicit GC, and increase GC pauses.

For example:

```
java -d64 -Xms32G -Xmx32G -XX:+AlwaysPreTouch -XX:MaxGCPauseMillis=10000 -XX:+DisableExplicitGC -jar \
    JEDi_Driver_MT.jar My_Parameters_PP.txt
```

- Where ‘My_Parameters_PP.txt’ is the input file to use and is passed as the first argument on the command line.

B. Organization of Output Files:

Output files from JEDi are written to subdirectories within the working directory, structured to organize the multitude of files produced in a meaningful manner. The top level of this directory tree is named "JEDi_RESULTS_**$description**", where **$description** is a user set parameter that succinctly describes the job. Limbs of the tree separate the types of PCA analysis, KPCA, FES, SSA, and VIZ, when present. Each of these in turn contains limbs for the models of PCA used, **Q** (**COV**), **R** (**CORR**), and **P (PCORR)** compartmentalization. The output file names include the **number of atoms or residues or atom-pairs** in the selected subset for reference, plus a description of the file contents.

C. Current Limitations:

Initial input of the protein trajectory must be done using PDB files that are expected to conform to the standard format, or a matrix of PDB coordinates containing the all-atom atomic positions only (see below for a description of this file). In JED, only carbon-alpha atomic positions were used to create the coordinates matrix for essential dynamic analysis.
In JEDi, all-atom atomic positions are used to create the coordinates matrix for essential dynamic analysis.

During pre-processing, **be sure that each PDB file has the same residues and atoms, in the same order**. If other files in the working directory do not match exactly, then the array sizes will not match and the program will crash. If atomic ordering is not consistent, then the arrays will be the right size but contain garbled information.

- *If JEDi crashes during the reading of PDB files, this is probably the reason.*

- *In this situation, we HIGHLY recommend that the parity check is used.*

During analytical runs, the indices for addressing the matrix of coordinates is determined from the specified Reference PDB file. JEDi will attempt to verify that the two match.

While JEDi can process a PDB file with missing residues and various numbering schemes, it can NOT interpret files that have alternate conformations within a given frame based on fractional **occupancy** values.
Only a single conformation per frame is allowed.

Note that the original residue coordinates in the PDB files are mapped to the rows of the coordinates matrix.
A block {X}{Y}{Z} packing is done:
For ***N*** *atoms*, there will be ***N x-coordinate*** rows, ***N y-coordinate*** rows, and ***N z-coordinate*** rows, in that order.

IV. Getting Started:

A. The Pre-Processing Run:

A Preliminary **Pre-Processing Run** should be performed to generate the JEDi formatted coordinate matrix file for all the atoms in the PDB files. This makes subsequent subset analyses much faster to perform. It also serves to guarantee that the specified atoms/residues for subset selection are correctly represented in matrix form. After this initialization step, the PDB files can be deleted, with the exception of the reference PDB file. Once the coordinate matrix is created, it should be used for all subsequent analyses, using different residue subsets and different job parameters.

The name of the coordinate file matrix produced from the PDB files is: `"original_PDB_coordinates_AA.txt"`
The matrix packing is as follows:
Rows are coordinate variables and columns are frames.
For N *atoms*, there are 3N rows: N x-coordinates, N y-coordinates, and N-z coordinates, stacked in that order.

- **The file to use in all subsequent JEDi analyses is the original_PDB_coordinates_AA matrix.**

  This matrix contains all the atoms in the PDB files and thus can be used for any subset of residues. When a subset is chosen, a new correspondence set is generated and a new transformation is done to optimize the alignment of the structures. This removes overall translation and rotation for each subset chosen.

In subsequent analyses, it is critical that no residues are requested that do not actually exist in the PDB file.
JEDi maps the specified residue list to an internal list that is aligned to the rows of the coordinates matrix.
JEDi generates a residue list file for all residues it finds in the PDB files that it reads.
This file should be used and edited with care when specifying residue subsets.

B. Common Causes for JEDi to Crash or Exit:

- A **KEY=VALUE PAIR** is missing
- A key in a **KEY=VALUE PAIR** is missing its value
- A specified **directory** or **file** cannot be found
- If **unexpected format** is found in **any** of the input files (reference PDB, residue list)

Note that the JEDi driver programs employ many checks during the reading of the input files and the execution of the program. There are checks to validate the number formats of numeric data. There are checks to ensure that the input files have the correct format/number of columns. There are checks to ensure that the number of modes requested does not exceed the actual number of modes available. JEDi also verifies that directories and files exist before performing any analysis. In many cases, missing or problematic parameter settings are set to a default value. The developers have attempted to provide meaningful information when the program crashes to facilitate making the necessary corrections. The input file is logged and echoed to ***standard out***, as are the assignment of parameters, while any detected problems or errors have their messages directed to ***standard error***. In the case that a Java runtime exception is thrown, a stack trace will also be sent to standard error. Please refer to the **Appendix** for help in creating properly formatted input files.

V. Running Jobs Using JEDi DRIVER

A. The Pre-Processing Run

This pre-processing step will read all PDB files in the working directory, but will perform **no PCA.**
The purpose of this is to generate the matrix of coordinates for performing subset analyses efficiently.

The PDB files (including the PDB reference file) must be in the working directory.

i. Parameters to set in the parameters file:

```
# ------------------------------------------------------------------------------------------
#     DIRECTORY, DESCRIPTION, REFERENCE_PDB
# ------------------------------------------------------------------------------------------
DIRECTORY=/working/directory/
DESCRIPTION=TEST
REFERENCE_PDB=test.pdb
# ------------------------------------------------------------------------------------------
#     PRE-PROCESSING
# ------------------------------------------------------------------------------------------
doPREPROCESS=true
doREAD_ARCHIVE=false
ARCHIVE_NAME=pdb_archive.zip
# ------------------------------------------------------------------------------------------
#     STATISTICAL THRESHOLDING
# ------------------------------------------------------------------------------------------
VARIANCE_THRESHOLD=1.000
SKEW_THRESHOLD=2.000
KURTOSIS_THRESHOLD=8.000
# ------------------------------------------------------------------------------------------
doOutputCoordinates=true
# ------------------------------------------------------------------------------------------
```

- **KEY=VALUE PAIRS SHOWN IN RED ARE RELEVENT TO THE PRE-PROCESSING RUN**

Key Points:
- The **doPREPROCESS** parameter **MUST** be set to **true** to perform pre-processing runs
- If reading form a compressed archive (ZIP or TAR), the **doREAD_ARCHIVE** parameter must be set to true
- If **doREAD_ARCHIVE=true,** then the archive name must be given by: **ARCHIVE_NAME=archive.zip**
- To get the aligned coordinates also, set **doOutputCoordinates=true**

ii. Output Files:

These are written to the **root** of the JEDi Results directory tree: `/working/directory/JEDi_Results_Description/`

- **JEDi LOG** providing a summary of the job parameters and results: `JEDi_LOG_`date+time`.txt`
- **PDB READ LOG** listing all the PDB files read, in the order they were read: `PDB_READ_Log.txt`
- **Coordinates matrix** from all the all-atom coordinates in the PDB files: `original_PDB_coordinates_AA.txt`
- **Aligned Coordinates matrix** (to the reference structure): `aligned_PDB_coordinates_AA.txt`
- **A list of all residues** found in the PDB files for subsequent editing and use: `All_PDB_Residues_JEDi.txt`
- **Files** with **counts** of **all atoms** and **heavy atoms** per residue
- **Files and Plots of Conformation RMSDs** and **Atomic RMSF**
- **Edited PDB file** containing all the atoms with the RMSF replacing B-factors
- **Files** and **Plots** the **coordinate statistics** in the PDB files
- **Files** of atom lists and residue lists that exceed the specified stat threshold

Note: JEDi creates subset files with the format: "ss_numResidues_numAtoms_.......txt"

B. Performing Production Runs

i. Parameters to set in the parameters file depend on what you want to do.

Key Points:

- **The working directory must contain:**
  - **The input file**
  - **The coordinates matrix**
  - **The PDB reference file, and**
  - **The residue lists.**

- The **doPREPROCESS** parameter **MUST** be set to **false** to perform production runs
- Set the logical switches to **true** to perform the selected action
- For each subset, specify the residue list
- **Make sure there are no spaces in the KEY=VALUE declarations**

ii. Root Output Files:

These are written to the **root** of the JEDi Results directory tree: `/working/directory/JEDi_Results_Description/`

- **The JEDi LOG** providing a summary of the job parameters and results:
  - `` JEDi_LOG_`date+time`.txt ``
- Numbers of atoms in residues: `numbers_Of_Atoms_in_Residues.txt`
- Numbers of heavy atoms in residues: `numbers_Of_Heavy_Atoms_in_Residues.txt`
- Aligned coordinates for chosen subsets (optional)
- RMSF edited PDB files: ex) `ss_xxx_AA_RMSF_edited.pdb`
- Plots of the atomic stats, RMSF, and RMSD
- Atom and residue lists of the statistical-thresholded atoms and residues (variance, skew, kurtosis)

iii. Process Dependent Output Files:

- PCA analyses are written to the **/type_PCA/model_PCA subdirectories** of the JEDi Results directory tree
- KPCA analyses are written to the **/KPCA/type_PCA/model_PCA subdirectories** of the JEDi Results directory tree
- FES analyses are written to the **/FES /type_PCA/model_PCA subdirectories** of the JEDi Results directory tree
- VIZ outputs are written to the **/VIZ /type_PCA/model_PCA subdirectories** of the JEDi Results directory tree
- SSA outputs are written to the **/SSA /type_PCA/model_PCA subdirectories** of the JEDi Results directory tree

iv. Residue List Format

The easiest way to create a residue list is to edit the JEDi generated file that lists all residues found in the PDB files

(This is already in the proper format): "*All_PDB_Residues_JEDi.txt*"

TWO columns: Column 1 Strings, Column 2 integers (tab separated): Chain IDs, residue numbers

Note that all entries in the residue list are checked against the reference PDB file.

If a requested residue cannot be found in the reference file, then JEDi will exit with an error message stating that the requested residue could not be found.

v. Atom List Format for Distance Pairs

Atom pairs are specified one per line in the atom pair list file:

Four columns of strings and integers, tab separated:

ChainID1 Atom_Number1 Chain ID2 Atom_Number2

Note that all entries in the atom pair list are checked against the reference PDB file.

If a requested atom cannot be found in the reference file, then JEDi will exit with an error message stating that the requested atom could not be found.

C. Debugging Crashes: Fixing and Preventing Problems:

i. Simple mistakes:

a) General:

1) Does every **KEY** have its **VALUE**?
2) Are there any **SPACES** in the KEY=VALUE statement?
3) Is the **doPREPROCESS** parameter set correctly?
   - Are you requesting to read PDBs when doing an analytical run?
4) Does the specified path to the input file exist?
5) Is the input file in the specified location?
6) Are **number formats** correct? (20.0 is NOT an integer)
7) Does the reference PDB file correspond exactly to the trajectory?

b) Pre-Processing Runs:

1) Does the working directory exist?
2) Does the working directory end with the file separator character ("**/**" or "****")?
3) Does the working directory contain PDB files with different entries?
   - **This is always results in a fatal error.**
4) Are the files or archive in a supported format?
5) Does the working directory contain the reference PDB file?

c) Production Runs:

1) Does the working directory contain all requested atom and residue list files?
2) Are you requesting residues that are not in the reference PDB?
3) Are you requesting atoms that are not in the reference PDB?
4) Are you requesting analyses that exceed the available compute resources?
   - If you get a "***java.lang.OutOfMemoryError: Java heap space***" error, then you need to have more RAM to perform the requested analyses. Also consider optimizing the JVM.

ii. More subtle issues:

- PDB files in non-standard format
- PDB files with fractional occupancy data
- PDB files with 2 chains, but no chain IDs or missing chain IDs

A single chain PDB file with no chain IDs will be successfully processed by JEDi, as it will add the default chain ID of 'A'.

- This will not be the case with 2 or more chains
- If the PDB file names are sorted in a different order than how they were generated, then the conformation RMSD results will not reflect what actually occurred in the simulation.
- NOTE: The Unix `**tar**` program places files in the archive randomly by default. Be sure to use the command line option "**-T**" to pass a sorted file list to the program to prevent the randomization. (Unix `**zip**` does not have this issue)

Naming the PDB files appropriately by padding the numbers with leading zeros will ensure proper sorting to prevent this problem caused by the operating system.

If the conformation RMSD is very different from what you expect, then you may be using PDB files that contain occupancy information. JEDi does not use that information. Your results will not be accurate.

- **If you have pooled data, make sure the combined matrix is constructed the way you think it is.**

Did you request Outlier Processing with a very high Z Score (>=3.00)?

- For example (dpPCA): You may have few or no samples that remain after adjusting to mean. In this case, you can end up with a singular covariance matrix. JEDi will attempt to perform the analysis with some corrections, but the program may still crash, and the results if obtained, will not be useful.

  In general, you should NOT use extreme Z or MAD scores as there is a monotonically decreasing number of samples in the outlier category as the cutoff increases, leading to less and less useful analyses**.**

Did you request more PCA modes than actually exist?

- For example (cPCA):

  If your residue subset contains 12 atoms and you ask for 50 modes, then you are going get error messages: ***Because there are only 36 modes available.***

- For example (dpPCA):

  If your Distance Pairs List contains 5 pairs and you request 10 modes, then you are going to get error messages: ***Because there are only 5 distance-pair modes available.***

In the above cases, JEDi will attempt to reset the offending value.

iii. Very subtle issues:

If your trajectory contains pooled PDB files:

- **It is critical the every file (frame) have the EXACT SAME ORDERING of the atoms.**

Atoms can be **ordered differently** when using PDB files generated from different programs or the same program with different command options. If the files have the same number of lines, but different orders, then the matrix of coordinates will be corrupted and not useable. This will not cause JEDI to fail, but will cause the results to be garbage. Please use the parity check to prevent these problems.

D. Performing Mode Visualization

To activate the Essential PCA Mode Visualization, you need to:

- set **`doEssentialViz=true`**

To activate the Individual Mode Visualization, you need to:

- set **doModeViz = true**

Recommended values for the mode amplitude are **near 0.25** depending on the system. JEDi uses a linear scaling in assigning the amount of mode displacement that is directly proportional to the number of atoms.

The VIZ outputs include all the structures for the Essential Mode visualization and the top individual modes chosen for visualization. JEDi will permute the reference structure for a given subset along the top eigenvectors (using a sine function) selected for visualization and output 10 structures (PDBs) that capture one cycle of this motion. Additionally, JEDi will also compute the superposition of the top modes deemed 'essential' (suggest limited to the top ten).

The amplitude of the motion is controlled by the value of the **VIZ_MODE_SCALE_FACTOR**, whose default value is **0.25**, and can be adjusted as necessary. Setting the value too high could cause Visualization software like PyMol™ to break the ribbon diagrams of the structures, or yield bizarre distortions of the molecule. Ultimately, the magnitude of the displacements is dependent on the magnitude of the eigenvector components for any given atom. Setting the mode amplitude between 0.25 and 0.50 is usually safe, but for proteins with highly mobile regions like loops, you may need to adjust the mode amplitude. This is done for the modes from all three models.

Other parameters and their default values:

- VIZ_MODE_SCALE_FACTOR=0.250
- LOG_FLOOR=1.000E-4
- numberModeCycles=1
- numberModeFrames=10
- numberModeComponents=10
- numberEssentialCycles=3
- numberEssentialFrames=30

Additionally, PyMol™ scripts are generated to animate those structures into a movie for visual analysis of the physical meanings of the top modes.

These files will be located in the **/VIZ** subdirectory of the root of the JEDi results tree:
`/working/directory/JEDi_Results_Description/VIZ/type_of_PCA/Model_of_PCA`

VI. Additional Types of Analysis

A. Pooling Data:

It is often useful to pool trajectory statistics. This can be done in JEDi by combining coordinate files and then performing the usual analysis. To combine the coordinate files, there is a utility program called **POOL_Driver.java** that will combine multiple matrices into one. Each matrix is appended to the last column of the preceding matrix. Of course, the number of rows in the coordinate files must match.

The matrices to combine are specified by an input file called **POOL.txt** that the user must construct correctly.

i. Run Command:

```
java -d64 Pool_Driver.java "/path/to/POOL.txt"
java -jar -d64 Pool_Driver.jar "/path/to/POOL.txt"
```

ii. Input File format:

LINE 1 specifies the number of jobs (integer)
LINE 2 is a separator line ***********************************
Then for each job you must specify the following:
The number of matrices to combine (integer)
The job description (String)
The output directory (string ending in "/" or "\\")
The path to each matrix (String)
A separator line at end of job declaration ***************************************

Sample "POOL.txt":

```
-------------------------------------------------------------------------------
2
*******************************************************************************
3
description1
/output/directory1/
/path/to/first/coords/matrix/original_PDB_Coordinates.txt
/path/to/second/coords/matrix/original_PDB_Coordinates.txt
/path/to/third/coords/matrix/original_PDB_Coordinates.txt
*******************************************************************************
4
description2
/output/directory2/
/path/to/first/coords/matrix/original_PDB_Coordinates.txt
/path/to/second/coords/matrix/original_PDB_Coordinates.txt
/path/to/third/coords/matrix/original_PDB_Coordinates.txt
/path/to/fourth/coords/matrix/original_PDB_Coordinates.txt
*******************************************************************************
-------------------------------------------------------------------------------
```

Notes:
This file specifies 2 jobs, with 3 matrices to combine for job 1, and 4 matrices to combine for job 2.
Be sure that each path and matrix file exists.

----

iii. Output File format:

The output is a single, augmented matrix with the same number of rows as the composite matrices and columns equal to the sum of all columns in the composite matrices.

The output file name for job 1 is: **Pooled Coordinates Matrix $description1 $*number_of_input_matrices*.txt**

(`Pooled_Coordinates_Matrix_$description_3.txt` for the example).

B. Subspace Analysis:

Once JEDi Driver has been run on multiple trajectories, as well as pooled trajectories, an analysis can be done to compare how similar the essential subspaces derived from those trajectories are to each other. JEDi contains a program called **Subspace_Analysis.java** along with 3 driver programs that perform those functions. The core program takes as input two matrices of eigenvectors derived from PCA (or NMA, ANM, etc.). **The matrices must have the same number of rows and columns, meaning the vectors being compared come from the same vector space and that the subspaces have the same dimensions**. For example, in an analysis of lysozyme you might choose to process 20 cPCA modes while examining 10 different experimental conditions, plus pooled data. As long as all the subsets in the analysis are the same, then all the 20 dimensional subspaces can be compared.

Like most of the JEDi programs, the subspace analysis program driver reads an input file called **SSA.txt** to obtain runtime information. This file must be constructed properly to perform the analysis correctly. The three driver programs are **SSA_Driver.java**, **FSSA_Driver.java**, and **FSSA_Iterated_Driver.java** and are different in how much analysis is requested. The SSA_Driver gives full outputs for non-iterated subspace comparison including both log files and individual flat files. The FSSA_Driver is a light-weight version with only RMSIP and PA output in the log files. The Iterated version performs a recursive variation of the above where all equidimensional subspaces are compared up to the size that was provided, for example, from 1 to 20 by step-size 1 for a 20 column input file.

i. Run Commands:

```
java -jar -d64 SSA_Driver.jar "/path/to/SSA.txt"
java -jar -d64 FSSA_Driver.jar "/path/to/SSA.txt"
java -jar -d64 FSSA_Iterated_Driver.jar "/path/to/SSA.txt"
```

ii. Input File format:

ALL three drivers use the same input file (only the outputs are different)

The format for SSA.txt is shown below:

LINE 1: Number_of_Jobs (integer)
LINE 2: Output_Directory (string ending in "/" or "\\")
LINE 3: Batch_Decription (string)
LINE 3: Separator Line **
THEN FOR EACH JOB:
Description (string)
$directory1 (string ending in "/" or "\")** **$eigenvectors1 (string)
$directory2 (string ending in "/" or "\")** **$eigenvectors2 (string)

Separator Line ***

Sample "SSA.txt":

```
--------------------------------------------------------------------------------
5
/output/directory/
MV_PCA_Model_Test
********************************************************************************
MV1
/path/to/first/eigenvector/matrix/              ss_946_top_20_eigenvectors_COV.txt
/path/to/second/eigenvector/matrix/             ss_946_top_20_eigenvectors_CORR.txt
********************************************************************************
MV2
/path/to/first/eigenvector/matrix/              ss_946_top_20_eigenvectors_COV.txt
/path/to/second/eigenvector/matrix/             ss_946_top_20_eigenvectors_CORR.txt
********************************************************************************
MV3
/path/to/first/eigenvector/matrix/              ss_946_top_20_eigenvectors_COV.txt
/path/to/second/eigenvector/matrix/             ss_946_top_20_eigenvectors_CORR.txt
********************************************************************************
MV4
/path/to/first/eigenvector/matrix/              ss_946_top_20_eigenvectors_COV.txt
/path/to/second/eigenvector/matrix/             ss_946_top_20_eigenvectors_CORR.txt
********************************************************************************
MV5
/path/to/first/eigenvector/matrix/              ss_946_top_20_eigenvectors_COV.txt
/path/to/second/eigenvector/matrix/             ss_946_top_20_eigenvectors_CORR.txt
********************************************************************************
--------------------------------------------------------------------------------
```

C. Free Energy Surface:

JEDi contains a program called FES_Driver.java that takes two DVPs as order parameters (OP) to calculate free energy (FE) using a 2-D kernel density estimate (KDE) derived from Gaussian kernels. The output file is three columns: OP1, OP2, FE. This output can be used to plot a free energy surface with respect to the selected PCA modes. The FES can be done for each type of PCA and each PCA model. The user specifies the OPs, the number of conformations to use, the offset into the points (which determines the points to use in the KDE), and the size of the 2D grid elements for making the KDE. Additionally, the program outputs a PNG image of the data as a 3D scatterplot.
All required information is specified in the input file FES.txt

i. Run Commands:

```
java -jar -d64 FES_Driver.jar “/path/to/FES.txt”
```

```
java -d64 FES_Driver.java “/path/to/FES.txt”
```

ii. Input File format:

The format for FES.txt is shown below:

LINE 1: Number_of_Jobs (integer)
LINE 2: Output_Directory (string ending in "/" or "\")
LINE 3: Batch_Decription (string)
LINE 3: Separator Line ***
THEN FOR EACH JOB:
Description (string)
$directory1 (string ending in "/" or "\")
$delta_vectors (string)
$OP1 (int) $OP2 (int) $num_points (int) $offset (int) $cellsize (double)

Separator Line ***

Sample "FES.txt":

```
------------------------------------------------------------------------------------
2
/output/directory/
Test_FES_Pooled_Data
*****************************************************************************************************
Test_First_Half
/working/directory1/
ss_151_top_2_DVPs_CORR.txt
0     1     1001     0     0.00
*****************************************************************************************************
Test_Second_Half
/working/directory1/
ss_151_top_2_DVPs_CORR.txt
0     1     1000     1000     0.00
*****************************************************************************************************
------------------------------------------------------------------------------------
```

Notes:

Recommended values for cellsize is [0.01, 0.05]

Set $size = 0 to determine automatically, using the Max (Range X, Range Y)/256

D. Kernel PCA:

JEDi contains a program called KPCA_Driver.java that takes as input the top PCs or DVPs from the PCA analyses. This program runs a new PCA analysis using a selection of kernels on the dimension-reduced generalized coordinates. The user selects the number of inputs, the kernels to use, and the number of output kernel PCs.
All required information is specified as **KEY=VALUE** pairs in the in the input file: KPCA_Parameters.txt

i. Run Commands:

```
java -jar -d64 KPCA_Driver.jar /path/to/KPCA_Parameters.txt

java -d64 KPCA_Driver.java /path/to/KPCA_Parameters.txt
```

ii. Input File format:

The format for KPCA_Parameters.txt is shown below:

```
-----------------------------------------------------------------------------------------------
DIRECTORY=C:\Users\workspace\JEDi\test\BL\JEDi_RESULTS_TEST_BL\Alpha_Carbon_PCA\COV\
OUT_DIRECTORY=C:\Users\workspace\JEDi\test\BL\KPCA\
DESCRIPTION=TEST_KPCA
PROJECTIONS=ss_263_top_5_normed_DVPs_Alpha_Carbon_PCA.txt
NUMBER_KPCs=3
do_linear_kernel=false
do_Degree_2_Poly=false
do_Degree_3_Poly=false
do_Degree_4_Poly=false
do_XY_Poly=false
do_Poly_Diff_Sq=false
do_Poly_Diff_Cubes=false
do_Euclidean=false
do_Gaussian=true
do_Neural_Net=true
do_MI=true
do_MI_KDE=true
do_SinCos=false
SLOPE=100
SIGMA=1.000
-----------------------------------------------------------------------------------------------
```

Notes:
Set SLOPE and SIGMA according to the variance in the data.
Make sure that the output directory exists prior to running the program.

iii. Output

The output includes the kernel PCs and scatterplots of the top two KPCs.

E. Essential Mode Visualization:

JEDi contains a program called VIZ_Driver.java that allows the user to view not only individual modes, but the superposition of all the modes deemed 'essential'. A starting mode is specified so that the user can window over sets of modes. The number of modes to visualize determines the size of that window. Also, the user can specify a threshold (low/high) that can be adjusted to enhance the log coloring scheme. With thresholding, a percentage of frames are set to the minimum and maximum values, broadening the coloring of the inter-threshold range. The mode amplitude parameter determines the amount of displacement from equilibrium for visualizing the modes. Selecting the number of frames and number of cycles controls the level of detail captured in the movies.
All required information is specified in the input file VIZ.txt

i. Run Commands:

```
java -jar -d64 VIZ_Driver.jar "/path/to/VIZ.txt"
java -d64 VIZ_Driver.java "/path/to/VIZ.txt"
```

ii. Input File format:

The format for VIZ.txt is shown below:

LINE 1: Number_of_Jobs (integer)
LINE 2: Separator Line **
THEN FOR EACH JOB:
$start_mode $num_modes_viz $mode_amplitutude $threshold_low $threshold_high $num_frames $num_cycles $do_individual $type

$ref_PDB

$eigenvalues

$eigenvectors

$sq_modes

$mode_maxes

$mode_mins

$out_dir (string ending in "/" or "\\")

Separator Line **

Sample "VIZ.txt":

```
--------------------------------------------------------------------------------
2
***************************************************************************************************************
1     1     3.0     .05     .05     100     5     0     PCORR
/path/to/ss_151_RMSF_edited.pdb
/path/to/ss_151_eigenvalues_PCORR.txt
/path/to/ss_151_top_20_eigenvectors_PCORR.txt
/path/to/ss_151_top_20_square_pca_modes_PCORR.txt
/path/to/ss_151_top_20_square_pca_mode_MAXES_PCORR.txt
/path/to/ss_151_top_20_square_pca_mode_MINS_PCORR.txt
/output/directory/VIZ_TEST_PCORR/
***************************************************************************************************************
1     1     3.0     .05     .05     100     5     0     CORR
/path/to/ss_151_RMSF_edited.pdb
/path/to/ss_151_eigenvalues_CORR.txt
/path/to/ss_151_top_20_eigenvectors_CORR.txt
/path/to/ss_151_top_20_square_pca_modes_CORR.txt
/path/to/ss_151_top_20_square_pca_mode_MAXES_CORR.txt
/path/to/ss_151_top_20_square_pca_mode_MINS_CORR.txt
/output/directory/VIZ_TEST_CORR/
***************************************************************************************************************
--------------------------------------------------------------------------------
```

iii. Output:

The output is a set of **$num_frames** PDBs for the individual modes, if requested by setting **doModeViz** to **true**.

A set of 30 PDBs for the Essential Modes (default value is 30 frames)

A PyMol™ script is generated to animate each set of PDB files (.pml files).

Make sure that the output directory exists prior to running the program.

VII. Appendix 1 - Input File Formats

A. JEDi_Parameters.txt

```
# Lines beginning with "#" are comments.
# -------------------------------------------------------------
#     DIRECTORY, DESCRIPTION, REFERENCE_PDB
# -------------------------------------------------------------
DIRECTORY=/user/xxxxx/workspace/
DESCRIPTION=Test
REFERENCE_PDB=ss_5_81_Hierarchical_AA_RMSF_edited.pdb
# -------------------------------------------------------------
#     PRE-PROCESSING
# -------------------------------------------------------------
doPREPROCESS=false
doParityCheck=false
# -------------------------------------------------------------
doREAD_ARCHIVE=false
ARCHIVE_NAME=archive.zip
# -------------------------------------------------------------
#     STATISTICAL THRESHOLDING
# -------------------------------------------------------------
do_StatThresholds=false
# -------------------------------------------------------------
VARIANCE_THRESHOLD=1.0
SKEW_THRESHOLD=3.0
KURTOSIS_THRESHOLD=8.0
# -------------------------------------------------------------
#     COORDINATES FILE
# -------------------------------------------------------------
ORIGINAL_PDB_COORDS=Pooled_Coordinates_Matrix_BL_MD_FRODA_TEM1_TEM52_MechanisticSite_4.txt.bz2
# -------------------------------------------------------------
#     PCA SUBSETS & RESOLUTIONS
# -------------------------------------------------------------
doAA=false
doHA=false
doBB=false
doCA=false
doATOM_LIST=false
# -------------------------------------------------------------
doRESIDUE_INDIVIDUAL=true
doRESIDUE_PAIRS=true
# -------------------------------------------------------------
doHIERARCHICAL_AA=true
doHIERARCHICAL_HA=true
# -------------------------------------------------------------
doDIST_PAIRS=true
# -------------------------------------------------------------
#     PCA STATISTICAL MODELS
# -------------------------------------------------------------
doCORR=true
doPCORR=true
# -------------------------------------------------------------
#     NUMBER of PCA MODES
# -------------------------------------------------------------
MODES_RESIDUE_PAIRS=1
MODES_RESIDUE_INDIVIDUAL=20
# -------------------------------------------------------------
MODES_EIGEN_RESIDUE_AA=3
MODES_HIERARCHICAL_AA=15
# -------------------------------------------------------------
MODES_EIGEN_RESIDUE_HA=3
MODES_HIERARCHICAL_HA=15
# -------------------------------------------------------------
MODES_ALL_ATOM=20
MODES_HEAVY_ATOM=20
MODES_BACKBONE=15
MODES_ALPHA_CARBON=10
# -------------------------------------------------------------
MODES_ATOMS_LIST=20
MODES_DISTANCE_PAIRS=20
# -------------------------------------------------------------
#     RESIDUE & ATOM SUBSETS
# -------------------------------------------------------------
RESIDUE_LIST_ALL_ATOM=MechanisticSite.txt
RESIDUE_LIST_HEAVY_ATOM=MechanisticSite.txt
RESIDUE_LIST_BACKBONE=MechanisticSite.txt
RESIDUE_LIST_ALPHA_CARBON=MechanisticSite.txt
# -------------------------------------------------------------
RESIDUE_LIST_PAIRS=MechanisticSite.txt
RESIDUE_LIST_INDIVIDUAL=MechanisticSite.txt
# -------------------------------------------------------------
RESIDUE_LIST_HIERARCHICAL_AA=MechanisticSite.txt
RESIDUE_LIST_HIERARCHICAL_HA=MechanisticSite.txt
# -------------------------------------------------------------
ATOMS_LIST=MECHSITE_Most_Active_Atoms_List.txt
ATOM_PAIRS_LIST=MECHSITE_Most_Active_Atoms_Pairs.txt
# -------------------------------------------------------------
#     DOWN-SAMPLING
# -------------------------------------------------------------
doDownSample=true
# -------------------------------------------------------------
DOWNSAMPLE=25
# -------------------------------------------------------------
doFrameSelect=false
# -------------------------------------------------------------
FRAME_START=1
```

```
FRAME_END=50
# -------------------------------------------------------------
#     OUTLIER PROCESSING
# -------------------------------------------------------------
doOutlierProcessing=true
# -------------------------------------------------------------
MAD_SCORE_CUTOFF=0
Z_SCORE_CUTOFF=1.960
# -------------------------------------------------------------
#     SPARSIFICATION
# -------------------------------------------------------------
doSPARSIFY=true
# -------------------------------------------------------------
THRESHOLD_COV=0
THRESHOLD_CORR=0.500
THRESHOLD_PCORR=0.250
THRESHOLD_RP_DIFF=0.500
# -------------------------------------------------------------
#     REDUCE DYNAMIC MATRICES (3N --> N)
# -------------------------------------------------------------
doREDUCE=true
# -------------------------------------------------------------
#     FREE ENERGY LANDSCAPE
# -------------------------------------------------------------
doFES=false
# -------------------------------------------------------------
#     KERNEL PCA
# -------------------------------------------------------------
doKPCA=false
# -------------------------------------------------------------
MAX_KERNEL_FRAMES=500
NUMBER_PCs_INPUT=5
KERNEL_SHRINKAGE=0.010
KPCA_SIGMA=0
KPCA_SLOPE=0
MULTIPLIER=100
# -------------------------------------------------------------
Linear=false
Degree_2_Poly=true
Degree_3_Poly=true
Degree_4_Poly=true
XY_Poly=false
Euclidean=true
Mahalanobis=true
Gaussian=true
Sigmoid=true
Log=true
Circular=false
Cauchy=false
MI=true
MI_KDE=true
# -------------------------------------------------------------
#     MODE VISUALIZTION
# -------------------------------------------------------------
doModeViz=false
doEssentialViz=false
MODES_VIZ=5
# -------------------------------------------------------------
#     ADVANCED PARAMETERS
# -------------------------------------------------------------
FLOOR=1.000E-16
NOISE_LEVEL=1.000E-6
KDE_RESOLUTION=20
KDE_CELL=64
KDE_MARGIN=0.250
# -------------------------------------------------------------
VIZ_MODE_SCALE_FACTOR=0.250
LOG_FLOOR=1.000E-4
numberModeCycles=1
numberModeFrames=10
numberModeComponents=10
numberEssentialCycles=3
numberEssentialFrames=30
# -------------------------------------------------------------
READ_PDBS_FILTER_STRING=.pdb
# -------------------------------------------------------------
#     OUTPUT CONTROL
# -------------------------------------------------------------
verbose=false
doOutputCoordinates=false
# -------------------------------------------------------------
```

B. Residue List file

```
-----------------------------------------------------------------------------------
A       1
A       2
A       3
A       7
A       8
A       9
A       10
B       1
B       2
B       3
B       4
C       1
-----------------------------------------------------------------------------------
```

C. Atom Pair List file

```
-----------------------------------------------------------------------------------
A              62             A              227
A              65             A              320
A              120            B              570
B              18             B              80
B              360            B              1010
-----------------------------------------------------------------------------------
```

D. POOL.txt

```
--------------------------------------------------------------------------------------------
$num_jobs --> (repeat job declaration $num_jobs times)
********************************************************************************
$num_of_matrices_to_combine
$description
$output_directory
$path_to_coords_matrix1 --> (repeat line $num_of_matrices_to_combine times)
********************************************************************************
-----------------------------------------------------------------------------------------------
```

E. SSA.txt

```
--------------------------------------------------------------------------------------------
$num_jobs --> (repeat job declaration $num_jobs times)
$output_directory
$batch_description
********************************************************************************
$job_description
$path_to_first_eigenvector_file
$path_to_second_eigenvector_file
********************************************************************************
-----------------------------------------------------------------------------------------------
```

F. FES.txt

```
-----------------------------------------------------------------------------------------------
$num_jobs --> (repeat job declaration $num_jobs times)
$out_dir
$batch_description
*****************************************************
$job_description
$directory
$delta_vectors
$op1     $op2     $num_points     $offset     $cell_size
*****************************************************

-----------------------------------------------------------------------------------------------
```

G. KPCA_Parameters.txt

```
------------------------------------------------------------------------------------------
DIRECTORY=C:\Users\workspace\JEDi\test\BL\JEDi_RESULTS_TEST_BL\Alpha_Carbon_PCA\COV\
OUT_DIRECTORY=C:\Users\workspace\JEDi\test\BL\KPCA\
DESCRIPTION=TEST_KPCA
PROJECTIONS=ss_263_top_5_normed_DVPs_Alpha_Carbon_PCA.txt
NUMBER_KPCs=3
do_linear_kernel=false
do_Degree_2_Poly=false
do_Degree_3_Poly=false
do_Degree_4_Poly=false
do_XY_Poly=false
do_Poly_Diff_Sq=false
do_Poly_Diff_Cubes=false
do_Euclidean=false
do_Gaussian=true
do_Neural_Net=true
do_MI=true
do_MI_KDE=true
do_SinCos=false
SLOPE=100
SIGMA=1.000
------------------------------------------------------------------------------------------
```

H. VIZ.txt

```
------------------------------------------------------------------------------------------
$num_jobs --> (repeat job declaration $num_jobs times)
******************************************************************************************
$start_mode   $num_modes_viz   $mode_amplitutude   $threshold_low   $threshold_high   $num_frames   $num_cycles   $do_individual   $type

$ref_PDB
$eigenvalues
$eigenvectors
$sq_modes
$mode_maxes
$mode_mins
$out_dir
******************************************************************************************
------------------------------------------------------------------------------------------
```

VIII. Appendix 2 - Output File Formats:

A. Sample JEDi Log file: Abridged (…) for space considerations

```
---------------------------------------------------------------------------------------------
JEDi: Java Essential Dynamics Inspector
Release 1, September, 2019
---------------------------------------------------------------------------------------------

Parameters for the analysis:
                    DIRECTORY=C:\Users\cdavid\eclipse-workspace\JEDi_Release\test\BL\POOLED_MD_FRODA_TEM1_TEM52\ActiveSite_OmegaLoop\
                    DESCRIPTION=ActiveSite_OmegaLoop_DS10_NOP
                    REFERENCE_PDB=ss_24_376_AA_RMSF_edited.pdb
                    doPREPROCESS=false
                    doREAD_ARCHIVE=false
                    ARCHIVE_NAME=archive.zip
                    ORIGINAL_PDB_COORDS=Pooled_Coordinates_Matrix_BL_MD_FRODA_TEM1_TEM52_ActiveSite_OmegaLoop_4.txt
                    doAA=true
                    doHA=true
                    doBB=true
                    doCA=true
                    doRESIDUE_INDIVIDUAL=true
                    doRESIDUE_PAIRS=true
                    doHIERARCHICAL_AA=true
                    doHIERARCHICAL_HA=true
                    doATOM_LIST=true
                    doATOM_PAIRS=false
                    doCORR=true
                    doPCORR=true
                    MODES_RESIDUE_PAIRS=1
                    MODES_RESIDUE_INDIVIDUAL=20
                    MODES_RESIDUE_AA=3
                    MODES_HIERARCHICAL_AA=20
                    MODES_RESIDUE_HA=3
                    MODES_HIERARCHICAL_HA=20
                    MODES_ALL_ATOM=15
                    MODES_HEAVY_ATOM=15
                    MODES_BACKBONE=15
                    MODES_ALPHA_CARBON=15
                    MODES_ATOMS_LIST=21
                    MODES_DISTANCE_PAIRS=21
                    RESIDUE_LIST_ALL_ATOM=ActiveSite_OmegaLoop.txt
                    RESIDUE_LIST_HEAVY_ATOM=ActiveSite_OmegaLoop.txt
                    RESIDUE_LIST_BACKBONE=ActiveSite_OmegaLoop.txt
                    RESIDUE_LIST_ALPHA_CARBON=ActiveSite_OmegaLoop.txt
                    RESIDUE_LIST_INDIVIDUAL=ActiveSite_OmegaLoop.txt
                    RESIDUE_LIST_PAIRS=ActiveSite_OmegaLoop.txt
                    RESIDUE_LIST_HIERARCHICAL_AA=ActiveSite_OmegaLoop.txt
                    RESIDUE_LIST_HIERARCHICAL_HA=ActiveSite_OmegaLoop.txt
                    ATOMS_LIST=All_Atom_High_Kurtosis_Atoms_6.000.txt
                    ATOM_PAIRS_LIST=MECHSITE_Most_Active_Atoms_Pairs.txt
                    doDownSample=true
                    DOWNSAMPLE=10
                    doFrameSelect=false
                    FRAME_START=1
                    FRAME_END=50
                    VARIANCE_THRESHOLD=1.000
                    SKEW_THRESHOLD=2.000
                    KURTOSIS_THRESHOLD=8.000
                    doOutlierRemoval=false
                    doOutlierSelection=false
                    MAD_SCORE_CUTOFF=0
                    Z_SCORE_CUTOFF=1.000
                    doSPARSIFY=false
                    THRESHOLD_COV=0
                    THRESHOLD_CORR=0.500
                    THRESHOLD_PCORR=0.100
                    doREDUCE=true
                    doFES=false
                    doKPCA=false
                    MAX_KERNEL_FRAMES=500
                    NUMBER_PCs_INPUT=5
                    KERNEL_SHRINKAGE=0.010
                    KPCA_SIGMA=0
                    KPCA_SLOPE=0
                    MULTIPLIER=100
                    doModeViz=false
                    doEssentialViz=false
                    MODES_VIZ=10
                    READ_PDBS_FILTER_STRING=.pdb
                    FLOOR=1.000E-16
                    NOISE_LEVEL=1.000E-6
                    KDE_RESOLUTION=20
                    KDE_CELL=64
                    KDE_MARGIN=0.250
                    VIZ_MODE_SCALE_FACTOR=0.250
                    LOG_FLOOR=1.000E-4
                    numberModeCycles=1
                    numberModeFrames=10
                    numberModeComponents=10
                    numberEssentialCycles=3
                    numberEssentialFrames=30
                    doOutputCoordinates=true
```

```
-----------------------------------------------------------------------------------------------

Processed the Reference PDB file: ss_24_376_AA_RMSF_edited.pdb
                     The number of residues found in the Reference PDB file = 24
                     The number of atoms found in the Reference PDB file = 376
                     The number of Heavy atoms found in the Reference PDB file = 201
                     Created file: 'All_PDB_Residues_JEDi.txt'. This file contains all chainID-residue number pairs found in the Reference PDB file.
                     Number of atoms per residue list file created: 'numbers_Of_Atoms_in_Residues.txt'
                     Number of heavy atoms per residue list file created: 'numbers_Of_Heavy_Atoms_in_Residues.txt'
-----------------------------------------------------------------------------------------------

Read the All Atom PDB coordinates file: Pooled_Coordinates_Matrix_BL_MD_FRODA_TEM1_TEM52_ActiveSite_OmegaLoop_4.txt
                     The expected packing in this Matrix is: {X}{Y}{Z} stacking.
                     The dimension of the coordinates matrix is = 1128 by 24006
                     Total number of atoms in matrix = 376
                     Total number of conformations in matrix = 24006
                     The matching reference structure is: ss_24_376_AA_RMSF_edited.pdb
-----------------------------------------------------------------------------------------------

Down Sampled the coordinates file:
                     The down-sample-factor was: 10
                     The number of frames in the original matrix was: 24006
                     The number of frames in the reduced matrix is: 2400
-----------------------------------------------------------------------------------------------

The All_Atom_PCA Subset coordinates were obtained from: ActiveSite_OmegaLoop.txt
                     The number of residues in the subset = 24
                     The number of atoms in the subset = 376
                     The dimension of the Reference Coordinates matrix  = 1128 by 1
                     The dimension of the Coordinates matrix  = 1128 by 2400
                     The coordinates were aligned to the reference coordinates using quaternion algebra.
                     The atomic RMSFs were calculated and used to create a PDB file with B-Factors replaced with the atomic RMSF.
                     The subset atoms are:
                                          ATOM    710     N    SER  A    70     44.201   41.496   42.341    31.47   N
                                          ATOM    711     H    SER  A    70     44.801   42.306   42.231    37.13   H
                                          ATOM    712     CA   SER  A    70     42.891   41.596   42.941    30.13   C
                                          ATOM    713     HA   SER  A    70     42.951   40.946   43.811    31.46   H
                                          ATOM    714     CB   SER  A    70     42.641   43.066   43.231    37.46   C
                                          ATOM    715     HB1  SER  A    70     41.581   43.276   43.401    50.93   H
                                          ATOM    716     HB2  SER  A    70     42.861   43.676   42.361    54.26   H
                                          ATOM    717     OG   SER  A    70     43.301   43.606   44.361    34.26   O
                                          ATOM    718     HG   SER  A    70     44.251   43.456   44.271    60.98   H
                                          ATOM    719     C    SER  A    70     41.731   40.956   42.191    31.30   C
                                          ATOM    720     O    SER  A    70     40.681   40.776   42.801    45.69   O
                                          ....
                                          ....
                                          ATOM    3238    N    LYS  A    234    36.031   42.406   37.821    36.51   N
                                          ATOM    3239    H    LYS  A    234    36.241   41.856   36.991    38.52   H
                                          ATOM    3240    CA   LYS  A    234    37.021   42.706   38.841    33.93   C
                                          ATOM    3241    HA   LYS  A    234    36.791   43.646   39.331    35.58   H
                                          ATOM    3242    CB   LYS  A    234    37.081   41.606   39.891    34.34   C
                                          ATOM    3243    HB1  LYS  A    234    37.521   40.726   39.421    43.46   H
                                          ATOM    3244    HB2  LYS  A    234    36.061   41.336   40.171    46.43   H
                                          ATOM    3245    CG   LYS  A    234    37.921   41.766   41.151    39.56   C
                                          ATOM    3246    HG1  LYS  A    234    38.981   41.956   40.991    53.48   H
                                          ATOM    3247    HG2  LYS  A    234    37.821   40.876   41.791    54.98   H
                                          ATOM    3248    CD   LYS  A    234    37.341   42.806   42.101    34.99   C
                                          ATOM    3249    HD1  LYS  A    234    37.041   42.396   43.071    46.01   H
                                          ATOM    3250    HD2  LYS  A    234    36.551   43.346   41.591    37.20   H
                                          ATOM    3251    CE   LYS  A    234    38.561   43.706   42.321    31.70   C
                                          ATOM    3252    HE1  LYS  A    234    39.501   43.336   41.911    39.52   H
                                          ATOM    3253    HE2  LYS  A    234    38.771   43.746   43.391    37.28   H
                                          ATOM    3254    NZ   LYS  A    234    38.261   45.106   42.001    27.91   N
                                          ATOM    3255    HZ1  LYS  A    234    37.401   45.316   42.501    28.82   H
                                          ATOM    3256    HZ2  LYS  A    234    39.011   45.736   42.231    31.12   H
                                          ATOM    3257    HZ3  LYS  A    234    37.991   45.276   41.041    30.42   H
                                          ATOM    3258    C    LYS  A    234    38.391   42.896   38.211    33.66   C
                                          ATOM    3259    O    LYS  A    234    38.811   42.106   37.381    37.81   O
-----------------------------------------------------------------------------------------------

The Backbone_PCA Subset coordinates were obtained from: ActiveSite_OmegaLoop.txt
                     The number of residues in the subset = 24
                     The number of atoms in the subset = 96
                     The dimension of the Reference Coordinates matrix  = 288 by 1
                     The dimension of the Coordinates matrix  = 288 by 2400
                     The coordinates were aligned to the reference coordinates using quaternion algebra.
                     The atomic RMSFs were calculated and used to create a PDB file with B-Factors replaced with the atomic RMSF.
                     The subset atoms are:
                                          ATOM    710     N    SER  A    70     44.201   41.496   42.341    31.47   N
                                          ATOM    712     CA   SER  A    70     42.891   41.596   42.941    30.13   C
                                          ATOM    719     C    SER  A    70     41.731   40.956   42.191    31.30   C
                                          ATOM    720     O    SER  A    70     40.681   40.776   42.801    45.69   O
                                          ...
                                          ...
                                          ATOM    3238    N    LYS  A    234    36.031   42.406   37.821    36.51   N
                                          ATOM    3240    CA   LYS  A    234    37.021   42.706   38.841    33.93   C
                                          ATOM    3258    C    LYS  A    234    38.391   42.896   38.211    33.66   C
                                          ATOM    3259    O    LYS  A    234    38.811   42.106   37.381    37.81   O
-----------------------------------------------------------------------------------------------

The Heavy_Atom_PCA Subset coordinates were obtained from: ActiveSite_OmegaLoop.txt
                     The number of residues in the subset = 24
                     The number of atoms in the subset = 201
                     The dimension of the Reference Coordinates matrix  = 603 by 1
                     The dimension of the Coordinates matrix  = 603 by 2400
                     The coordinates were aligned to the reference coordinates using quaternion algebra.
                     The atomic RMSFs were calculated and used to create a PDB file with B-Factors replaced with the atomic RMSF.
```

```
                     The subset atoms are:
                                          ATOM    710     N    SER  A    70     44.201   41.496   42.341    31.47   N
                                          ATOM    712     CA   SER  A    70     42.891   41.596   42.941    30.13   C
                                          ATOM    714     CB   SER  A    70     42.641   43.066   43.231    37.46   C
                                          ATOM    717     OG   SER  A    70     43.301   43.606   44.361    34.26   O
                                          ATOM    719     C    SER  A    70     41.731   40.956   42.191    31.30   C
                                          ATOM    720     O    SER  A    70     40.681   40.776   42.801    45.69   O
                                          ...
                                          ...
                                          ATOM    3238    N    LYS  A    234    36.031   42.406   37.821    36.51   N
                                          ATOM    3240    CA   LYS  A    234    37.021   42.706   38.841    33.93   C
                                          ATOM    3242    CB   LYS  A    234    37.081   41.606   39.891    34.34   C
                                          ATOM    3245    CG   LYS  A    234    37.921   41.766   41.151    39.56   C
                                          ATOM    3248    CD   LYS  A    234    37.341   42.806   42.101    34.99   C
                                          ATOM    3251    CE   LYS  A    234    38.561   43.706   42.321    31.70   C
                                          ATOM    3254    NZ   LYS  A    234    38.261   45.106   42.001    27.91   N
                                          ATOM    3258    C    LYS  A    234    38.391   42.896   38.211    33.66   C
                                          ATOM    3259    O    LYS  A    234    38.811   42.106   37.381    37.81   O
-----------------------------------------------------------------------------------------------

The Alpha_Carbon_PCA Subset coordinates were obtained from: ActiveSite_OmegaLoop.txt
                     The number of residues in the subset = 24
                     The number of atoms in the subset = 24
                     The dimension of the Reference Coordinates matrix  = 72 by 1
                     The dimension of the Coordinates matrix  = 72 by 2400
                     The coordinates were aligned to the reference coordinates using quaternion algebra.
                     The atomic RMSFs were calculated and used to create a PDB file with B-Factors replaced with the atomic RMSF.
                     The subset atoms are:
                                          ATOM    712     CA   SER  A    70     42.891   41.596   42.941    30.13   C
                                          ATOM    757     CA   LYS  A    73     38.981   38.846   45.331    21.30   C
                                          ATOM    1637    CA   SER  A    130    39.941   46.456   45.761    29.63   C
                                          ATOM    1648    CA   ASP  A    131    40.571   46.706   49.591    28.95   C
                                          ATOM    1708    CA   ASN  A    136    43.081   39.336   54.121    30.62   C
                                          ATOM    2130    CA   ASP  A    163    49.871   31.006   48.291    14.79   C
                                          ATOM    2142    CA   ARG  A    164    50.421   34.556   49.631    13.95   C
                                          ATOM    2166    CA   TRP  A    165    48.361   37.386   51.321    20.94   C
                                          ATOM    2190    CA   GLU  A    166    47.981   41.026   50.191    16.10   C
                                          ATOM    2213    CA   PRO  A    167    50.281   42.786   49.341    27.91   C
                                          ATOM    2219    CA   GLU  A    168    52.841   39.966   48.951    34.16   C
                                          ATOM    2234    CA   LEU  A    169    50.751   38.156   46.351    30.39   C
                                          ATOM    2253    CA   ASN  A    170    51.211   41.036   43.821    32.32   C
                                          ATOM    2267    CA   GLU  A    171    54.921   40.336   43.511    29.50   C
                                          ATOM    2282    CA   ALA  A    172    54.841   38.766   40.001    48.80   C
                                          ATOM    2292    CA   ILE  A    173    58.301   37.076   39.741    33.82   C
                                          ATOM    2319    CA   PRO  A    174    58.941   35.636   36.221    38.40   C
                                          ATOM    2325    CA   ASN  A    175    57.971   32.006   35.911    34.29   C
                                          ATOM    2339    CA   ASP  A    176    56.931   31.956   39.641     7.66   C
                                          ATOM    2351    CA   GLU  A    177    53.631   30.136   39.701    13.37   C
                                          ATOM    2366    CA   ARG  A    178    52.591   31.516   43.041    16.06   C
                                          ATOM    2390    CA   ASP  A    179    49.231   33.436   43.041    20.00   C
                                          ATOM    3228    CA   ASP  A    233    34.061   42.806   36.521    44.53   C
                                          ATOM    3240    CA   LYS  A    234    37.021   42.706   38.841    33.93   C
-----------------------------------------------------------------------------------------------

The Residue_All_Atom_PCA Subset coordinates were obtained from: ActiveSite_OmegaLoop.txt
                     The number of residues in the subset = 24
                     The number of atoms in the subset = 376
                     The dimension of the Reference Coordinates matrix  = 1128 by 1
                     The dimension of the Coordinates matrix  = 1128 by 2400
                     The coordinates were aligned to the reference coordinates using quaternion algebra.
                     The atomic RMSFs were calculated and used to create a PDB file with B-Factors replaced with the atomic RMSF.
                     The subset atoms are:
                                          ATOM    710     N    SER  A    70     44.201   41.496   42.341    31.47   N
                                          ATOM    711     H    SER  A    70     44.801   42.306   42.231    37.13   H
                                          ATOM    712     CA   SER  A    70     42.891   41.596   42.941    30.13   C
                                          ATOM    713     HA   SER  A    70     42.951   40.946   43.811    31.46   H
                                          ATOM    714     CB   SER  A    70     42.641   43.066   43.231    37.46   C
                                          ATOM    715     HB1  SER  A    70     41.581   43.276   43.401    50.93   H
                                          ATOM    716     HB2  SER  A    70     42.861   43.676   42.361    54.26   H
                                          ATOM    717     OG   SER  A    70     43.301   43.606   44.361    34.26   O
                                          ATOM    718     HG   SER  A    70     44.251   43.456   44.271    60.98   H
                                          ATOM    719     C    SER  A    70     41.731   40.956   42.191    31.30   C
                                          ATOM    720     O    SER  A    70     40.681   40.776   42.801    45.69   O
                                          ...
                                          ...
                                          ATOM    3238    N    LYS  A    234    36.031   42.406   37.821    36.51   N
                                          ATOM    3239    H    LYS  A    234    36.241   41.856   36.991    38.52   H
                                          ATOM    3240    CA   LYS  A    234    37.021   42.706   38.841    33.93   C
                                          ATOM    3241    HA   LYS  A    234    36.791   43.646   39.331    35.58   H
                                          ATOM    3242    CB   LYS  A    234    37.081   41.606   39.891    34.34   C
                                          ATOM    3243    HB1  LYS  A    234    37.521   40.726   39.421    43.46   H
                                          ATOM    3244    HB2  LYS  A    234    36.061   41.336   40.171    46.43   H
                                          ATOM    3245    CG   LYS  A    234    37.921   41.766   41.151    39.56   C
                                          ATOM    3246    HG1  LYS  A    234    38.981   41.956   40.991    53.48   H
                                          ATOM    3247    HG2  LYS  A    234    37.821   40.876   41.791    54.98   H
                                          ATOM    3248    CD   LYS  A    234    37.341   42.806   42.101    34.99   C
                                          ATOM    3249    HD1  LYS  A    234    37.041   42.396   43.071    46.01   H
                                          ATOM    3250    HD2  LYS  A    234    36.551   43.346   41.591    37.20   H
                                          ATOM    3251    CE   LYS  A    234    38.561   43.706   42.321    31.70   C
                                          ATOM    3252    HE1  LYS  A    234    39.501   43.336   41.911    39.52   H
                                          ATOM    3253    HE2  LYS  A    234    38.771   43.746   43.391    37.28   H
                                          ATOM    3254    NZ   LYS  A    234    38.261   45.106   42.001    27.91   N
                                          ATOM    3255    HZ1  LYS  A    234    37.401   45.316   42.501    28.82   H
                                          ATOM    3256    HZ2  LYS  A    234    39.011   45.736   42.231    31.12   H
                                          ATOM    3257    HZ3  LYS  A    234    37.991   45.276   41.041    30.42   H
                                          ATOM    3258    C    LYS  A    234    38.391   42.896   38.211    33.66   C
                                          ATOM    3259    O    LYS  A    234    38.811   42.106   37.381    37.81   O
```

```
-----------------------------------------------------------------------------------------------

The Residue_Pair_Analysis Subset coordinates were obtained from: ActiveSite_OmegaLoop.txt
                    The number of residues in the subset = 376
                    The dimension of the Reference Coordinates matrix  = 1128 by 1
                    The dimension of the Coordinates matrix  = 1128 by 2400
                    The coordinates were aligned to the reference coordinates using quaternion algebra.
                    The atomic RMSFs were calculated and used to create a PDB file with B-Factors replaced with the atomic RMSF.
                    The subset atoms are:
                                        ATOM    710     N    SER  A    70     44.201   41.496   42.341    31.47   N
                                        ATOM    711     H    SER  A    70     44.801   42.306   42.231    37.13   H
                                        ATOM    712     CA   SER  A    70     42.891   41.596   42.941    30.13   C
                                        ATOM    713     HA   SER  A    70     42.951   40.946   43.811    31.46   H
                                        ATOM    714     CB   SER  A    70     42.641   43.066   43.231    37.46   C
                                        ATOM    715     HB1  SER  A    70     41.581   43.276   43.401    50.93   H
                                        ATOM    716     HB2  SER  A    70     42.861   43.676   42.361    54.26   H
                                        ATOM    717     OG   SER  A    70     43.301   43.606   44.361    34.26   O
                                        ATOM    718     HG   SER  A    70     44.251   43.456   44.271    60.98   H
                                        ATOM    719     C    SER  A    70     41.731   40.956   42.191    31.30   C
                                        ATOM    720     O    SER  A    70     40.681   40.776   42.801    45.69   O
                                        ...
                                        ...
                                        ATOM    3238    N    LYS  A    234    36.031   42.406   37.821    36.51   N
                                        ATOM    3239    H    LYS  A    234    36.241   41.856   36.991    38.52   H
                                        ATOM    3240    CA   LYS  A    234    37.021   42.706   38.841    33.93   C
                                        ATOM    3241    HA   LYS  A    234    36.791   43.646   39.331    35.58   H
                                        ATOM    3242    CB   LYS  A    234    37.081   41.606   39.891    34.34   C
                                        ATOM    3243    HB1  LYS  A    234    37.521   40.726   39.421    43.46   H
                                        ATOM    3244    HB2  LYS  A    234    36.061   41.336   40.171    46.43   H
                                        ATOM    3245    CG   LYS  A    234    37.921   41.766   41.151    39.56   C
                                        ATOM    3246    HG1  LYS  A    234    38.981   41.956   40.991    53.48   H
                                        ATOM    3247    HG2  LYS  A    234    37.821   40.876   41.791    54.98   H
                                        ATOM    3248    CD   LYS  A    234    37.341   42.806   42.101    34.99   C
                                        ATOM    3249    HD1  LYS  A    234    37.041   42.396   43.071    46.01   H
                                        ATOM    3250    HD2  LYS  A    234    36.551   43.346   41.591    37.20   H
                                        ATOM    3251    CE   LYS  A    234    38.561   43.706   42.321    31.70   C
                                        ATOM    3252    HE1  LYS  A    234    39.501   43.336   41.911    39.52   H
                                        ATOM    3253    HE2  LYS  A    234    38.771   43.746   43.391    37.28   H
                                        ATOM    3254    NZ   LYS  A    234    38.261   45.106   42.001    27.91   N
                                        ATOM    3255    HZ1  LYS  A    234    37.401   45.316   42.501    28.82   H
                                        ATOM    3256    HZ2  LYS  A    234    39.011   45.736   42.231    31.12   H
                                        ATOM    3257    HZ3  LYS  A    234    37.991   45.276   41.041    30.42   H
                                        ATOM    3258    C    LYS  A    234    38.391   42.896   38.211    33.66   C
                                        ATOM    3259    O    LYS  A    234    38.811   42.106   37.381    37.81   O
-----------------------------------------------------------------------------------------------

The Hierarchical_All_Atom_PCA Subset coordinates were obtained from: ActiveSite_OmegaLoop.txt
                    The number of residues in the subset = 24
                    The number of atoms in the subset = 376
                    The dimension of the Reference Coordinates matrix  = 1128 by 1
                    The dimension of the Coordinates matrix  = 1128 by 2400
                    The coordinates were aligned to the reference coordinates using quaternion algebra.
                    The atomic RMSFs were calculated and used to create a PDB file with B-Factors replaced with the atomic RMSF.
                    The subset atoms are:
                                        ATOM    710     N    SER  A    70     44.201   41.496   42.341    31.47   N
                                        ATOM    711     H    SER  A    70     44.801   42.306   42.231    37.13   H
                                        ATOM    712     CA   SER  A    70     42.891   41.596   42.941    30.13   C
                                        ATOM    713     HA   SER  A    70     42.951   40.946   43.811    31.46   H
                                        ATOM    714     CB   SER  A    70     42.641   43.066   43.231    37.46   C
                                        ATOM    715     HB1  SER  A    70     41.581   43.276   43.401    50.93   H
                                        ATOM    716     HB2  SER  A    70     42.861   43.676   42.361    54.26   H
                                        ATOM    717     OG   SER  A    70     43.301   43.606   44.361    34.26   O
                                        ATOM    718     HG   SER  A    70     44.251   43.456   44.271    60.98   H
                                        ATOM    719     C    SER  A    70     41.731   40.956   42.191    31.30   C
                                        ATOM    720     O    SER  A    70     40.681   40.776   42.801    45.69   O
                                        ...
                                        ...
                                        ATOM    3238    N    LYS  A    234    36.031   42.406   37.821    36.51   N
                                        ATOM    3239    H    LYS  A    234    36.241   41.856   36.991    38.52   H
                                        ATOM    3240    CA   LYS  A    234    37.021   42.706   38.841    33.93   C
                                        ATOM    3241    HA   LYS  A    234    36.791   43.646   39.331    35.58   H
                                        ATOM    3242    CB   LYS  A    234    37.081   41.606   39.891    34.34   C
                                        ATOM    3243    HB1  LYS  A    234    37.521   40.726   39.421    43.46   H
                                        ATOM    3244    HB2  LYS  A    234    36.061   41.336   40.171    46.43   H
                                        ATOM    3245    CG   LYS  A    234    37.921   41.766   41.151    39.56   C
                                        ATOM    3246    HG1  LYS  A    234    38.981   41.956   40.991    53.48   H
                                        ATOM    3247    HG2  LYS  A    234    37.821   40.876   41.791    54.98   H
                                        ATOM    3248    CD   LYS  A    234    37.341   42.806   42.101    34.99   C
                                        ATOM    3249    HD1  LYS  A    234    37.041   42.396   43.071    46.01   H
                                        ATOM    3250    HD2  LYS  A    234    36.551   43.346   41.591    37.20   H
                                        ATOM    3251    CE   LYS  A    234    38.561   43.706   42.321    31.70   C
                                        ATOM    3252    HE1  LYS  A    234    39.501   43.336   41.911    39.52   H
                                        ATOM    3253    HE2  LYS  A    234    38.771   43.746   43.391    37.28   H
                                        ATOM    3254    NZ   LYS  A    234    38.261   45.106   42.001    27.91   N
                                        ATOM    3255    HZ1  LYS  A    234    37.401   45.316   42.501    28.82   H
                                        ATOM    3256    HZ2  LYS  A    234    39.011   45.736   42.231    31.12   H
                                        ATOM    3257    HZ3  LYS  A    234    37.991   45.276   41.041    30.42   H
                                        ATOM    3258    C    LYS  A    234    38.391   42.896   38.211    33.66   C
                                        ATOM    3259    O    LYS  A    234    38.811   42.106   37.381    37.81   O
-----------------------------------------------------------------------------------------------

The Hierarchical_Heavy_Atom_PCA Subset coordinates were obtained from: ActiveSite_OmegaLoop.txt
                    The number of residues in the subset = 24
                    The number of atoms in the subset = 201
                    The dimension of the Reference Coordinates matrix  = 603 by 1
                    The dimension of the Coordinates matrix  = 603 by 2400
                    The coordinates were aligned to the reference coordinates using quaternion algebra.
```

```
                    The atomic RMSFs were calculated and used to create a PDB file with B-Factors replaced with the atomic RMSF.
                    The subset atoms are:
                                        ATOM    710     N    SER  A    70     44.201   41.496   42.341    31.47   N
                                        ATOM    712     CA   SER  A    70     42.891   41.596   42.941    30.13   C
                                        ATOM    714     CB   SER  A    70     42.641   43.066   43.231    37.46   C
                                        ATOM    717     OG   SER  A    70     43.301   43.606   44.361    34.26   O
                                        ATOM    719     C    SER  A    70     41.731   40.956   42.191    31.30   C
                                        ATOM    720     O    SER  A    70     40.681   40.776   42.801    45.69   O
                                        ...
                                        ...
                                        ATOM    3238    N    LYS  A    234    36.031   42.406   37.821    36.51   N
                                        ATOM    3240    CA   LYS  A    234    37.021   42.706   38.841    33.93   C
                                        ATOM    3242    CB   LYS  A    234    37.081   41.606   39.891    34.34   C
                                        ATOM    3245    CG   LYS  A    234    37.921   41.766   41.151    39.56   C
                                        ATOM    3248    CD   LYS  A    234    37.341   42.806   42.101    34.99   C
                                        ATOM    3251    CE   LYS  A    234    38.561   43.706   42.321    31.70   C
                                        ATOM    3254    NZ   LYS  A    234    38.261   45.106   42.001    27.91   N
                                        ATOM    3258    C    LYS  A    234    38.391   42.896   38.211    33.66   C
                                        ATOM    3259    O    LYS  A    234    38.811   42.106   37.381    37.81   O
----------------------------------------------------------------------------------------------

The Atom_List_PCA Subset coordinates were obtained from: ActiveSite_OmegaLoop.txt
                    The number of atoms in the subset = 131
                    The dimension of the Reference Coordinates matrix  = 393 by 1
                    The dimension of the Coordinates matrix  = 393 by 2400
                    The coordinates were aligned to the reference coordinates using quaternion algebra.
                    The atomic RMSFs were calculated and used to create a PDB file with B-Factors replaced with the atomic RMSF.
                    The subset atoms are:
                                        ATOM    711     H    SER  A    70     44.801   42.306   42.231    37.13   H
                                        ATOM    712     CA   SER  A    70     42.891   41.596   42.941    30.13   C
                                        ATOM    713     HA   SER  A    70     42.951   40.946   43.811    31.46   H
                                        ATOM    714     CB   SER  A    70     42.641   43.066   43.231    37.46   C
                                        ATOM    715     HB1  SER  A    70     41.581   43.276   43.401    50.93   H
                                        ATOM    717     OG   SER  A    70     43.301   43.606   44.361    34.26   O
                                        ATOM    765     CD   LYS  A    73     40.731   42.166   46.391    35.19   C
                                        ATOM    766     HD1  LYS  A    73     40.141   42.506   47.251    58.87   H
                                        ATOM    767     HD2  LYS  A    73     40.601   42.796   45.511    61.17   H
                                        ATOM    768     CE   LYS  A    73     42.181   42.206   46.841    25.37   C
                                        ATOM    769     HE1  LYS  A    73     42.831   41.856   46.031    46.06   H
                                        ATOM    771     NZ   LYS  A    73     42.521   43.606   47.111    14.12   N
                                        ATOM    772     HZ1  LYS  A    73     43.391   43.666   47.621     5.27   H
                                        ATOM    773     HZ2  LYS  A    73     42.821   43.976   46.221    19.59   H
                                        ATOM    774     HZ3  LYS  A    73     41.761   44.146   47.491    28.38   H
                                        ...
                                        ...
                                        ATOM    2379    CZ   ARG  A    178    56.831   32.946   46.171    41.19   C
                                        ATOM    2380    NH1  ARG  A    178    56.781   32.906   47.501    48.55   N
                                        ATOM    2381    1HH1 ARG  A    178    56.381   32.056   47.861    49.58   H
                                        ATOM    2382    2HH1 ARG  A    178    57.521   33.426   47.961    53.49   H
                                        ATOM    2383    NH2  ARG  A    178    57.651   33.766   45.501    41.86   N
                                        ATOM    2384    1HH2 ARG  A    178    57.681   33.676   44.491    36.51   H
                                        ATOM    2385    2HH2 ARG  A    178    58.161   34.436   46.061    48.10   H
                                        ATOM    2389    H    ASP  A    179    50.151   31.686   43.771    16.96   H
                                        ATOM    2392    CB   ASP  A    179    49.561   34.636   43.921    21.05   C
                                        ATOM    2399    O    ASP  A    179    48.661   34.896   41.301    31.17   O
                                        ATOM    3243    HB1  LYS  A    234    37.521   40.726   39.421    43.46   H
                                        ATOM    3245    CG   LYS  A    234    37.921   41.766   41.151    39.56   C
                                        ATOM    3246    HG1  LYS  A    234    38.981   41.956   40.991    53.48   H
                                        ATOM    3247    HG2  LYS  A    234    37.821   40.876   41.791    54.98   H
                                        ATOM    3249    HD1  LYS  A    234    37.041   42.396   43.071    46.01   H
                                        ATOM    3251    CE   LYS  A    234    38.561   43.706   42.321    31.70   C
                                        ATOM    3252    HE1  LYS  A    234    39.501   43.336   41.911    39.52   H
                                        ATOM    3258    C    LYS  A    234    38.391   42.896   38.211    33.66   C
----------------------------------------------------------------------------------------------

Performed Hierarchical_Heavy_Atom_PCA, Computed Top 20 modes.

                    The Eigen Residues and residue PCs were calculated by performing residue pca using a global alignment over the entire subset.
                                        Each residue was represented with the top 3 PCs (DOFs).
                    Number of Residues in the subset: 24
                    Number of Atoms in the subset: 201
                    The KMO Statistic for the variables in the subset is: 0.629948
                    Shrinkage intensity used to shrink the Covariance Matrix = 0.009168
                    Rank of the Covariance Matrix = 72
                    Condition Number of the Covariance Matrix = 1.31E3
                    Trace of the Covariance Matrix = 1.858E2
                    Determinant of the Covariance Matrix = 6.977E-11
----------------------------------------------------------------------------------------------

Performed Hierarchical_All_Atom_PCA, Computed Top 20 modes.

                    The Eigen Residues and residue PCs were calculated by performing residue pca using a global alignment over the entire subset.
                                        Each residue was represented with the top 3 PCs (DOFs).
                    Number of Residues in the subset: 24
                    Number of Atoms in the subset: 376
                    The KMO Statistic for the variables in the subset is: 0.699140
                    Shrinkage intensity used to shrink the Covariance Matrix = 0.009725
                    Rank of the Covariance Matrix = 72
                    Condition Number of the Covariance Matrix = 8.177E2
                    Trace of the Covariance Matrix = 3.94E2
                    Determinant of the Covariance Matrix = 2.636E16
----------------------------------------------------------------------------------------------

Performed RESIDUE PAIR ANALYSIS using Global Alignment for each Residue Pair.
                    Used the top 1 residue PCs (DOFs) to compute the coupling score.
                    No coordinate outliers were adjusted and no thresholding was done.
                    The coupling scores matrix was written to file and plotted as a heatmap in the Residue Pair Analysis Directory in the JEDi output tree.
```

```
-----------------------------------------------------------------------------------------------

Performed Heavy_Atom_PCA, Computed Top 15 modes.

                     Number of Residues in the subset: 24
                     Number of Atoms in the subset: 201
                     The KMO Statistic for the variables in the subset is: 0.975951
                     Shrinkage intensity used to shrink the Covariance Matrix = 0.009735
                     Rank of the Covariance Matrix = 603
                     Condition Number of the Covariance Matrix = 8.417E4
                     Trace of the Covariance Matrix = 2.354E2
                     Determinant of the Covariance Matrix = 0E0
-----------------------------------------------------------------------------------------------

Performed All_Atom_PCA, Computed Top 15 modes.

                     Number of Residues in the subset: 24
                     Number of Atoms in the subset: 376
                     The KMO Statistic for the variables in the subset is: 0.988553
                     Shrinkage intensity used to shrink the Covariance Matrix = 0.011278
                     Rank of the Covariance Matrix = 1128
                     Condition Number of the Covariance Matrix = 1.642E5
                     Trace of the Covariance Matrix = 5.428E2
                     Determinant of the Covariance Matrix = 0E0
-----------------------------------------------------------------------------------------------

Performed Alpha_Carbon_PCA, Computed Top 15 modes.

                     Number of Residues in the subset: 24
                     Number of Atoms in the subset: 24
                     The KMO Statistic for the variables in the subset is: 0.645163
                     Shrinkage intensity used to shrink the Covariance Matrix = 0.006164
                     Rank of the Covariance Matrix = 72
                     Condition Number of the Covariance Matrix = 5.55E3
                     Trace of the Covariance Matrix = 1.492E1
                     Determinant of the Covariance Matrix = 1.736E-113
-----------------------------------------------------------------------------------------------

Performed Backbone_PCA, Computed Top 15 modes.

                     Number of Residues in the subset: 24
                     Number of Atoms in the subset: 96
                     The KMO Statistic for the variables in the subset is: 0.966313
                     Shrinkage intensity used to shrink the Covariance Matrix = 0.006047
                     Rank of the Covariance Matrix = 288
                     Condition Number of the Covariance Matrix = 5.075E4
                     Trace of the Covariance Matrix = 6.349E1
                     Determinant of the Covariance Matrix = 0E0
-----------------------------------------------------------------------------------------------

Performed Atom_List_PCA, Computed Top 21 modes.

                     Number of Residues in the subset: 131
                     Number of Atoms in the subset: 131
                     The KMO Statistic for the variables in the subset is: 0.966611
                     Shrinkage intensity used to shrink the Covariance Matrix = 0.000000
                     Rank of the Covariance Matrix = 393
                     Condition Number of the Covariance Matrix = 2.732E4
                     Trace of the Covariance Matrix = 1.311E2
                     Determinant of the Covariance Matrix = 0E0
-----------------------------------------------------------------------------------------------

Performed Individual Residue PCA using Local Alignment for each residue, Computed Top 20 modes.

                     The results for each residue was put in a separate directory in the JEDi output tree.
-----------------------------------------------------------------------------------------------

The Displacement Vectors (DVs) were calculated using the reference PDB: ss_24_376_AA_RMSF_edited.pdb

The Displacement Vector Projections (DVPs) from the selected PCA models were calculated in the following ways:
                     Standard dot product(dp), normed dp, weighted dp (by sqrt of eigenvalue), and weighted normed dp
                     The weighted DVPs were plotted as 2-D Scatter Plots (PC Plots).
-----------------------------------------------------------------------------------------------

'Intra-Subset' Subspace Analysis was done for each subset comparing the top vector spaces from the selected PCA models.
                     Note that the Hierarchical Methods only use the Covariance PCA model.
                     Key metrics include RMSIP and Principle Angle Spectra.
                     The iterated RMSIPs with comparisons to random were plotted for each analysis with Z-Scores for assessing significance.
                     Additional log files can be found in the /SSA directory tree.
-----------------------------------------------------------------------------------------------

JEDi Analysis Completed: 2019-11-02 18:26:02
                     Total Run Time = 8.204 minutes.
```

B. Sample PDB READ Log file:

```
1A6N.pdb
1A6N_froda_00000001.pdb
1A6N_froda_00000002.pdb
1A6N_froda_00000003.pdb
1A6N_froda_00000004.pdb
1A6N_froda_00000005.pdb
1A6N_froda_00000006.pdb
1A6N_froda_00000007.pdb
1A6N_froda_00000008.pdb
1A6N_froda_00000009.pdb
1A6N_froda_00000010.pdb
1A6N_froda_00000011.pdb
1A6N_froda_00000012.pdb
1A6N_froda_00000013.pdb
1A6N_froda_00000014.pdb
1A6N_froda_00000015.pdb
1A6N_froda_00000016.pdb
1A6N_froda_00000017.pdb
1A6N_froda_00000018.pdb
1A6N_froda_00000019.pdb
1A6N_froda_00000020.pdb
1A6N_froda_00000021.pdb
1A6N_froda_00000022.pdb
1A6N_froda_00000023.pdb
1A6N_froda_00000024.pdb
1A6N_froda_00000025.pdb
```

C. Sample SSA Log File:

```
-------------------------------------------------------------------------------------------
Top_COV_Eigenvectors:
Rows: 150
Cols: 5
Top_CORR_Eigenvectors:
Rows: 150
Cols: 5

Output Directory: C:\\Users\\Charles\\workspace\\JED_2.0\\JED_Test\\Multi\\JED_RESULTS_TEST/cPCA/SSA/CORR_vs_PCORR/
Projections file written to: Projections_dim_5.txt
Cumulative overlaps 1 --> 2 file written to: CO_1_2_dim_5.txt
Cumulative overlaps 2 --> 1 file written to: CO_2_1_dim_5.txt
Principle Angles file written to: PAs_dim_5.txt
Cosine Products file written to: Cosine_Products_dim_5.txt
Vectorial sums of angles file written to: Vector_Sums_of_Angles_dim_5.txt

The Inner Products of each vector in subspace 1 with each vector in subspace 2 are:

     -0.995      0.015     -0.004      0.003     -0.009
      0.016      0.993     -0.002      0.011     -0.028
      0.003      0.001     -0.985     -0.003     -0.014
     -0.004      0.008      0.004     -0.978      0.067
      0.025     -0.040      0.001     -0.059     -0.946


The cumulative overlaps CO_5 for each vector in subspace 1 with all the vectors in subspace 2 are:
Vector  1           0.996
Vector  2           0.994
Vector  3           0.985
Vector  4           0.980
Vector  5           0.949

The cumulative overlaps CO_5 for each vector in subspace 2 with all the vectors in subspace 1 are:
Vector  1           0.996
Vector  2           0.994
Vector  3           0.985
Vector  4           0.979
Vector  5           0.949

The RMSIP score is 0.981

The principle angles (in degrees) are:
PA    1           4
PA    2           6
PA    3           10
PA    4           12
PA    5           19

The cosine products (in degrees) are:
CP    1           4
CP    2           7
CP    3           12
CP    4           16
CP    5           25

The vectorial sums of angles (in degrees) are:
VS    1           4
VS    2           7
VS    3           12
VS    4           17
VS    5           25

Maximum possible angle between two subspaces of this dimension is 201 degrees

Analysis completed: 2016-08-15 07:07:09
```

D. Sample FSSA Iterated Log File:

```
--------------------------------------------------------------------------------------------
Type of analysis: All_Atom
Comparison: Cart_vs_Disp_CORR
First Set of Eigenvectors:
Rows: 120
Cols: 21
Second Set of Eigenvectors:
Rows: 120
Cols: 21
Output Directory: C:\Users\cflcyd\eclipse-
workspace\JEDi_Release2\test\Myoglobin\JEDi_RESULTS_Test_2_Residues\SSA\All_Atom\Cart_vs_Disp_CORR\
Principle Angle Spectra file written to: Iterated_PAs.txt
RMSIPs file written to: Iterated_RMSIPs.txt

SS_DIM      RMSIP       Z-SCORE

1           0.819162    14.16
2           0.775353    57.07
3           0.768154    42.30
4           0.863826    22.01
5           0.858198    43.45
6           0.932900    45.19
7           0.927293    27.64
8           0.891246    21.37
9           0.891031    44.99
10          0.880776    34.91
11          0.878179    96.44
12          0.865819    58.97
13          0.892371    52.38
14          0.888804    43.13
15          0.891796    44.74
16          0.882897    19.57
17          0.888799    32.88
18          0.897940    49.14
19          0.902575    30.95
20          0.910070    33.47
21          0.907081    28.67

The PA spectra for the range of subspaces are:

   35    0    0    0    0    0    0    0    0    0    0    0    0    0    0    0    0    0    0    0    0
   12   60    0    0    0    0    0    0    0    0    0    0    0    0    0    0    0    0    0    0    0
   11   28   82    0    0    0    0    0    0    0    0    0    0    0    0    0    0    0    0    0    0
   10   16   26   57    0    0    0    0    0    0    0    0    0    0    0    0    0    0    0    0    0
   10   12   23   30   67    0    0    0    0    0    0    0    0    0    0    0    0    0    0    0    0
   10   11   14   22   27   34    0    0    0    0    0    0    0    0    0    0    0    0    0    0    0
    9   11   14   15   23   29   41    0    0    0    0    0    0    0    0    0    0    0    0    0    0
    9   10   13   15   16   25   32   75    0    0    0    0    0    0    0    0    0    0    0    0    0
    9    9   11   14   15   19   32   50   55    0    0    0    0    0    0    0    0    0    0    0    0
    8    9   11   14   15   19   23   32   49   74    0    0    0    0    0    0    0    0    0    0    0
    8    8   10   12   13   15   21   26   35   58   74    0    0    0    0    0    0    0    0    0    0
    8    8    9   11   12   14   19   22   26   49   63   89    0    0    0    0    0    0    0    0    0
    7    8    8   11   12   12   16   19   22   26   34   53   88    0    0    0    0    0    0    0    0
    7    7    8    9   11   12   15   17   21   26   34   47   52   67    0    0    0    0    0    0    0
    7    7    8    8   10   11   13   14   18   21   26   32   47   52   79    0    0    0    0    0    0
    7    7    8    8   10   10   11   13   16   17   21   27   35   49   77   80    0    0    0    0    0
    7    7    7    8    9   10   11   12   15   17   19   20   25   30   51   79   84    0    0    0    0
    6    7    7    8    9   10   11   11   14   14   17   18   22   25   30   47   72   87    0    0    0
    6    6    7    8    8    9   10   11   13   14   14   15   19   24   25   29   45   77   83    0    0
    6    6    7    7    8    9   10   11   11   13   14   14   15   19   23   26   30   44   67   81    0
    5    6    6    7    8    8    9   11   11   12   13   14   14   15   19   23   26   40   51   69   76


Analysis completed: 2019-06-10 23:40:21
```

Supplemental Material: JEDi: Java Essential Dynamics Inspector, C. David, C. Avery, and D. Jacobs

E. Sample Plots:

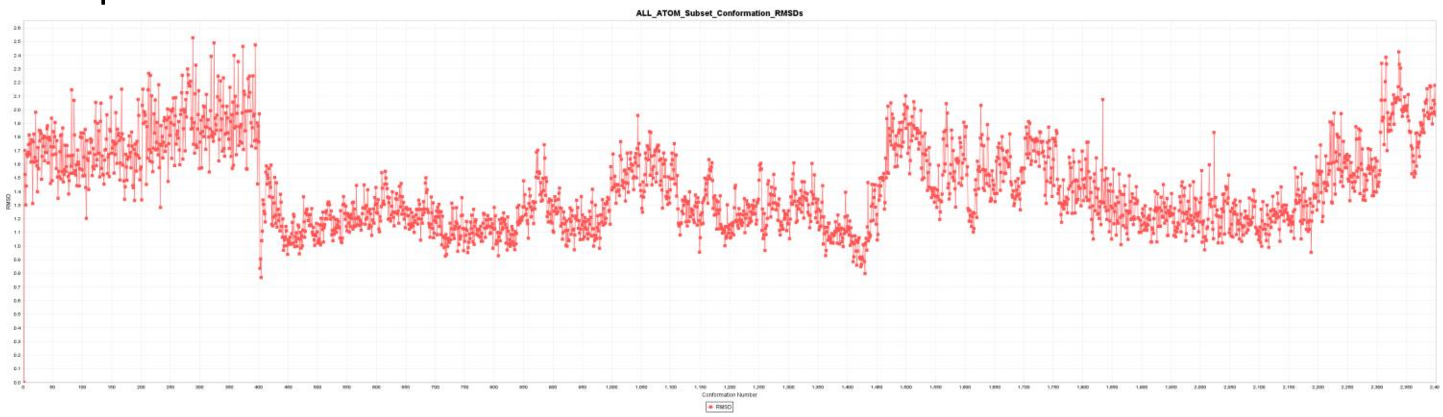

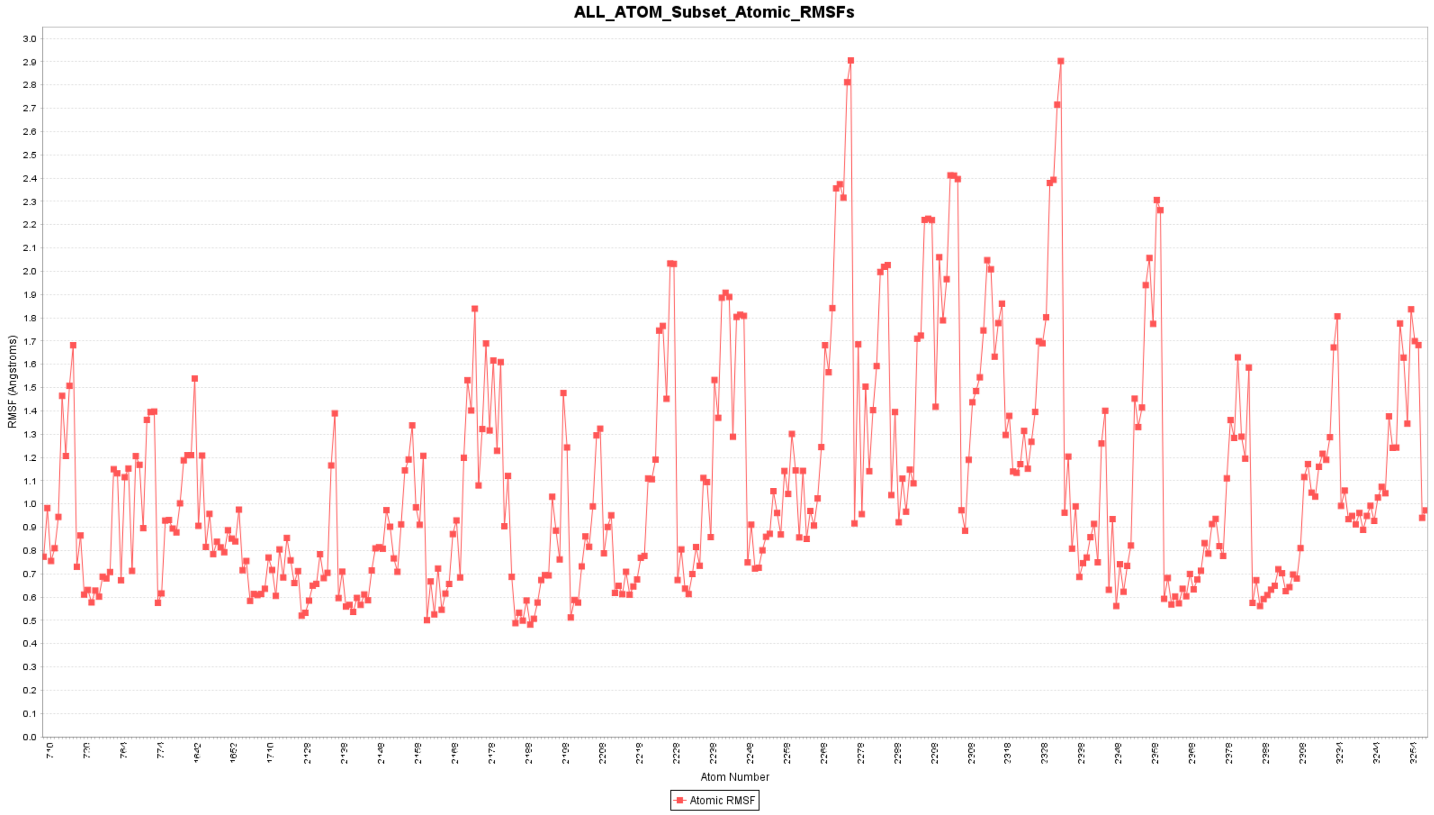

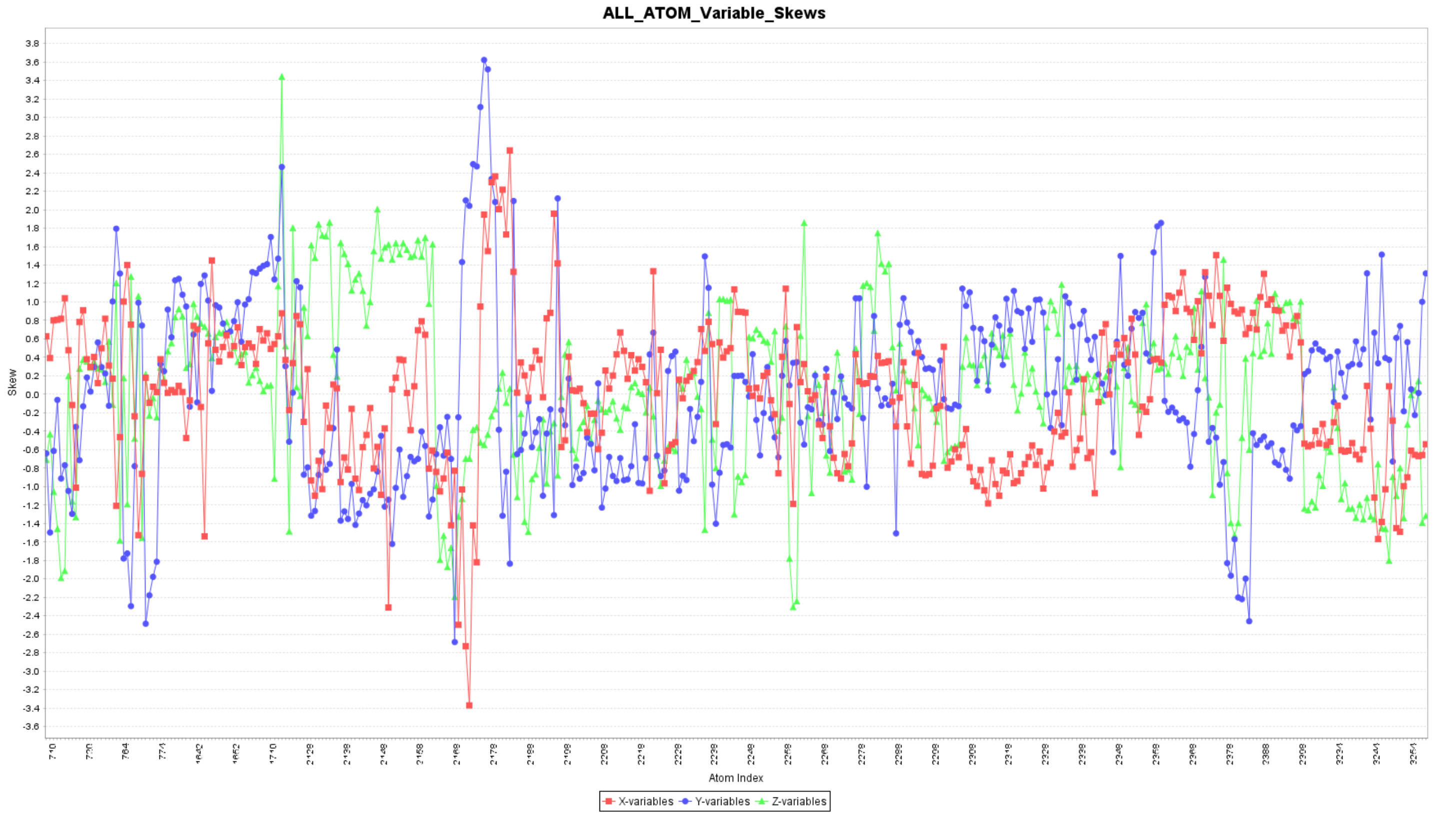
ALL_ATOM_Variable_Skews
Skew
Atom Index
X-variables
Y-variables
Z-variables

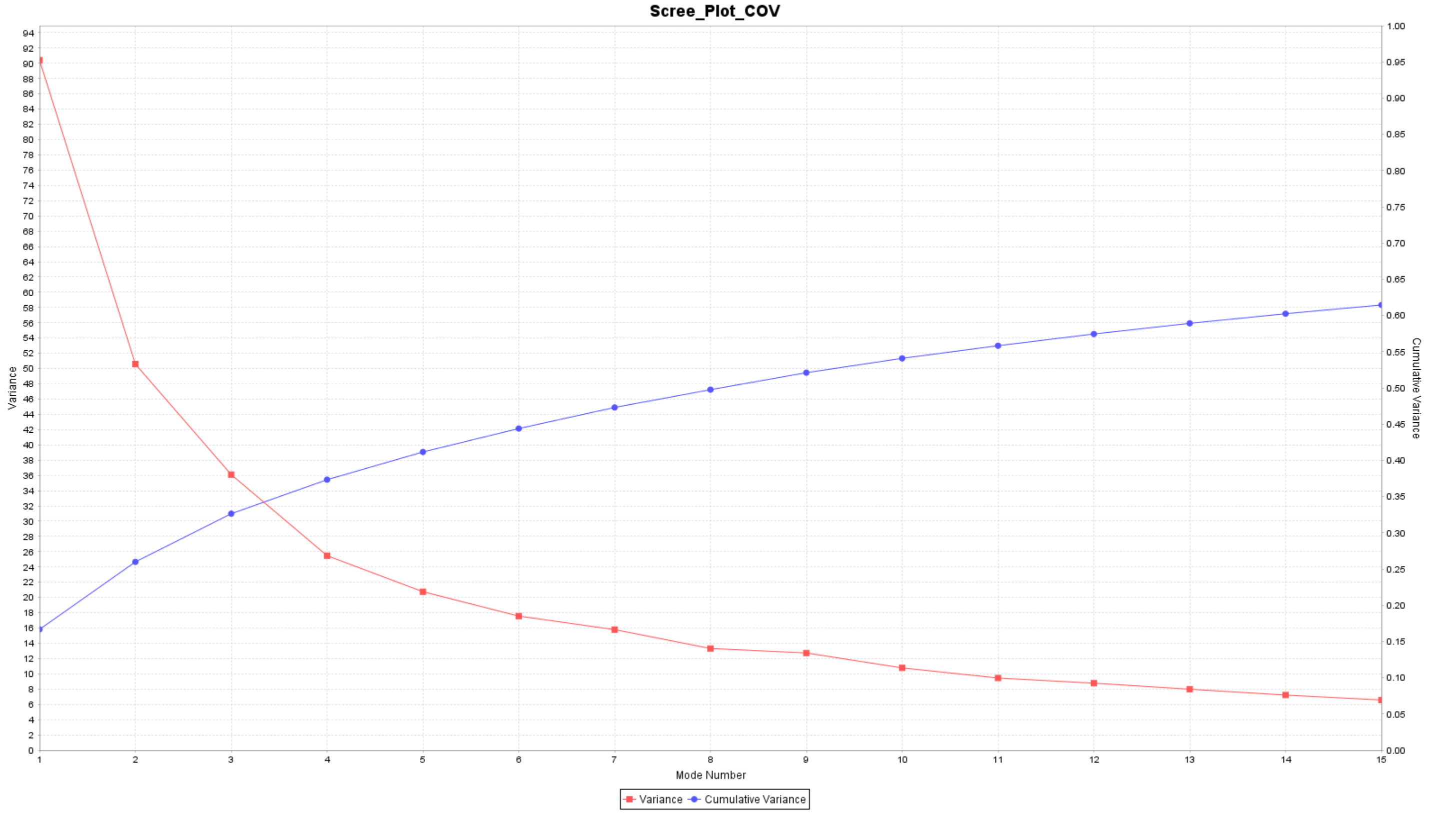
Scree_Plot_COV
Variance
Cumulative Variance
Mode Number
Variance
Cumulative Variance

Supplemental Material: JEDi: Java Essential Dynamics Inspector, C. David, C. Avery, and D. Jacobs

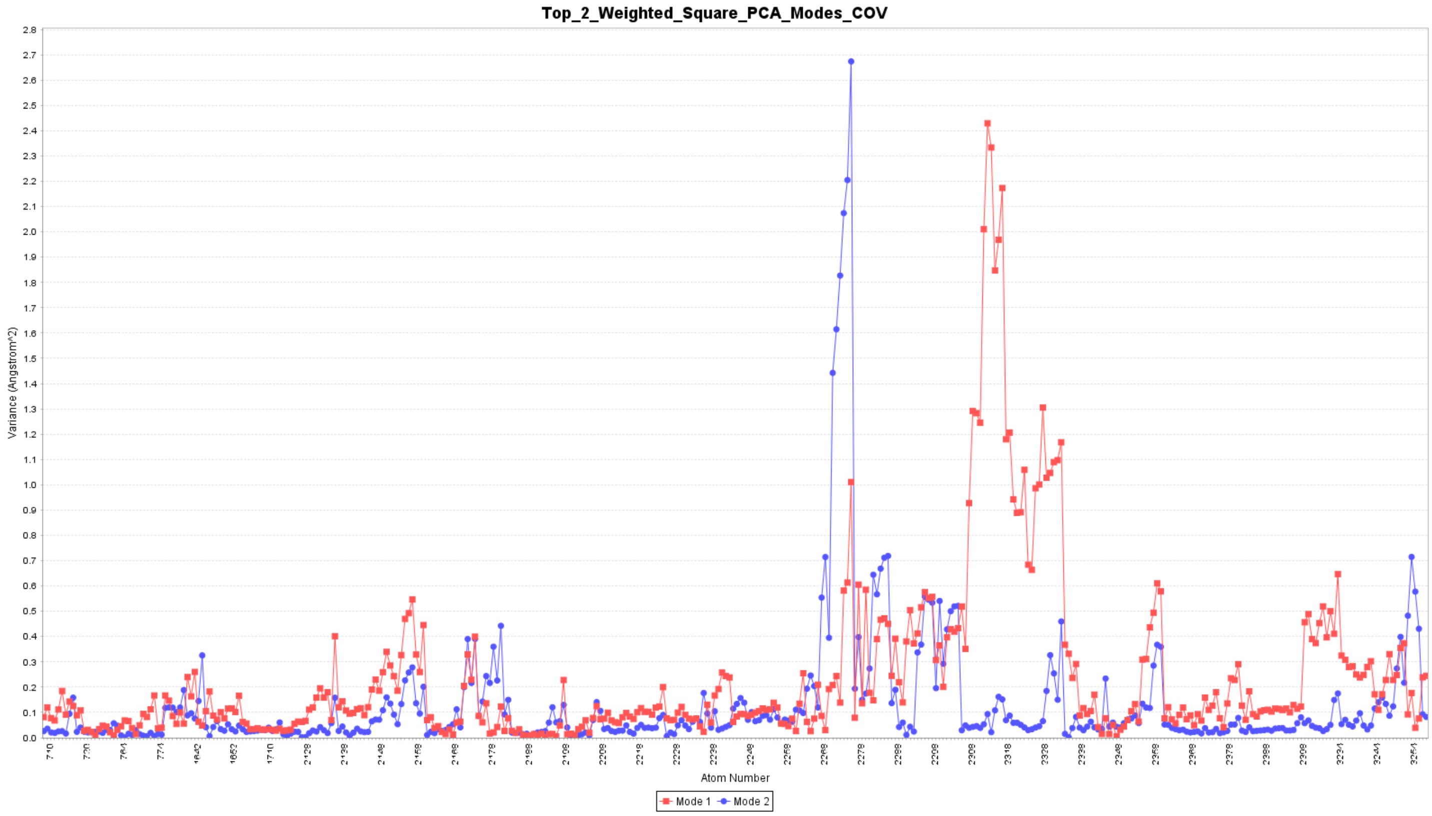

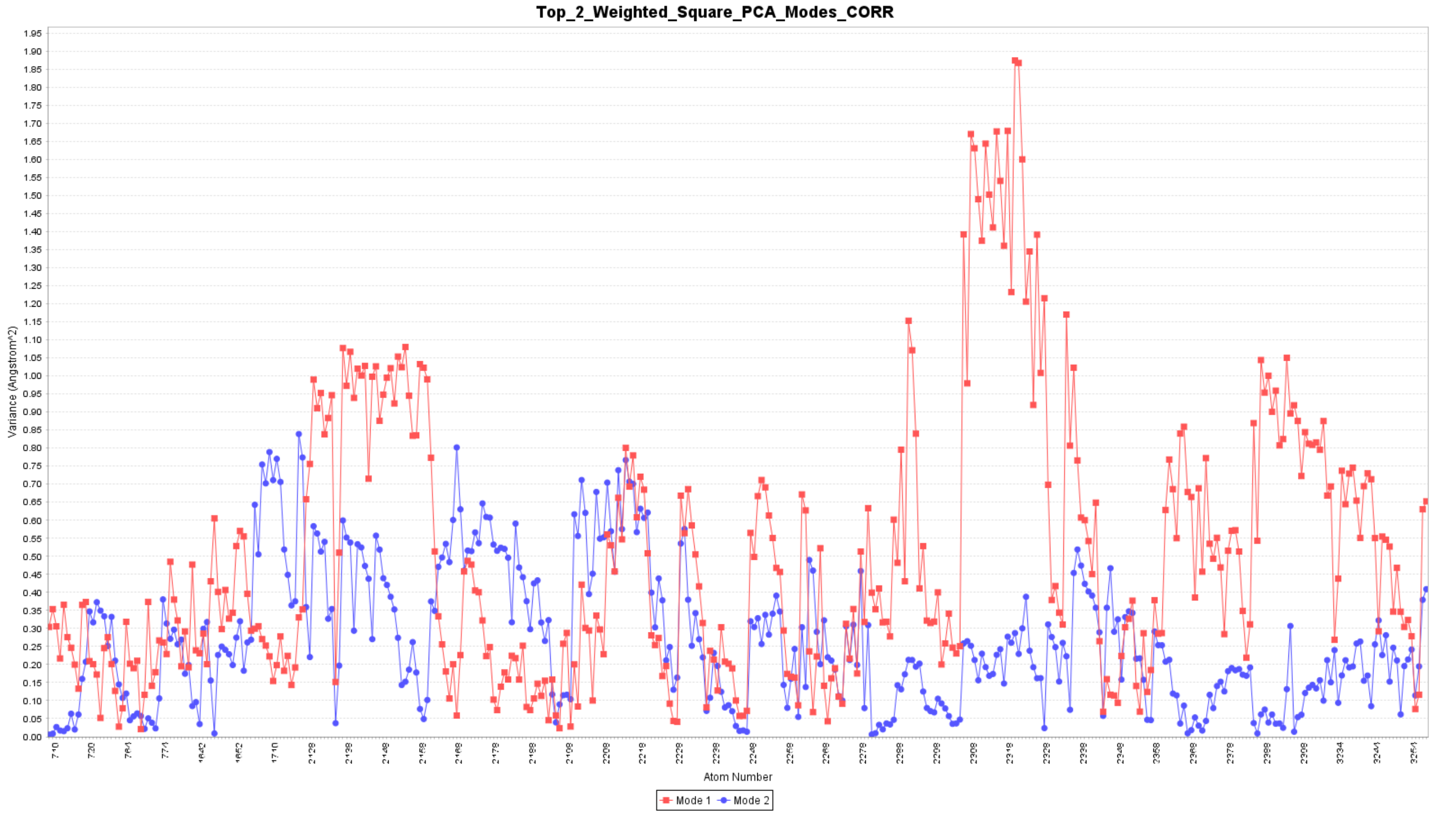

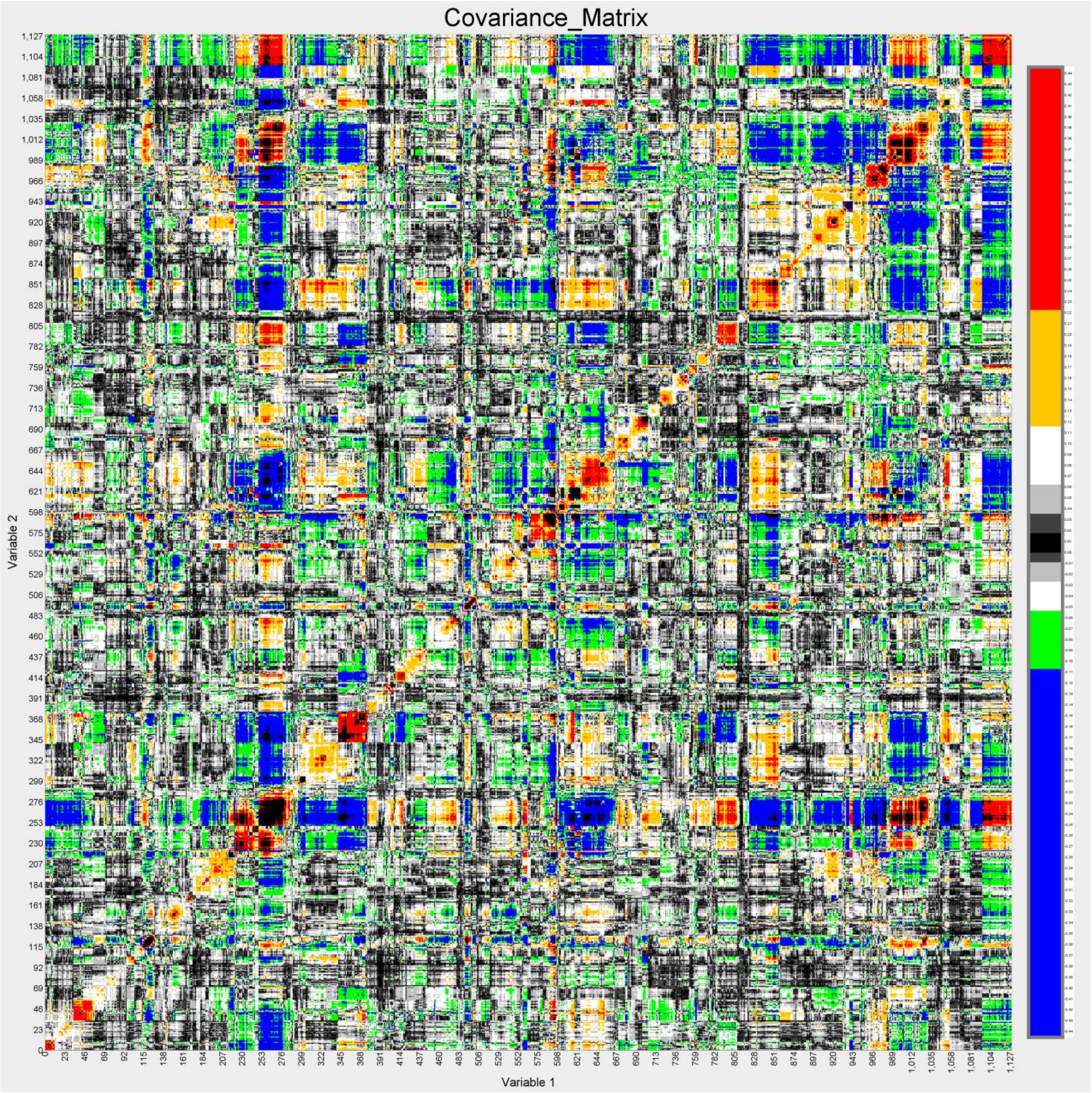
Covariance_Matrix
Variable 2
Variable 1

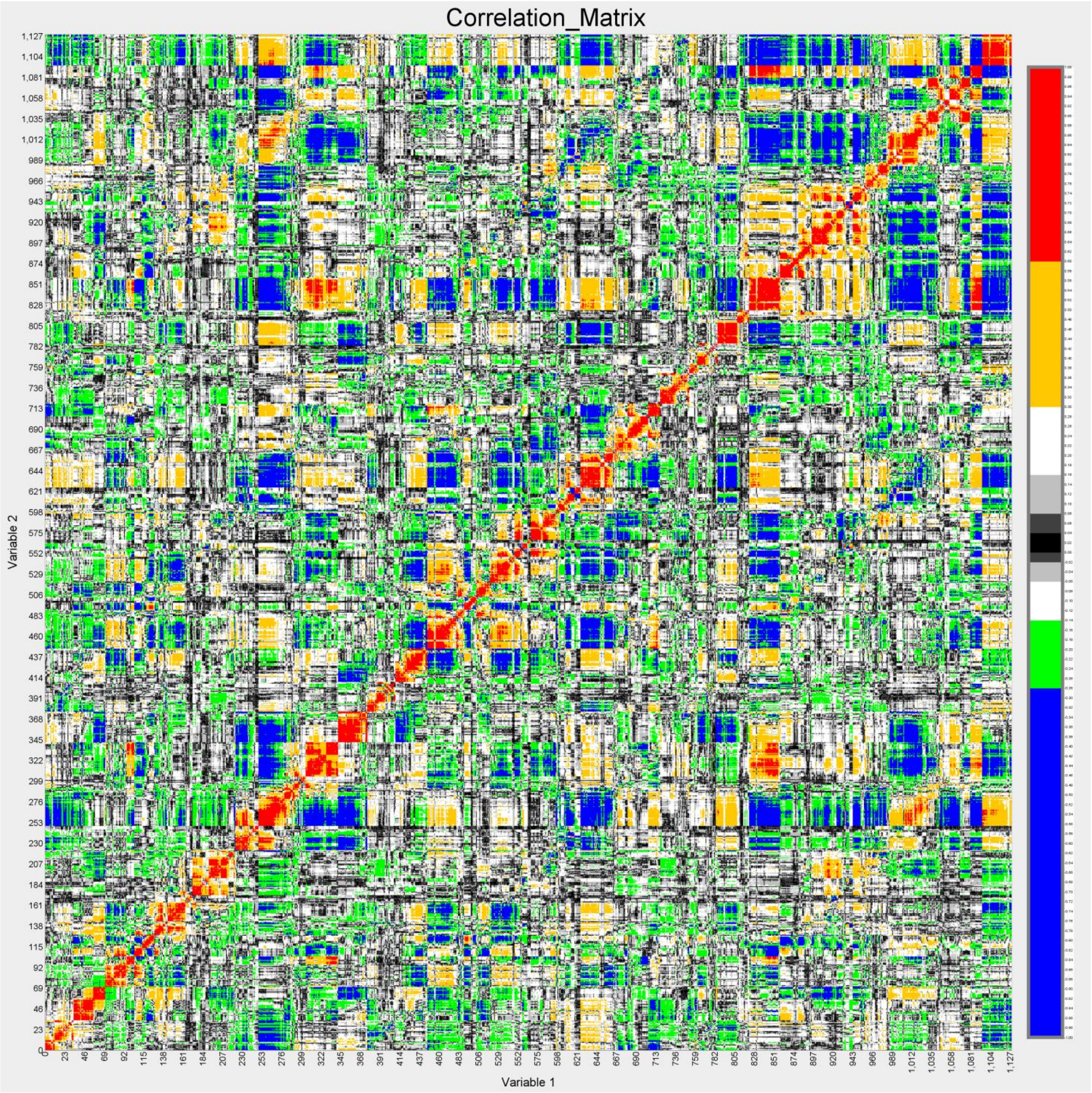
Correlation_Matrix
Variable 1
Variable 2

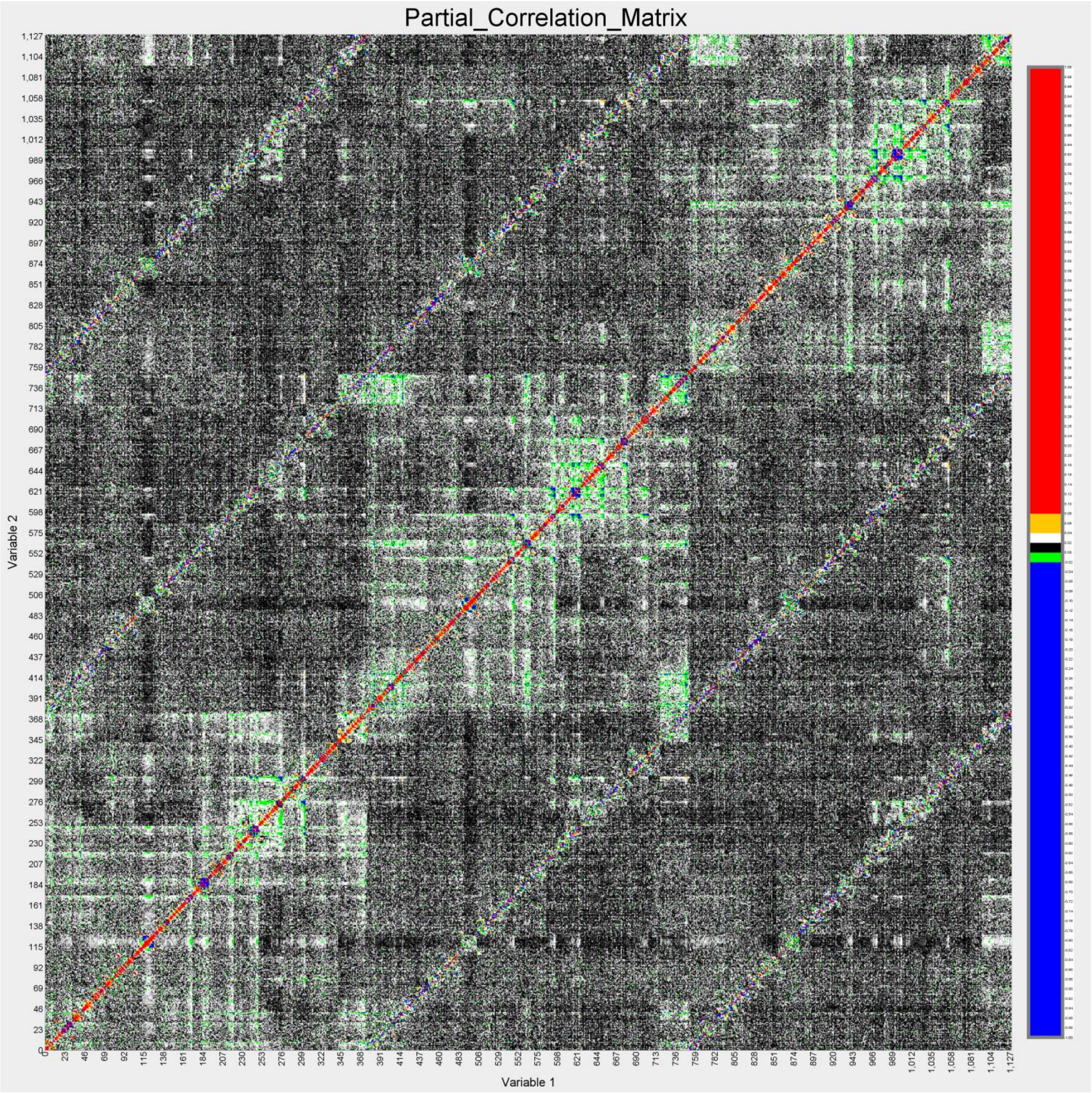
Partial_Correlation_Matrix
Variable 1
Variable 2
0
23
46
69
92
115
138
161
184
207
230
253
276
299
322
345
368
391
414
437
460
483
506
529
552
575
598
621
644
667
690
713
736
759
782
805
828
851
874
897
920
943
966
989
1,012
1,035
1,058
1,081
1,104
1,127

F. Sample PCA Projection Plots:

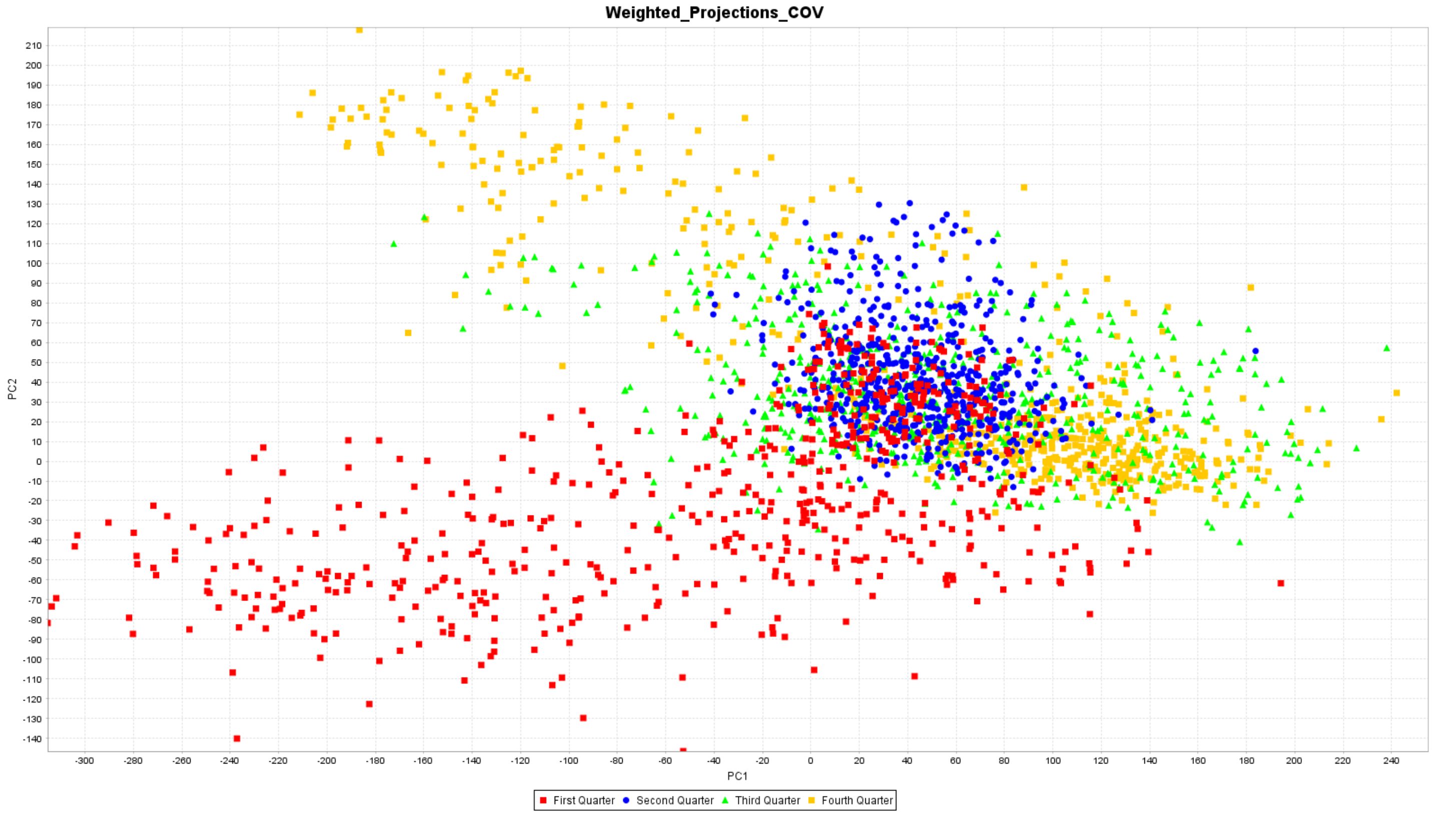

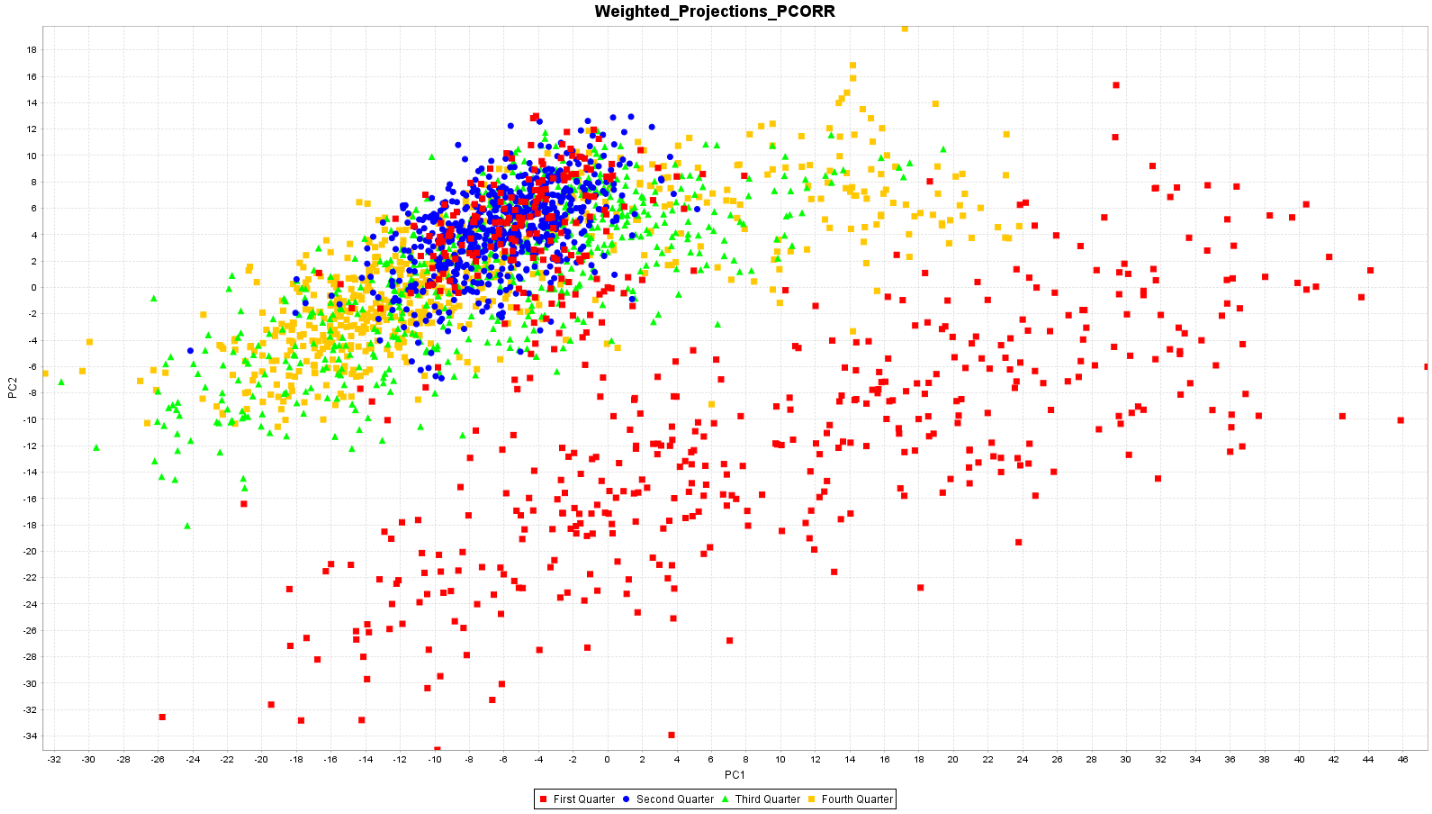

G. Sample Kernel PCA Projection Plots:

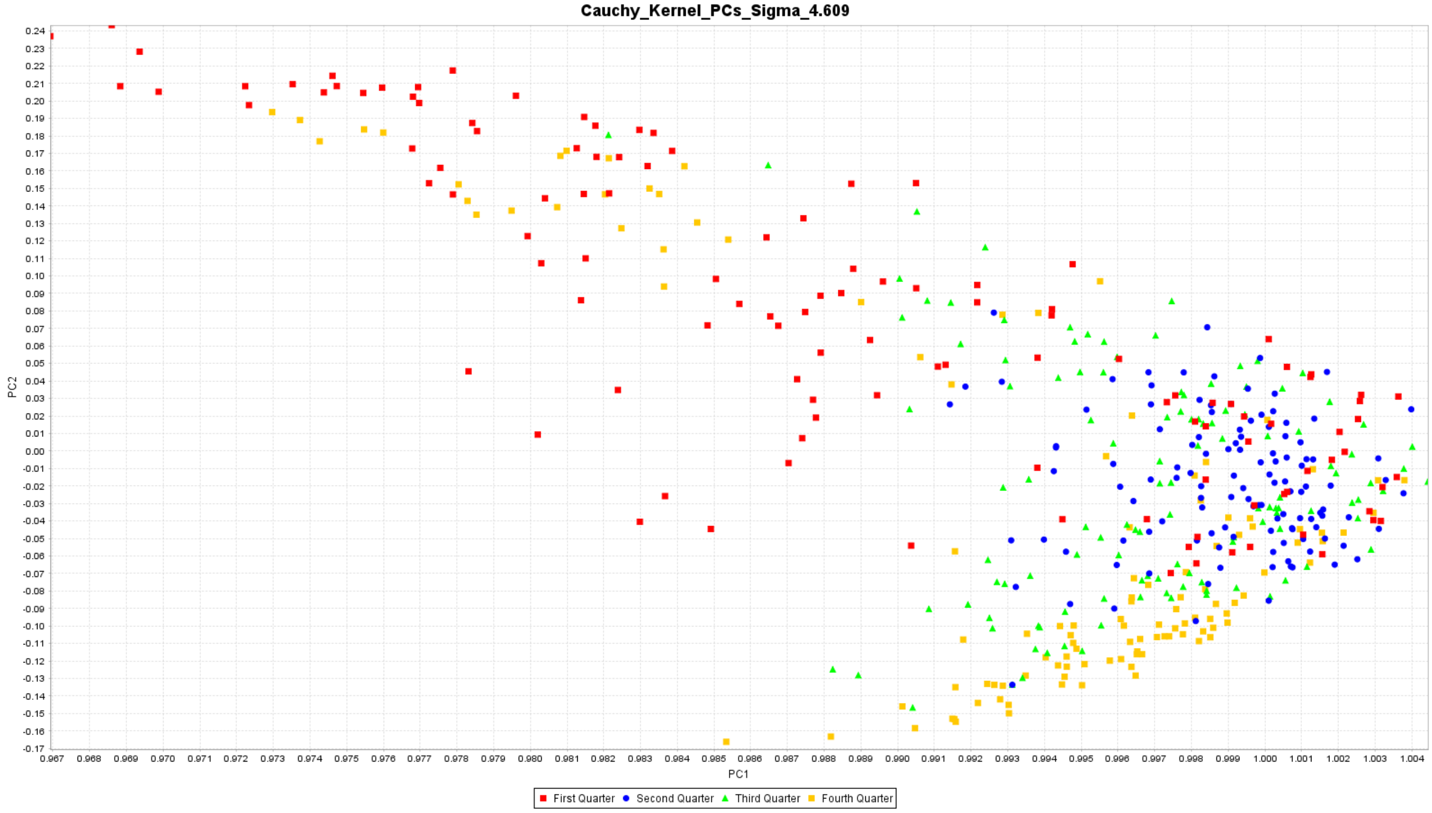

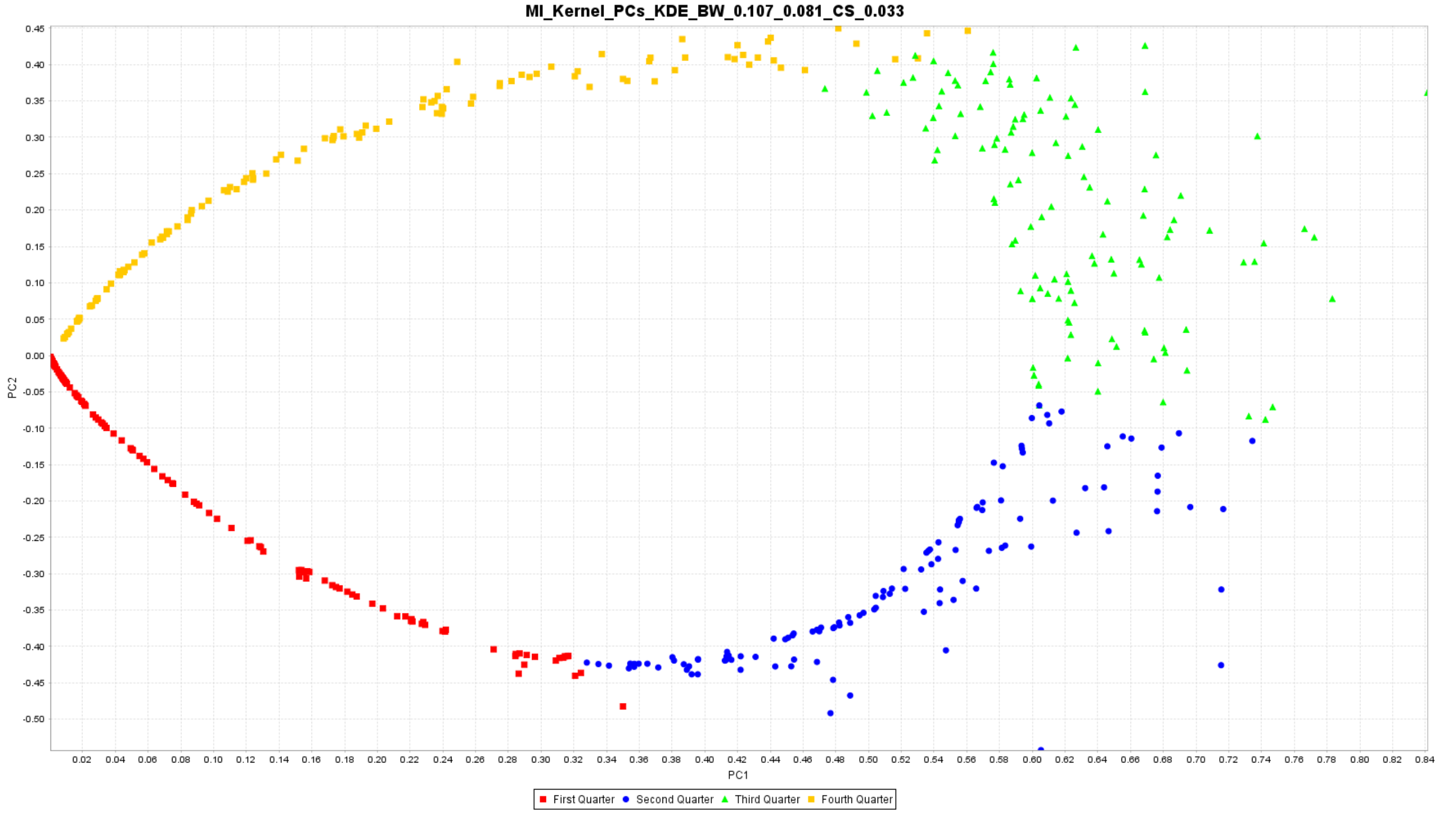

H. Sample FES Plots:

- **24 Residues with 376 Atoms:**

Free_Energy_Landscape_Above_Left

Derived from Normed Projections of PC Modes 1 & 2

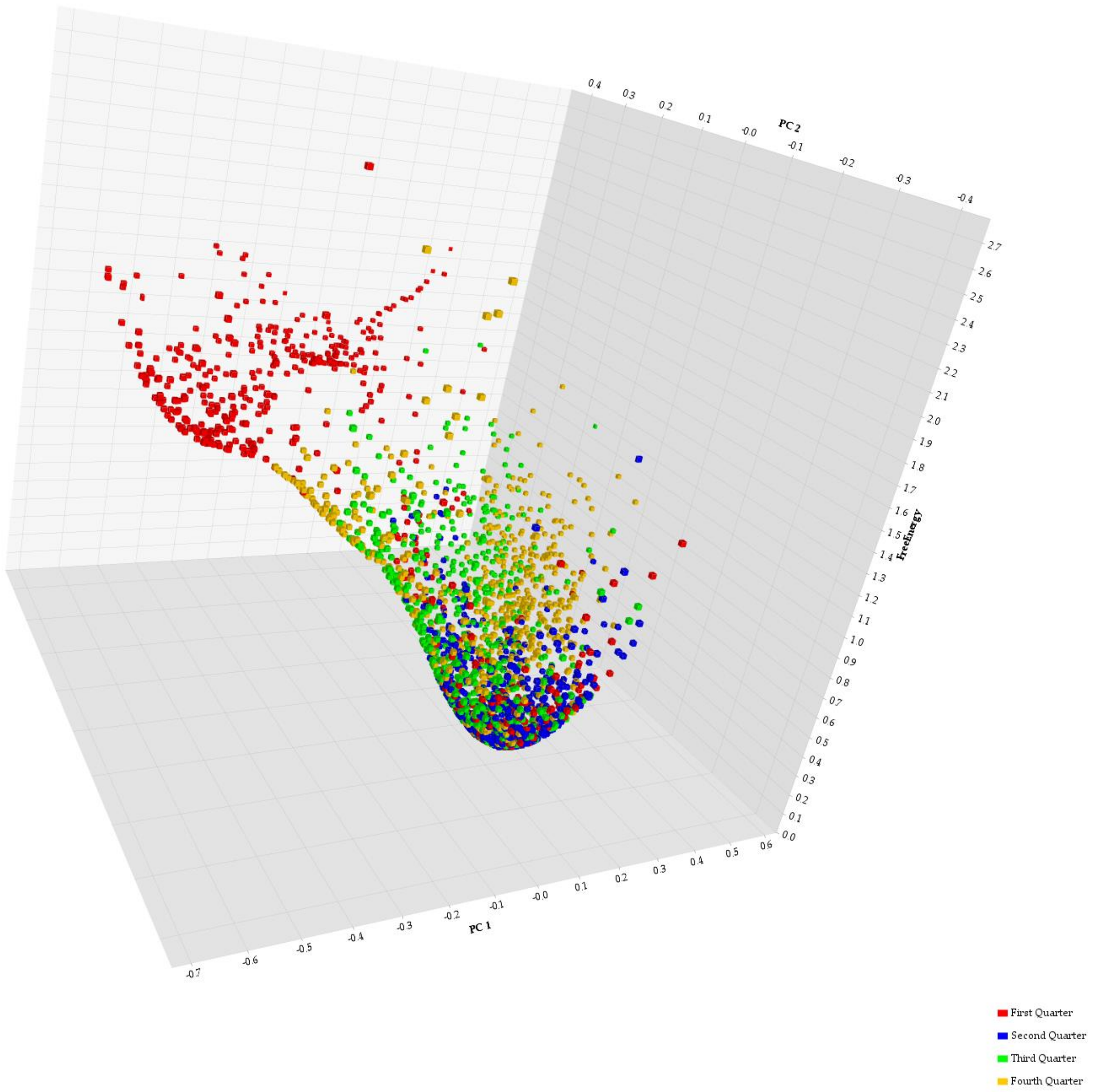

- **Single Residues with 14 Atoms:**

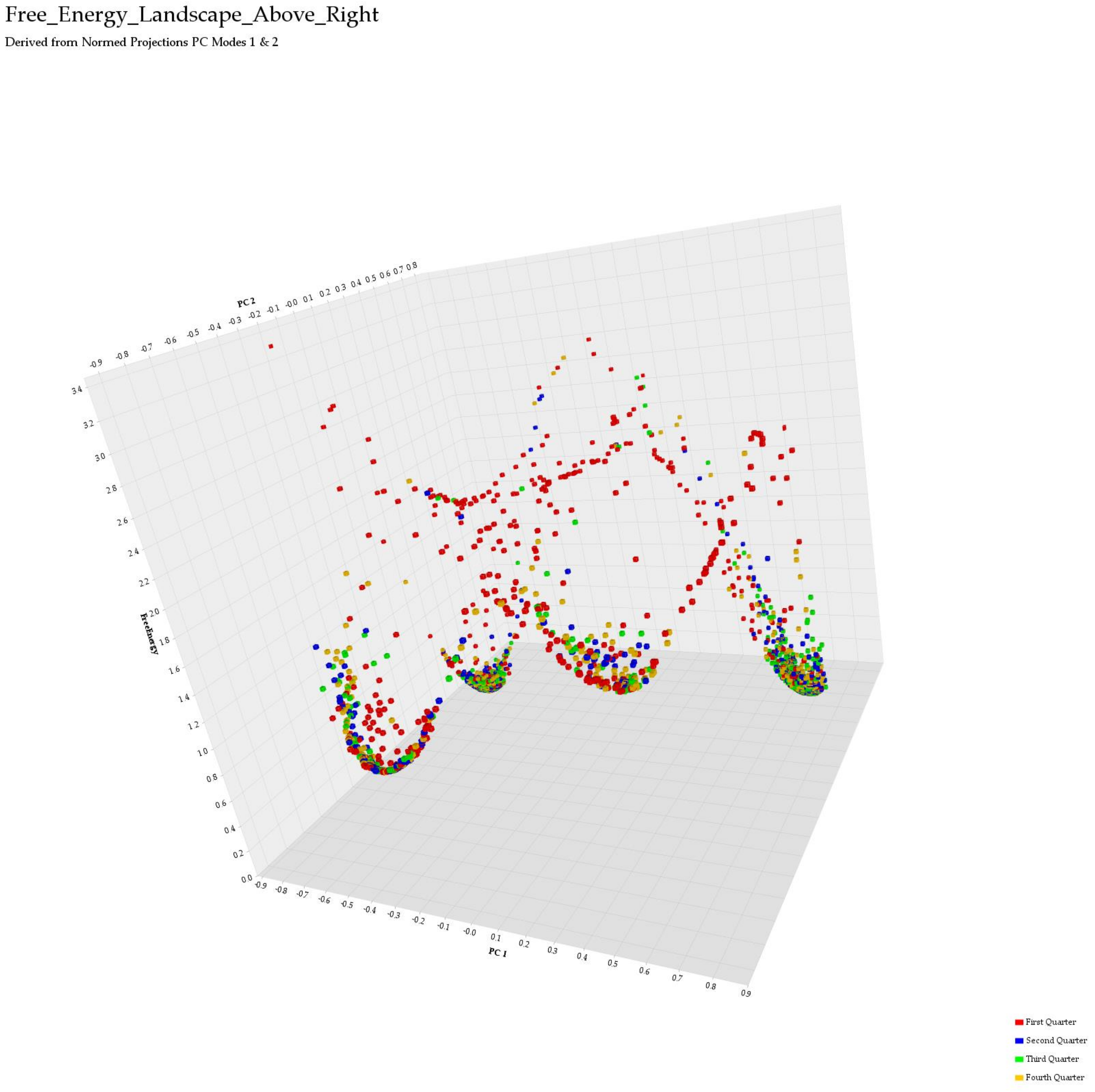

Supplemental Material: JEDi: Java Essential Dynamics Inspector, C. David, C. Avery, and D. Jacobs

I. Sample RMSIP Plots:

➢ COV versus CORR Model

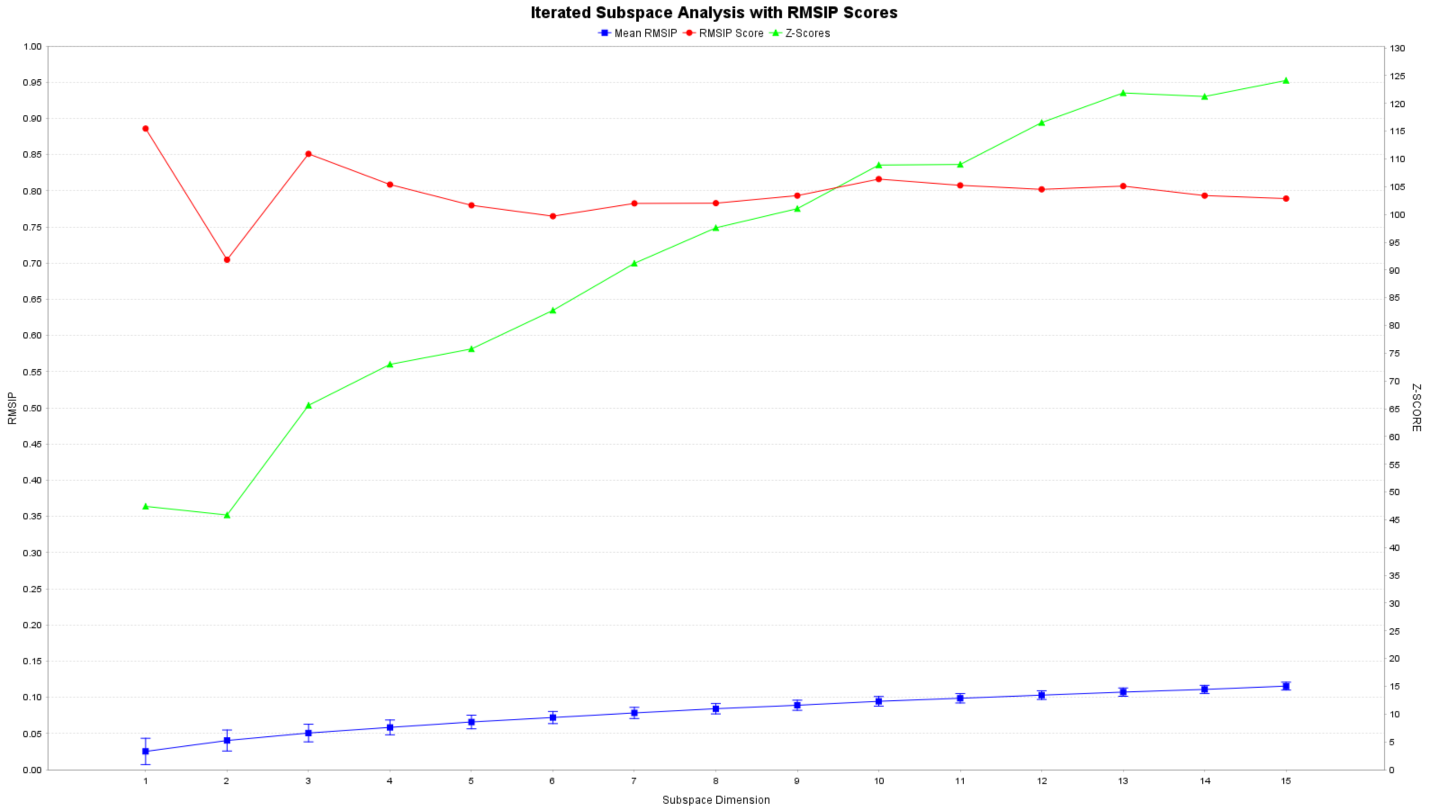

➢ CORR versus PCORR Model

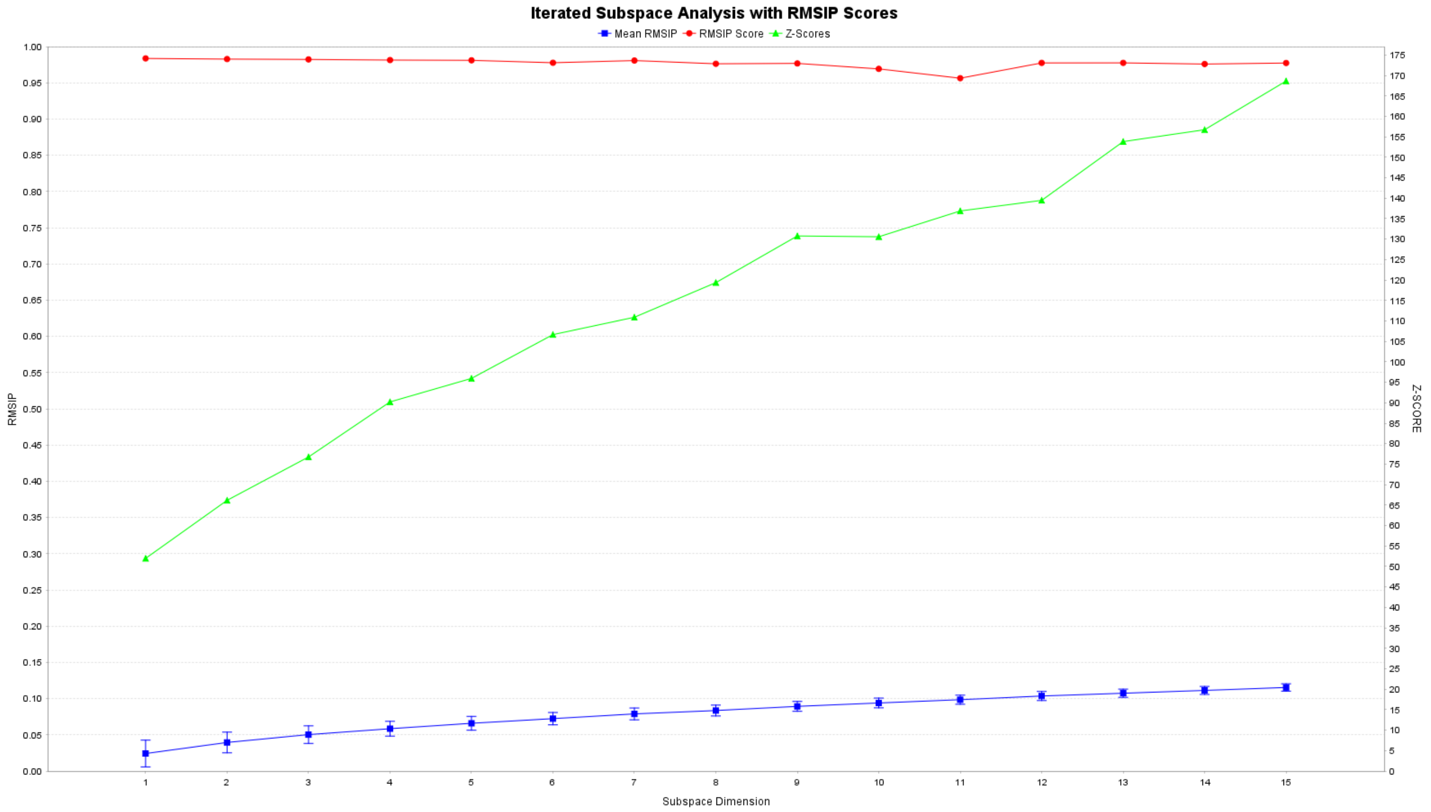

J. **Sample atomic RMSF Edited PDB files:**

- Myosin V: Chains A & C

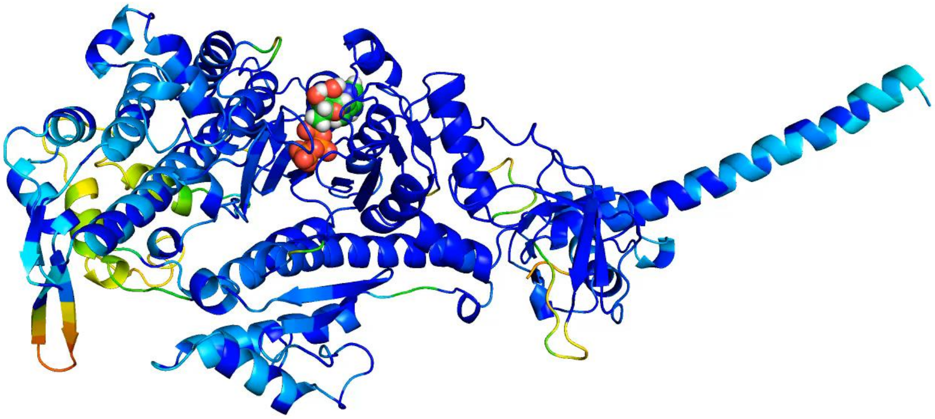

- Beta Lactamase Active Site + Omega Loop

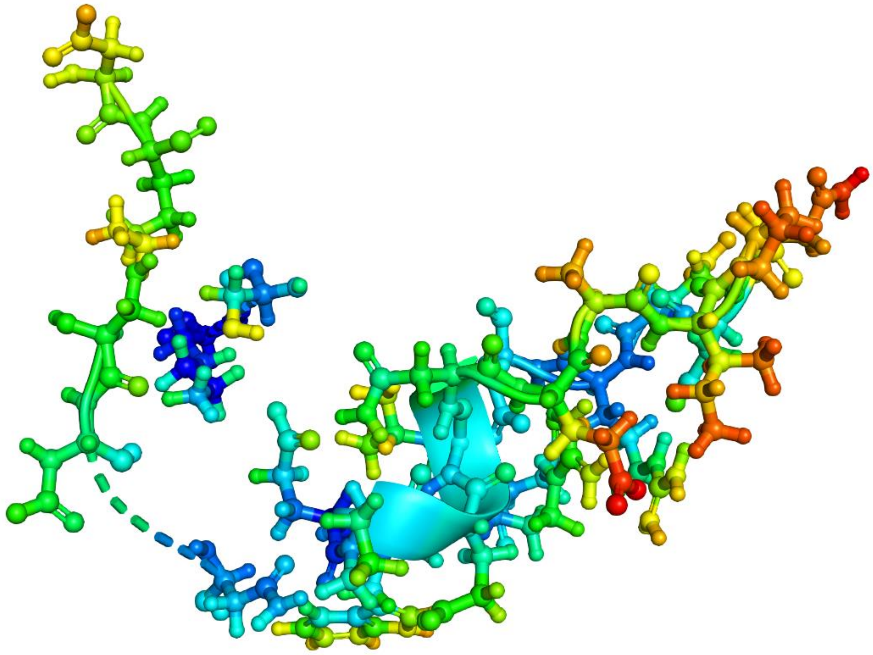

IX. Appendix 3 - For the Impatient...

A. All you need to do a JEDi Pre-Processing Run is a working directory with the following:

- **The JEDi_Parameters.txt** file
- The **reference PDB** file
- All the **PDB files** to be processed

B. All you need to do a JEDi Production Run is a working directory with the following:

- The **JEDi_Parameters.txt** file
- The **reference PDB** file
- The **residue lists**
- The **coordinates file**.

  - You get the all atom coordinates by doing the pre-processing run on your PDB files.

Notes on the parameters file:

- **Please look at Section II D to learn what the parameters control.**
  - **NO SPACES in the KEY=VALUE declarations.**
  - **DO NOT remove or add any key value pairs, they need to be handled programmatically.**
  - **DO NOT have any blank values: no KEY= or JEDi will crash.**
  - **The format of the file NEVER changes, only the VALUES.**
  - **Lines starting with a hash ('#') are ignored**

C. The JEDi Pre-Processing Workflow:

1. Define the working directory (with the PDB files), job description, and reference PDB file: For example,

    - DIRECTORY=workspace/JEDi/test/PDB/
    - DESCRIPTION=TEST_PP
    - REFERENCE_PDB=test.pdb

2. Set the Logical switches for your job: The only relevant ones here are:

    - **doPREPROCESS=true**
    - doReadArchive=true
    - ARCHIVE_NAME=archive.zip
    - READ_PDBS_FILTER_STRING=.pdb

- All other parameters are ignored (but need to be in the parameters file!)

D. The JEDi Production Workflow:

1. Define the working directory, job description, and reference PDB file: For example,

    - DIRECTORY= workspace/JEDi/test/
    - DESCRIPTION=TEST
    - REFERENCE_PDB=test.pdb

2. Specify the all-atom PDB coordinates file: For example,

    - ORIGINAL_PDB_COORDS=original_PDB_Coordinates_AA.txt

3. Set the Logical switches for your job: For example,

    - **doPREPROCESS=false**
    - **doOutlierProcessing=true**

Choose SUBSETS:

- doAA=true
- doHA=true
- doBB=false
- doCA=false
- doRESIDUE_INDIVIDUAL=true
- doRESIDUE_PAIRS= true
- doHIERARCHICAL_AA=true
- doHIERARCHICAL_HA=true
- doATOM_LIST=true
- doATOM_PAIRS= true

Choose PCA MODELS:

- doCORR=true
- doPCORR=true

Choose FEATURES and Related Settings:

- doDownSample=true
- DOWNSAMPLE=10
- doOutlierProcessing=true
- doSPARSIFY=false
- doREDUCE=false
- doKPCA=true
- MAX_KERNEL_FRAMES=250
- doFES=true
- doModeViz=true
- doEssentialViz=true
- MODES_VIZ=10

4. Select the NUMBER OF MODES:

- MODES_RESIDUE_PAIRS=1
- MODES_RESIDUE_INDIVIDUAL=20
- MODES_EIGEN_RESIDUE_AA=3
- MODES_HIERARCHICAL_AA=20
- MODES_EIGEN_RESIDUE_HA=3
- MODES_HIERARCHICAL_HA=20
- MODES_ALL_ATOM=20
- MODES_HEAVY_ATOM=20
- MODES_BACKBONE=20
- MODES_ALPHA_CARBON=20
- MODES_ATOMS_LIST=20
- MODES_DISTANCE_PAIRS=10

5. Specify the residue lists and/or atom list for the analysis you are requesting: For example,

    - RESIDUE_LIST_ALL_ATOM=residues5.txt
    - RESIDUE_LIST_HEAVY_ATOM=residues10.txt
    - RESIDUE_LIST_BACKBONE=residues25.txt
    - RESIDUE_LIST_ALPHA_CARBON=residues100.txt
    - RESIDUE_LIST_LOCAL=residues5.txt
    - RESIDUE_LIST_HIERARCHICAL_AA=residues100.txt
    - RESIDUE_LIST_HIERARCHICAL_HA=residues100.txt
    - ATOMS_LIST= residues.txt
    - ATOM_PAIRS_LIST=atom_pairs.txt

6. Specify Advanced Parameter SETTINGS: For example,

    - VARIANCE_THRESHOLD=1.000
    - SKEW_THRESHOLD=2.500
    - KURTOSIS_THRESHOLD=10.000
    - FLOOR=1.000E-12
    - NOISE_LEVEL=1.000E-3
    - Z_SCORE_CUTOFF=1.960
    - VIZ_MODE_SCALE_FACTOR=0.333

May the Force be with you…Always

END OF HOLOCRON
